# Phenylalanine Modification in Plasma-Driven Biocatalysis Revealed by Solvent Accessibility and Reactive Dynamics in Combination with Protein Mass Spectrometry

**DOI:** 10.1021/acs.jpcb.5c03518

**Published:** 2025-10-23

**Authors:** Hanna-Friederike Poggemann, Sabrina Klopsch, Simon Homann, Tim Dirks, Sina Schäkermann, Julia E. Bandow, Timo Jacob, Christoph Jung

**Affiliations:** † Institute of Electrochemistry, 9189 Ulm University, D-89081 Ulm, Germany; ‡ Applied Microbiology, Faculty of Biology and Biotechnology, 9142 Ruhr University Bochum, D-44801 Bochum, Germany; § Karlsruhe Institute of Technology (KIT), D-76049 Karlsruhe, Germany; ∥ Helmholtz Institute Ulm (HIU) Electrochemical Energy Storage, D-89081 Ulm, Germany

## Abstract

Biocatalysis is an emerging field that provides an environmentally friendly alternative to conventional catalysis but still faces some challenges. One of the major difficulties for biocatalysts that require reactive species like H_2_O_2_ as cosubstrates lies in the concentration of these reactive species. On the one hand, they are used as reactants; on the other hand, they inactivate the enzymes at high concentrations. When utilizing nonthermal plasma to deliver H_2_O_2_ for biocatalysis, it is essential to understand the potential interactions between plasma-generated species (PGS) and enzymes. This is particularly important because, alongside H_2_O_2_, other reactive species such as hydroxyl radicals, atomic oxygen, superoxide, and nitric oxide are also produced. The investigation of the localized reactivity of the solvent accessible surface area (SASA) of an enzyme, with certain species, is an important tool for predicting these interactions. In combination with reactive molecular dynamics (MD) simulations, this enabled us to identify amino acid residues that are likely targets for modifications by the PGS. A subset of the theoretical predictions made in the present study was confirmed experimentally by mass spectrometry, leading to the discovery of plasma-mediated phenylalanine modifications. This result underlines the utility of the SASA and MD-based screening approach to direct time-consuming experiments and assist their interpretation.

## Graphical abstract

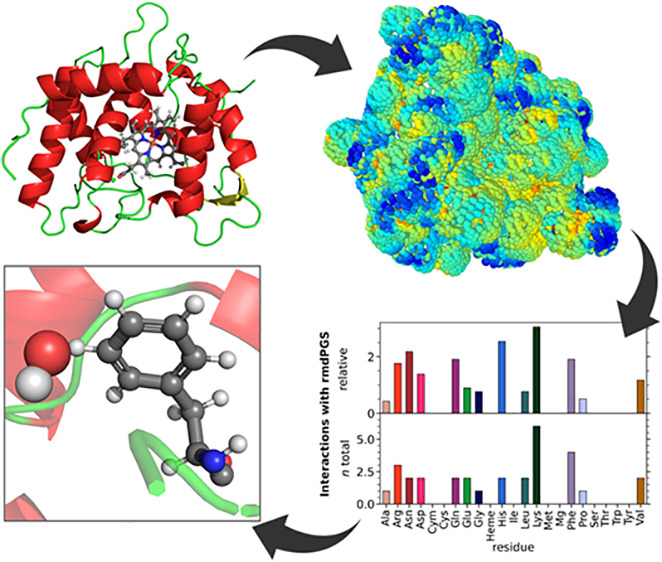

## Introduction

Catalysis plays a pivotal role in our industrial world, as most chemical syntheses only became feasible due to the development of appropriate catalysts. It contributes to the production of about 90% of all industrial chemicals.^1^ However, conventional catalysis often has drawbacks, such as the use of harsh chemicals and the generation of heavy metal waste, for example.

Here, the emerging field of biocatalysis offers several advantages over classical catalysis. Enzymes as catalysts not only exhibit high efficiency but also can show remarkable specificity regarding the substrates and the reactions.^2^ This reduces the need for downstream processing and minimizes waste production. The use of enzymes as catalysts not only benefits the environment but also enables the production of specific chemicals on a smaller scale.^3,4^ To make biocatalysis more competitive than conventional catalytic processes, various strategies have been explored, including the use of individual enzymes and enzyme cascades.^3^ Among the promising emerging enzyme classes are hydrogen peroxide-dependent peroxygenases and peroxidases that catalyze selective oxidative transformations.^5^ In particular, the unspecific peroxygenases (UPOs) have a broad substrate spectrum, and their high chemo-, regio-, and stereoselectivity make them promising candidates for applications in green chemistry and industrial biocatalysis. When using enzymes that work with cosubstrates such as H_2_O_2_, it is essential to ensure a constant supply of the reactive species at suitable concentrations. This is critical, as these species are required to drive the reaction but can cause enzyme inactivation at elevated concentrations, thereby limiting the turnover rates.^6–8^ A recent review from Wapshott-Stehli and Grunden emphasizes that this problem can be addressed by the in situ production of H_2_O_2_ for biocatalysis.^9^ They discuss several approaches in detail, highlighting bioelectrocatalysis as the most efficient way of in situ H_2_O_2_ generation. With this approach, the highest turnover numbers were generated. Another innovative approach to overcome this challenge for H_2_O_2_-dependent enzymes, also mentioned in the review, is plasma-driven biocatalysis.^10^ As the name implies, the method uses plasma to generate H_2_O_2_ as a reactant for the catalytic reaction. Plasma is often referred to as the fourth state of matter, and it is a highly energetic state where the molecules and atoms in a gas are ionized to some extent. This results in a variety of charged species and radicals in the plasma, which can recombine to form new species, such as H_2_O_2_. While most plasmas have a very high temperature, which makes them unsuitable for biocatalysis or other biological applications, nonthermal plasmas are characterized by cooler gas temperatures and are therefore now frequently used in biology and medicine.^11^ For this reason, nonthermal plasma sources are also well suited for the plasma-driven biocatalysis approach. A nonthermal plasma source, such as the capillary plasma jet (CPJ),^12,13^ the microscale atmospheric pressure plasma jet (μAPPJ),^14,15^ or the Cinogy dielectric barrier discharge (DBD) device,^16^ can be used to generate the desired concentration of reactants required for biocatalysis on demand. The reactants are then transported into the liquid phase and therefore to the enzymes via the effluent of the plasma source.^17^ Recent research by Yayci et al. showed that a plasma-driven production of H_2_O_2_ can indeed fuel catalytic reactions.^10,18^ To circumvent the deactivation of the biocatalyst by plasma-generated species, it is crucial to understand the plasma-generated species (PGS)–protein interactions. Previous studies on the interactions between PGS and model peptides and amino acids have shown that the sulfur-containing amino acids cysteine and methionine are most likely to be modified.^19–21^ In their study, Guo et al. described that the strongest structural changes are caused by the introduction of oxygen atoms into the peptides. They also found that polar amino acids have a strong tendency to be oxidized to produce a variety of products. This result agrees with the theoretical findings of Verlackt et al.^22^ who also emphasized that the oxidation of amino acids is influenced strongly by their chemical environment. Furthermore, in their molecular dynamics simulations, Verlackt et al. predicted that the presence of OH radicals mostly leads to H-abstraction and rarely to incorporation of OH into the peptide structure. However, a subsequent experimental study by Wenske et al.^23^ on plasma-induced modification of peptides was unable to confirm all predicted modifications, concluding that steric restrictions might play a major role, particularly in full-size proteins.

The present study seeks to elucidate the interactions between plasma-generated species and three enzymes of interest (see Figure 1) from an atomistic point of view using mainly ReaxFF reactive molecular dynamics (RMD) simulations. We decided to investigate the enzymes *Aae*UPO, *Cvi*UPO, and GapA. *Aae*UPO is a well-studied unspecific peroxygenase and has been the model enzyme for plasma-driven biocatalysis. *Cvi*UPO is a promising alternative candidate; therefore, the focus of this study lies on *Cvi*UPO as the main model enzyme. GapA is neither a nonspecific peroxygenase nor does it use H_2_O_2_ as a cosubstrate. Instead, it is an enzyme involved in central metabolism that is known to be reversibly inactivated by H_2_O_2_ because the active site cysteine is sensitive to oxidation by H_2_O_2_.^25^ This, in turn, enables a comparison between the interaction behavior of an enzyme that is inhibited by H_2_O_2_ and those that actively utilize it. The PGS that occur at high densities in the plasma generated by the ambient air dielectric barrier discharge are H_2_O_2_, ^·^OH, ^·^O_2_^–^, ^·^O, and ^·^NO. Their quantities have been characterized spectrometrically.^26,27^ At this point, it must be emphasized that the species modeled with reactive molecular dynamics are not real plasma-generated species. Due to the limitation of the method, these *reactive molecular dynamics* plasma-generated species, *rmd*PGS, may differ in their chemical behavior from the real species. In general, MD force fields are unable to describe the behavior of excited species, and ReaxFF in particular does not distinguish well between a radical and an ion of the same molecule. This behavior was already observed by Zhang and van Duin in their 2018 study.^28^ Therefore, we refer to the *rmd*PGS in our model only as H_2_O_2_, OH, O_2_, and NO. We expect predominantly radical behavior from the species OH, H, and NO, but it is not impossible that they also exhibit properties associated with ions. A detailed explanation can be found in the Supporting Information. We included H, which is not known to be generated by the plasma effluent, but it could emerge from secondary reactions with the solvent.

**Figure 1:**
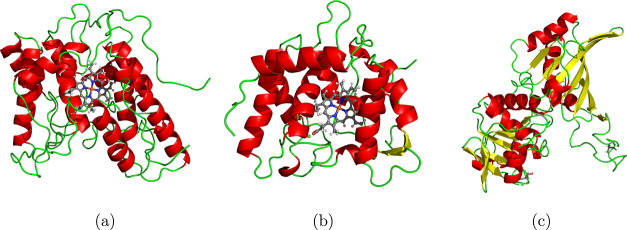
3D structural representations of the enzymes *Aae*UPO (PDB code: 5OXU) (a), *Cvi*UPO (PDB code: 7ZCL) (b), and GapA (PDB code: 7C5H) (c). Helices are shown in red, sheets in yellow, and turns and coils in green. The heme cofactor and the coordinating Mg ions in *Aae*UPO and *Cvi*UPO are shown in atomic representation using the standard atomic color coding (H: white, C: gray, N: blue, O: red, Fe: orange, Mg: green). The 3D representations were created with PyMOL.^24^

In our modeling investigation, the interaction profiles of each species with the enzymes were screened using a solvent accessible surface analysis (SASA) approach. Subsequently, reactive molecular dynamics simulations were performed in order to verify the possible *rmd*PGS-induced modifications of the predicted residues. Furthermore, the role of the surrounding solvent was addressed by performing these MD simulations both without and with the surrounding solvent. Finally, the simulation results were compared to mass spectrometry measurements of plasma-treated *Cvi*UPO, in order to validate the approach against the experimental findings. The experimental analysis concentrates exclusively on *Cvi*UPO, as the glycosylation of *Aae*UPO complicates the mass spectrometric analysis of this enzyme.

## Methods

### Computational Details

All nonreactive MD simulations in this work have been performed with the GROMACS simulation suite (version 2022.2 released June 16, 2022)^29–37^ and the latest version of the CHARMM force field, CHARMM36, for GROMACS (updated July 2022).^38–42^ The nonreactive simulations were used for solvation and pre-equilibration of the enzymes. Prior to the simulations, hydrogen atoms were added to the X-ray structures of the enzymes to emulate neutral pH conditions. Afterward, all proteins were solvated in cubic simulation boxes (80 × 80 × 80 Å) with periodic boundary conditions using the GROMACS solvation procedure and energy minimized and equilibrated by first a short run of 100 ps in a canonical ensemble, in the following referred to as *NVT* ensemble, where *N* is the number of particles, *V* is the volume, and *T* is the absolute temperature. This was followed by a 100 ps simulation in *NPT* (*N*: number of particles, *P*: pressure, and *T*: absolute temperature) ensemble. Both the *NVT* and *NPT* runs had a position restraint on all enzyme atoms but hydrogen. Afterward, a longer MD production run was performed for 1 ns at 300 K or any other desired temperature. The procedure follows the suggestions of Lemkul.^43^ The hence-prepared structures were then used as inputs for the reactive MD simulations.

All RMD simulations have been performed with the latest stable release of the LAMMPS simulation package (version August 2, 2023),^44–47^ employing a ReaxFF potential as originally developed by van Duin et al.^48^ The ReaxFF force field used in this work is the biomolecule force field trained by Monti et al. in 2013.^49^ ReaxFF potentials allow dynamic bond breaking and formation by working with a concept of interatomic distances to determine bond orders, which are then used to compute the potential energy function. The time-integrated molecular dynamics simulations were conducted by using a time step of 0.15 fs.

### SASA Analysis Method

Our SASA analysis package for the interaction analysis is built on the VMD molecular visualization program^50,51^ for the determination of the solvent accessible surface area (SASA) and on the ReaxFF implementation of LAMMPS for the calculation of the interaction energies. The program first automatically converts the GROMACS output file of an equilibrated protein into an LAMMPS data file, deleting the solvent molecule in the process, and then performs an energy minimization with the ReaxFF potential. Afterward, all points on the protein surface that are accessible for the solvent (SASA points) are determined for the minimized structure by an interface to VMD.^50,51^ The distance of the SASA points to the protein surface is set to 1.4 Å per default because it is approximately the radius of water, the most commonly used solvent.^51^ For the calculation of the interaction energy between the protein and a probe molecule, in our case the *rmd*PGS, one probe molecule at a time is then placed at each of the SASA points. All probe molecules are initially rotated so that they are oriented horizontally to the nearest neighbor atoms of the protein. To find the optimal orientation of the probe molecule to the protein, a constrained energy minimization is performed, allowing the probe molecule to rotate, keeping the center of mass at its original position. At last, the program will run a final single-point calculation. The resulting total energy *E*_SASA_ is used to calculate the local interaction energy at a specific SASA point *E*_int_ by subtracting the total energy of the protein *E*_macromol_ and the total energy of the probe molecule *E*_probemol_ (compare eq 1). The latter ones are calculated separately beforehand. All SASA calculations are performed in a vacuum.

$$E_{\mathrm{int}}=E_{\mathrm{SASA}}-E_{\mathrm{macro}\mathrm{mol}}-E_{\mathrm{probe}\mathrm{mol}}$$ 1

The method gives one interaction energy per SASA point, resulting in an interaction map around the enzyme showing regions of strong interactions (most negative values) and regions of weak interactions (fewer negative values). The package further provides two post-processing methods, generating plots showing the protein residues and the atom types that interacted most strongly. The residue analysis includes a calculation of the total interactions per residue and the relative interactions per residue. The relative interactions are calculated as follows:

$$\exp\mathrm{ected}\;\mathrm{Interactions}=\frac{\mathrm{Residue}\;\mathrm{Count}}{\mathrm{Total}\;\mathrm{Residue}\;\mathrm{Count}}\times {\mathrm{Interactions}}_{\mathrm{Total}\;\mathrm{Number}}$$ 2

$$\mathrm{Relative}\;\mathrm{Interactions}=\frac{{\mathrm{Interactions}}_{\mathrm{Residue}}}{\exp\mathrm{ected}\;\mathrm{Interactions}}$$ 3

For our analysis, we defined the relative interactions as the quotient of the observed and expected interactions. By default, the post-processing functions only take into account the 30 strongest interaction energies. This cutoff value was determined empirically by analyzing the average number of data points required to cover the interaction energies of interest. The post-processing functions strongly rely on the Python API of the Ovito Open Visualization Tool.^52^ The SASA package for the interaction analysis is available on GitHub (https://github.com/hpoggemann/SASA-Analysis).

### Enzyme Preparation

The gene *cviUPO* was overexpressed in ZYM-5052 autoinduction medium using a pET21a­(+) expression plasmid containing ampicillin resistance and an N-terminal His-tag.^53^ The plasmid was transformed into *Escherichia coli* BL21 competent cells, and cells were grown in LB medium containing 75 μg mL^–1^ ampicillin at 37 °C overnight. For overexpression, 10 mL of overnight culture were added into 1 L autoinduction medium with 75 μg mL^–1^ ampicillin, 500 μM α-aminolevulinic acid (Roth), and 200 μM hemin (Roth). Overexpression was performed at 16 °C and 120 rpm for 5 days.

Cells were harvested by centrifugation and lysed by a homogenizer (pressure cell homogenizer SPCH-EP, Stansted) in lysis buffer containing 20 mM sodium phosphate, 500 mM NaCl, 10% glycerol, 0.2 mg mL^–1^ DNase (Sigma), 0.2 mg mL^–1^ RNase (Sigma), 0.35 mg mL^–1^ lysozyme (Roth), and 2 mM complete protease inhibitor (Roche). After cell lysis, the lysate was centrifuged at 4 °C and 21,000 × *g* for 60 min, and the supernatant was filtered with 45 and 20 μm cutoff filters. Purification was carried out using the ÄKTA pure 25 system and a 5 mL HisTrap FF crude column (GE Healthcare). Protein elution was performed using 4 column volumes of elution buffer B (20 mM sodium phosphate, 500 mM NaCl, 500 mM imidazole, pH 7.4). Proteins were eluted stepwise at 100 mM imidazole (20%), 200 mM imidazole (40%), and 500 mM (100%) imidazole in buffer B, and fractions were collected in 1 mL steps, while fractions showing an absorption signal at 420 nm were united (heme-loaded protein).

After protein purification, dialysis was performed for buffer exchange in 5 L of buffer A (20 mM sodium phosphate, 500 mM NaCl, 10% glycerol pH 7.4) overnight. Finally, a Bradford assay was used to determine protein concentration and heme loading of the enzyme was measured with UV–vis spectroscopy (V-750 Spectrophotometer, Jasco), calculating the Reinheitszahl (*r*/*z*-value; *A*_420_/*A*_280_) of purified enzyme.

### Plasma Treatment

To assess the influence of plasma on *Cvi*UPO, a dielectric barrier discharge (DBD, Cinogy) source was used for the plasma treatment. To this end, 40 μL enzyme (1 mg mL^–1^ *r* *Cvi*UPO) was placed onto a metal plate and treated with the DBD for 5 min (electrode diameter 20 mm; 13.5 kV pulse amplitude; 300 Hz trigger frequency; 1 mm distance to sample). The treated samples were transferred to a reaction tube for centrifugation (2000 × *g* for 1 min). The samples were stored at −70 °C until mass spectrometric analysis. Untreated protein used as a control underwent the same procedure but was not exposed to plasma.

### Mass Spectrometry of *Cvi*UPO

For mass spectrometry (MS) analysis, the protein samples were reduced, alkylated, and then digested with trypsin. For this purpose, 0.1% RapiGest (Waters) and 2.5 mM tris­(2-carboxyethyl) phosphine hydrochloride were added to a 1 mg mL^–1^ enzyme sample and made up to 60 μL total volume with *A. dest*. The samples were then reduced to 60 °C for 45 min. After the addition of 5 mM iodoacetamide, the samples were incubated again at 25 °C for 15 min in the dark for alkylation. For tryptic digestion, 0.5 μg trypsin (0.5 μg μL^–1^ stock solution) was added prior to incubation at 37 °C and 300 rpm for 5 h. To precipitate RapiGest, 1 μL of trifluoroacetic acid (TFA; concentrated) was added, and the samples were centrifuged at 10,000 × *g* and 4 °C for 10 min. The supernatant was transferred to a new reaction tube, and the procedure was repeated until no more pellets were visible. For the MS measurement, samples were diluted to a concentration of 0.25 μg μL^–1^ in 5% acetonitrile with 0.1% TFA (total volume 20 μL) and 2 μL of sample (0.25 μg μL^–1^) were injected into an ACQUITY UPLC M-Class System (Waters), equipped with a nanoEase *m*/*z* peptide CSH column (Waters), particle size 1.7 μm, column size 0.3 × 100 mm) and eluted online to a Synapt XS (Waters) mass spectrometer equipped with an ESI source and a low-flow probe (Waters). Peptides were eluted using a gradient of 0.1% formic acid (FA) in MS-grade *A. dest.* (solvent A) or acetonitrile (solvent B) with a flow rate of 7 μL/min: 0–3 min, 1% B; 3–100 min, 35% B; 109 min, 90% B; 110 min, 90% B; 115 min, 1% B; and 120 min, 1% B. The column temperature was set to 40 °C, and MS^E^ spectra were recorded from 50 to 2000 *m*/*z* in positive resolution mode with a scan time of 0.7 s. Argon served as a collision gas with a collision ramp of 17–60 V. The following parameters were used: capillary voltage 2.5 kV, cone voltage 40 V, source offset 4 V; cone gas flow 50 L/h, desolvation gas flow 500 L/h, source temperature 80 °C, and desolvation temperature 250 °C. Glu-1-fibrinopeptide B was recorded as lock mass. To analyze the spectra of *Cvi*UPO peptides, ProteinLynx Global Server (Version 3.0.3, Waters) was used to detect protein modifications using an *E. coli* BL21 database (Uniprot UP000002032) containing the sequence for r*Cvi*UPO. The amino acid sequence of the *rCvi*UPO used in this study is provided in the Supporting Information (Figure S1). The His-tag attached to the protein was not considered in the numbering of amino acid positions, resulting in a 21-position shift when compared to the full-length sequence that includes the His-tag. The chromatographic peak width and MS TOF resolution for MS spectra analysis were set to automatic, the lock mass for charge 2 was set to 785.8426 Da/e, and the lock mass window was set to 0.25 Da. The low-energy threshold and elevated energy threshold were specified as 50.0 and 25.0 counts, respectively. Peptide tolerance and fragment tolerance were selected as automatic; minimal fragment ion matches per peptide were 3, minimal fragment matches per protein were 7, and minimal peptide matches per protein were 1. The maximal protein mass was set to 250 kDa, and the primary digest reagent was trypsin. For modification analysis, up to one missed cleavage per peptide was allowed, and the false discovery rate was set to 1. Spectra were processed and searched for the following modifications: carbamidomethylation of cysteine (delta mass +57.0215 Da), which was defined as fixed modifier reagents; oxidation of methionine (delta mass +15.9994 Da), double and triple oxidation of cysteine (delta mass +31.9988 and +47.9982 Da), single oxidation of phenylalanine (delta mass +15.9994 Da), dehydrogenation of arginine (delta mass −4.0316 Da), dehydrogenation of lysine (delta mass −4.0316 Da), oxidative deamination, and dehydrogenation of lysine (delta mass −3.0468 Da). All modifications were defined as variable modifications.

## Results and Discussion

### SASA Interaction Analysis

#### *Cvi*UPO

The SASA interaction analysis was performed for the *rmd*PGS H_2_O_2_, OH, O_2_, O, NO, and H. Figure 2 shows only the results for H, OH, and H_2_O_2_ interactions; data for the other species can be found in the Supporting Information (Figures S2–S5).

**Figure 2:**
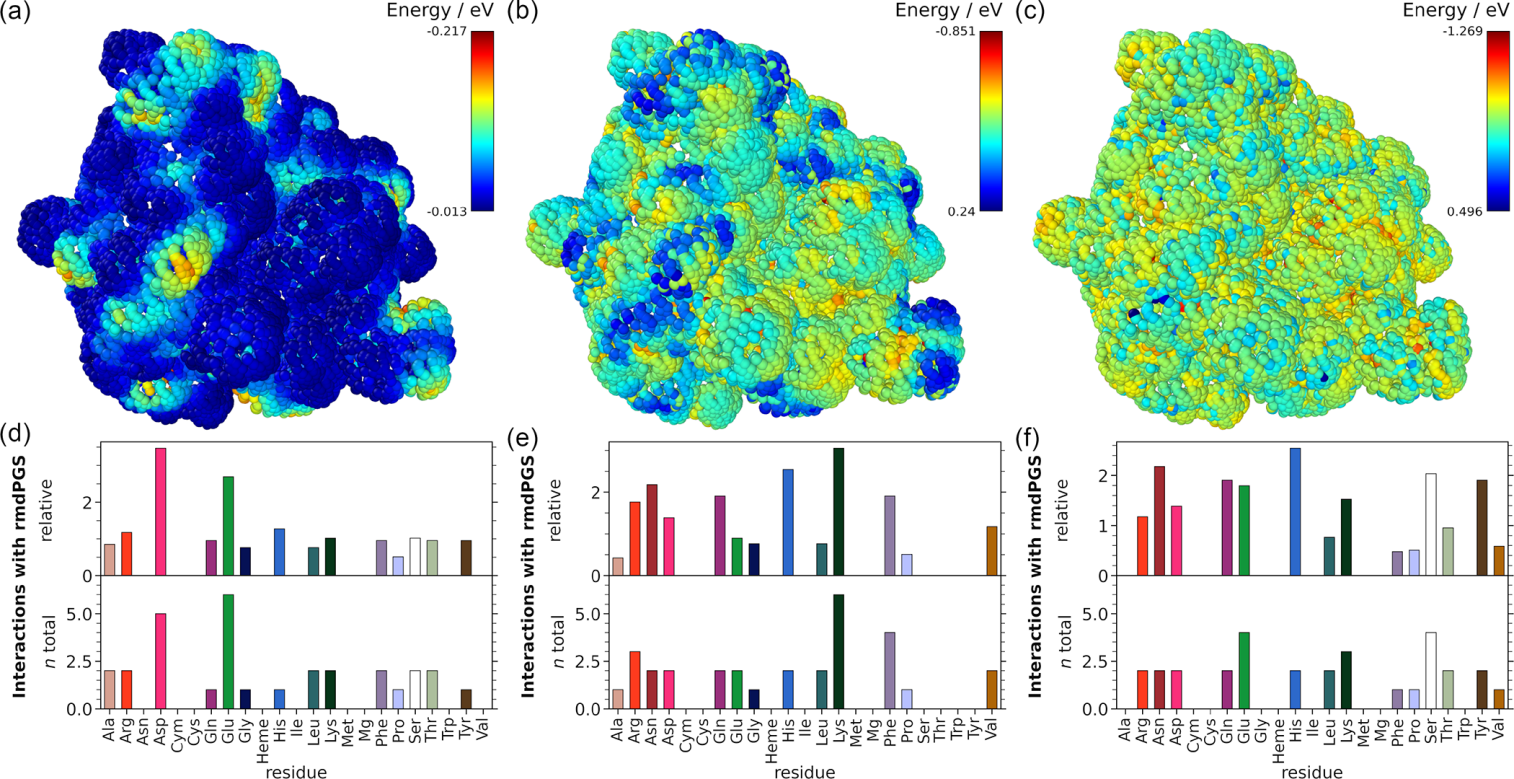
Interaction profiles from the SASA analysis for the enzyme *Cvi*UPO at 300 K. (a), (b), and (c) Interaction maps with H, OH, and H_2_O_2_, respectively. The color bar in the upper right corner of each interaction map indicates the value of the interaction energy for each species. More negative values are indicated in red, and less negative values in blue. The bar plots below show the relative and total interactions per amino acid. The heme cofactor (Heme) and the coordinated Mg ion (Mg) are highlighted separately, as is the cysteine group (Cym) bound to the heme center, which is chemically different from a Cys with a free thiol. (d) Interactions with H, and (e) and (f) interactions with OH and H_2_O_2_, respectively. The computational details provide a detailed explanation of the SASA procedure.

Comparing Figure 2a–c, it is apparent that H has the least negative interaction energy, which means that the interactions with H are weaker than with OH or H_2_O_2_, for example. H shows the strongest interaction with glutamic acid (Glu) and aspartic acid (Asp) residues in both absolute and relative terms (Figure 2d). Both of these residues have unsaturated carboxyl groups and can act as proton acceptor residues.^2^ OH shows the strongest absolute interactions with lysine (Lys), followed by phenylalanine (Phe) and arginine (Arg). When taking into account the frequency of each amino acid occurring in *Cvi*UPO (relative interaction plot in Figure 2e), Lys interactions are the most frequent, followed by histidine (His), Asp, Glu, and Phe. The side chains of the basic amino acids Lys and Arg should be fully protonated at neutral pH, which makes these amino acids attractive for OH and other *rmd*PGS since hydrogen abstraction is commonly observed when modeling the interaction mechanism between PGS and amino acids.^19,22^ Phe is one of the most frequent amino acids in *Cvi*UPO and is also exposed on the outside of the protein, which could explain strong interactions. Also, OH radicals are known to form adducts on the aromatic ring.^54^ OH also shows a nearly inverse local interaction profile to H, which is expected since H mainly interacts with oxygen sites, while OH has most interactions with hydrogen atoms. H_2_O_2_, on the other hand, does display a less locally pronounced interaction profile. Most of the protein surface seems to be equally attractive for this species, except for some sampling points inside the reactive channel. In absolute terms, H_2_O_2_ interacts strongly with serine (Ser) and also with Glu, followed by Lys. When accounting for amino acid frequency (relative values), His, Asn, and Ser interact most frequently with H_2_O_2_. Ser is a polar but uncharged amino acid, and it has proton donor as well as acceptor atoms, which could favor an interaction with an ambivalent molecule, such as H_2_O_2_. O exhibits a similar interaction profile of OH is similar to that of OH, with the highest number of interactions occurring with Lys, followed by Arg (Supplementary Figure S5d). Looking at the relative values, the frequent interactions with Met are particularly striking, as several other studies found Met to be prone to modifications by plasma species.^19–21^ NO interacts strongly with aspartic acid (Asp), which is negatively charged and often acts as a hydrogen acceptor residue (Figure S4d). Furthermore, NO often interacts with Lys, as well as Glu and leucine (Leu). Leu, similar to Phe, has no tendency to act as a hydrogen acceptor or donor; nevertheless, there are frequent interactions with this residue. That can be explained by the fact that Leu is the most frequent amino acid in *Cvi*UPO. The most dominant interaction partner of O_2_ is Lys, followed by Arg, Leu, isoleucine (Ile), tyrosine (Tyr), and valine (Val), all with an equal amount of total interactions in the SASA analysis (see Figure S5a in the SI). Furthermore, O_2_ is the only species besides O that has frequent interactions with Met at 300 K, albeit not quite as often as with O. All tested *rmd*PGS interact strongly with charged or polar residues of the enzyme, which is the expected behavior. But charge is not the only criterion for interaction. The position of the amino acid is also of importance, as is the total frequency of occurrence of the amino acid in the enzyme. Residues such as Leu or Lys, which appear often and are exposed on the outside of the protein, are more likely to have many interactions with *rmd*PGS and are therefore also more likely to be modified under experimental conditions. The analysis shows no strong tendency for the *rmd*PGS to interact with cysteine (Cys) and only O and O_2_ interact with methionine (Met), even though other studies found sulfur-containing amino acids to be strongly modified by the plasma.^19,20,55^ One possible explanation for this behavior might be that neither Met nor Cys appears frequently in *Cvi*UPO (Met only five times and Cys only two times). Also, all seven residues are buried in the protein structure and not at the solvent accessible surface area. Furthermore, there is only one free thiol available because one of the two Cys is bound covalently to the heme center (Cym). In addition, not all species seem to be equally prone to interacting with the active pocket. Only H_2_O_2_ and O_2_ have strong interactions with residues close to the heme center. H_2_O_2_ interacts with a Lys (Lys165) and a Glu (Glu162) residue, while O_2_ interacts with Ile (Ile61), the same Lys residue (Lys165), as well as a hydrogen atom from the heme center. Both the potential removal of a hydrogen atom from the heme center and alterations in Glu162 or Lys165 could be detrimental to the catalytic process. Given that catalysis primarily occurs at the heme center and both of these amino acids play an active role in the catalytic mechanism of *Cvi*UPO, their integrity is crucial for enzymatic function.^56^ In the *Cvi*UPO reaction cycle, Glu162 serves as a hydrogen acceptor, and Lys165 supports the stabilization of the intermediates.

The interaction profiles change when the temperature rises and the protein begins to unfold. The exact denaturation temperatures are different from enzyme to enzyme, but most of them are inactivated at temperatures above 60 °C (338.15 K). Theoretically, higher test temperatures up to 350 K could also be sufficient. However, proteins in MD simulations do not behave identically to those in experiments, and temperatures of 500 K are commonly used in MD simulations when enforcing protein unfolding.^57^ Therefore, alongside 300 K, two higher temperatures—400 and 500 K—were tested, ensuring that the protein is partially unfolded at 400 K and fully denatured at 500 K. The results for OH at 300, 400, and 500 K can be found in Figure 3. For the other species, the results can be found in the Supporting Information (Figures S3–S5). The 3D representations in Figure 3a–c show the stages of protein unfolding. As unfolding increases, so does the SASA, going from 116.803 nm^2^ at 300 K to 119.489 nm^2^ at 400 K and finally 142.758 nm^2^ at 500 K. The bar plots on the right already indicate some differences in behavior at 300 and 400 K. The most notable observation is the decrease in interactions with Lys and Phe at 400 K, accompanied by an increase in interactions with Arg and Asp. This shift may be attributed to the initial stages of enzyme unfolding, which likely expose different amino acids to OH, thereby enhancing the likelihood of interaction. The unfolded protein at 500 K has a greater surface area, exposing even more amino acid residues for potential interactions. But the overall interaction profile of OH is consistent with the lower temperatures. The bar plots highlight that the points with particularly strong interactions do not change significantly with the unfolding of the protein. However, a detailed analysis of all interaction energies revealed that at 500 K, the medium-strength interactions increased dramatically compared to lower temperatures. For the other tested *rmd*PGS spectrometers, this is not necessarily the case. With increasing temperature, most of the *rmd*PGS display a shift in their interactions toward protein residues that have been protected by the surrounding structure before, like the heme center (Supplementary Figures S3–S5 in the SI). Overall, higher temperatures appear to increase the chance of fatal modification on crucial parts of the enzyme as key residues become more exposed to reactive species.

**Figure 3:**
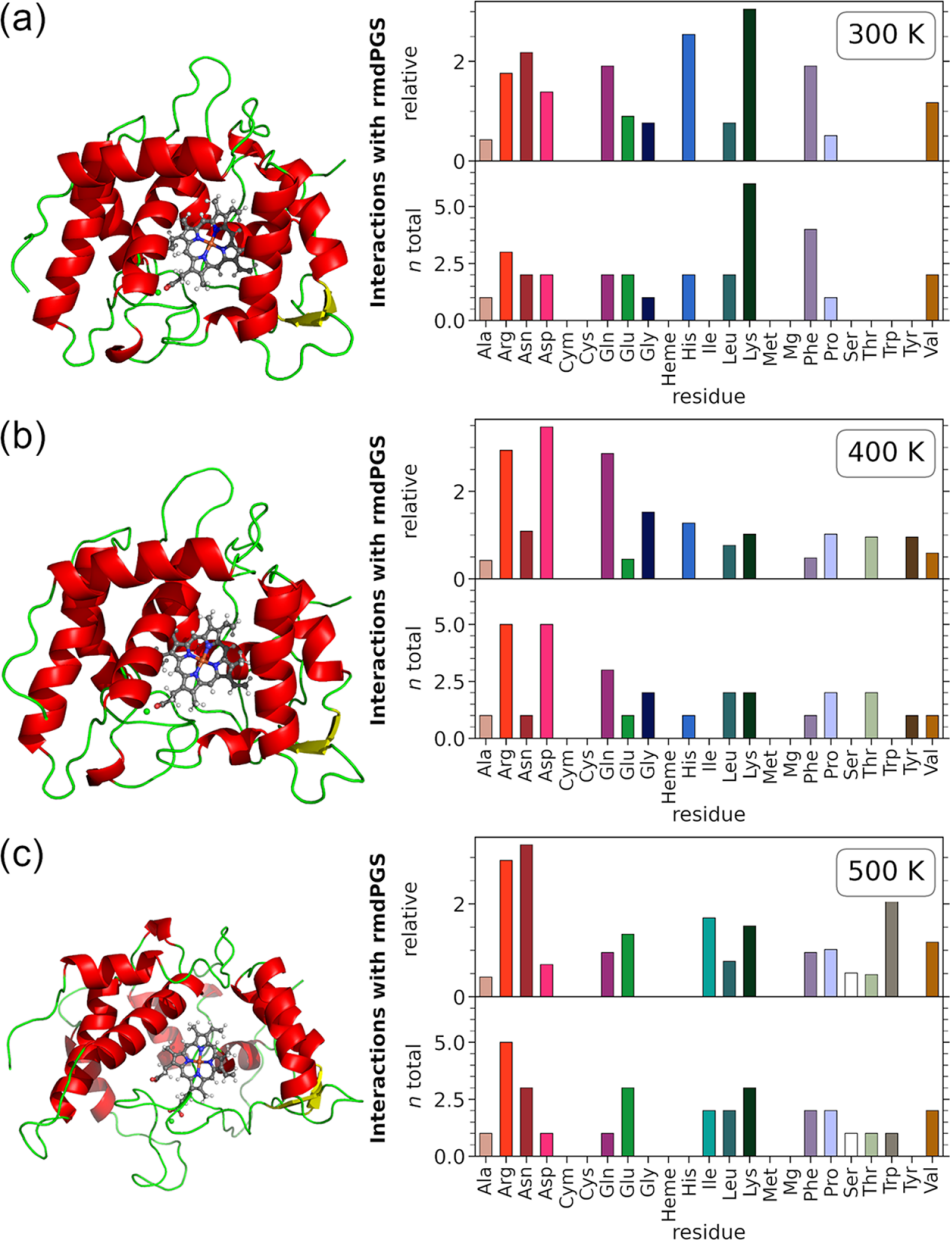
3D representations of *Cvi*UPO (left) and SASA interaction profile (right) for OH at different temperatures at (a) 300 K, (b) 400 K, and (c) 500 K. The SASA procedure and analysis are consistent with those used to generate the previous figure. The bar plots below show the relative and total interactions per amino acid. The heme cofactor (Heme), the coordinated Mg ion (Mg), and the cysteine group bound to the heme (Cym) are highlighted separately. The 3D representations were generated using PyMOL,^24^ with input structures derived from the output of the equilibration simulations at the specified temperatures.

To validate whether the *rmd*PGS really reacts with the protein in the predicted position, those ten SASA points that have the lowest interaction energies were investigated in detail (the precise coordinates and the corresponding amino acids for each point are listed in Supplementary Table S1). After an initial energy minimization, a short MD simulation of 75 fs was performed in order to check whether chemical reactions occur. This brief time period was selected to prevent the probe molecule from diffusing to locations other than the predicted position, ensuring that interactions occur at the predicted sites. The simulations were conducted for all *rmd*PGS at 300 K, 400 K, and 500 K in vacuum as well as in the solvent phase. None of the vacuum simulations showed immediate bond formation between the *rmd*PGS and the protein during the energy minimization step. After 75 fs, mainly H, O, and OH form bonds with the enzyme at the predicted positions with the high interaction energies. The details of interactions can be found in Table S1 in the Supporting Information. The results are consistent at all three tested temperatures. The other *rmd*PGS tend to associate at the protein surface, but they do not form covalent bonds to one of the protein atoms. In the solvent simulations, only a few bonds are forming between the protein and the *rmd*PGS in this time period (compare Table S2 in the Supporting Information). Most of the species do not react with the protein in the predicted position but rather interact with the solvent molecules. This is a trend that also continues with longer simulations that allow diffusion (see the section Interactions of CviUPO with High rmdPGS Concentrations).

This validation shows that the algorithm is indeed able to predict the positions of the protein surface that are most likely to be modified by *rmd*PGS binding, but a high interaction energy at a particular position does not automatically mean that *rmd*PGS will bind there. A subsequent verification is always recommended.

#### Comparison to *Aae*UPO and GapA

To broaden the scope of our investigation, the interaction analysis was also performed for *Aae*UPO, which is a model enzyme for H_2_O_2_-dependent biocatalysis, and for GapA. GapA is a glycolytic enzyme that does not have a heme center but uses a cysteine thiol group as its catalytic center instead. While H_2_O_2_-based heme poisoning is a common problem of peroxidases, GapA is inactivated reversibly in an H_2_O_2_-dependent fashion by the formation of an intramolecular disulfide bond that engages the catalytic cysteine. Figure 4 shows a comparison of the OH interactions with the three enzymes. Similar to Figure 2, the interaction maps are displayed in the upper panels, and the interactions with amino acids are in the lower panels. The interactions of the other tested species can be found in the Supporting Information (Figures S6–S9).

**Figure 4:**
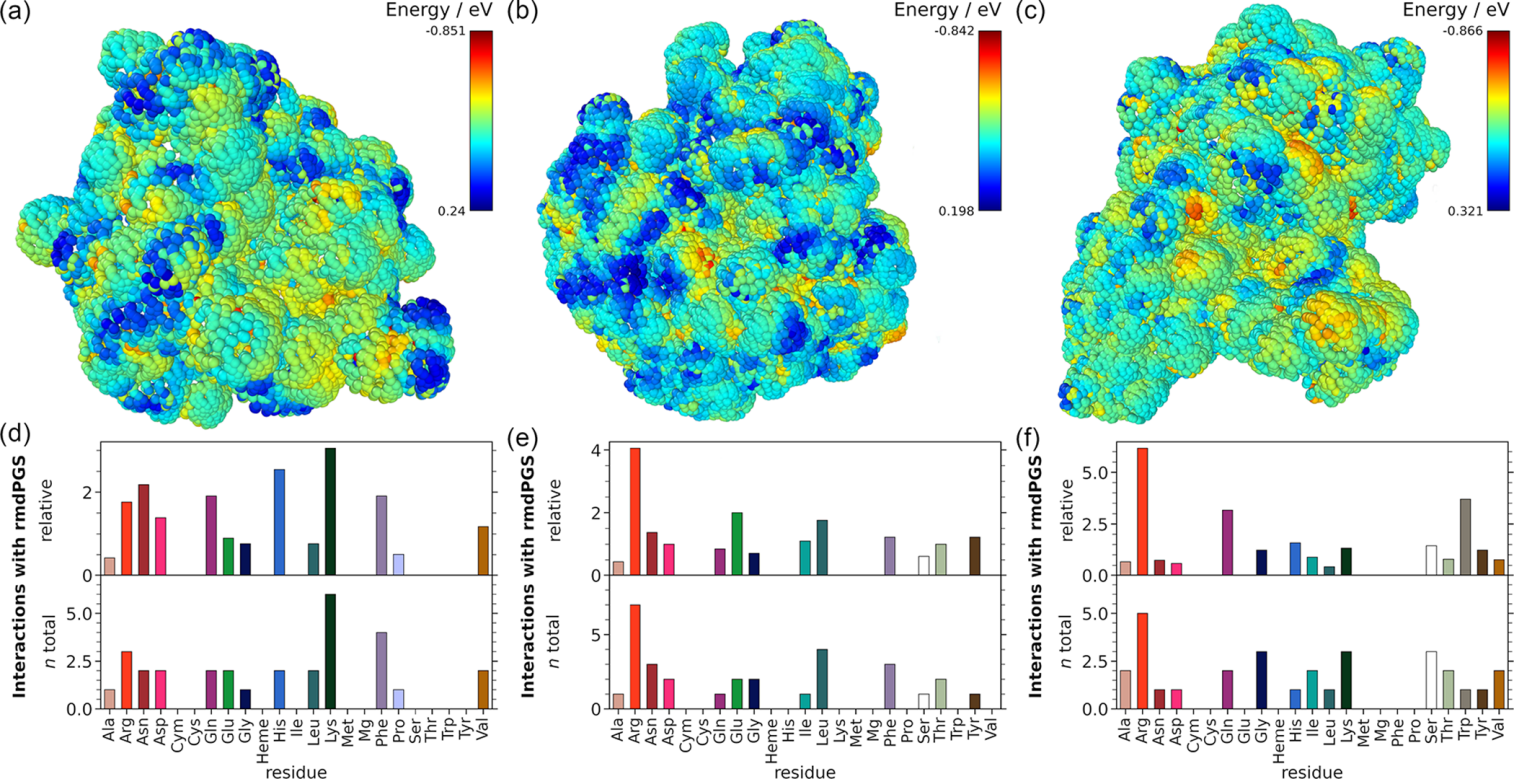
Comparison between the SASA interaction maps of OH with (a) *Cvi*UPO, (b) *Aae*UPO, and (c) GapA at 300 K. The bar plots underneath show the interactions per residue for *Cvi*UPO in (d), *Aae*UPO in (e), and GapA in (f). The color bar in the upper right corner of each interaction map indicates the value of the interaction energy for each species. More negative values are indicated in red, and less negative values in blue. The bar plots below show the relative and total interactions per amino acid. The heme cofactor (Heme), the coordinated Mg ion (Mg), and the cysteine group bound to the heme (Cym) are highlighted separately. The SASA procedure and analysis are consistent with the previous results.

Even though the three enzymes are differently shaped, the interactions with the *rmd*PGS follow similar trends. For all three enzymes, OH shows strong interactions with Arg. The main difference is the strong interaction of OH with Lys and Phe in *Cvi*UPO. In *Aae*UPO, OH has moderate interactions with Phe but not with Lys, whereas the opposite pattern is observed for GapA, where OH interacts to some extent with Lys but not with Phe. However, *Aae*UPO shows few strong interactions at Lys also with the other reactive species, such as O_2_ (Supplementary Figure S4e), likely because Lys occurs less frequently in *Aae*UPO than in *Cvi*UPO, which can be clearly seen in the relative data. The same argument applies to GapA, Phe is less abundant in this enzyme compared to *Cvi*UPO. While the interaction partners for H are almost identical for all three enzymes, H_2_O_2_ shows variation: in *Aae*UPO, it interacts most with Arg, Asp, and Trp, and in GapA, with Arg, Thr, and His, the latter interactions being particularly frequent. These differences likely stem from structural variations, such as the abundance of Thr-rich beta sheets in GapA. NO and O interact similarly across proteins, primarily with Glu (NO) and Arg/Lys (O), though NO interacts less with GapA, suggesting a possible lower affinity and better protection, but this would need to be confirmed experimentally. O_2_ binds mostly to Arg and Lys in *Cvi*UPO and GapA, while in *Aae*UPO, Arg, Leu, Phe, and Glu dominate. Overall, H and NO show the fewest surface interactions. Near the heme center, only two low-energy H_2_O_2_ interactions are found in *Aae*UPO (Glu196, Phe199). In GapA, H_2_O_2_, O_2_, OH, and O interact close to the reactive center, especially with Thr152 and Gly210, but no direct oxidation of active site cysteine Cys150 by H_2_O_2_ could be observed with SASA analysis. However, O_2_ engaged several times with a hydrogen atom (HN_2295_) from reactive Cys150.

The result emphasizes that the species important for catalysis, for example, H_2_O_2_ and O_2_, are pulled toward the active site in all three enzymes, but in comparison, the number of total interactions at the active site is lower than at other positions on the protein surface. This is partly due to the fact that there is only one reactive center in each of the enzymes studied, so the probability of interaction with this center is lower than with the more abundant residues of the enzyme. Moreover, the reactive center of the protein is less accessible than the amino acids on the protein surface.

With increasing temperature, the interactions of the *rmd*PGS with *Aae*UPO and GapA change in a similar way as for *Cvi*UPO. The residues with the most interactions change slightly because new interaction spots are available when the protein starts unfolding. Therefore, the change between 300 and 400 K is small, because the proteins are still quite stable at these temperatures. However, at around 500 K, the proteins lose their structure and shape, resulting in a more pronounced change in the interaction profile. Interestingly, *Cvi*UPO and GapA have the most positions with a favorable interaction energy in the active pocket at 400 K. At 500 K, the active pocket has fully collapsed due to the unfolding, and access pathways to the active center are blocked. *Aae*UPO, on the other hand, shows the most interactions within the active pocket at 500 K. The short validation MD simulations of the predicted positions in *Aae*UPO and GapA also showed similar results to *Cvi*UPO. In vacuum, mostly H, O, and OH instantly react with the enzymes at the predicted positions (compare Tables S3 and S4 in the SI). The hydrogen atoms bind to an unsaturated oxygen atom, while O and OH abduct hydrogen atoms from the protein. This behavior is independent of the tested temperature. In the solvent, we also observe identical behavior as for *Cvi*UPO. Most *rmd*PGS would rather interact with the solvent than with the proteins.

### Interactions of *Cvi*UPO with High *rmd*PGS Concentrations

Under experimental conditions, the *rmd*PGS will not diffuse toward the enzyme systematically but rather via random diffusion from the interface of the plasma (DBD) or the effluent (μAPPJ, CPJ) with the liquid through the solvent to the protein. To approach more realistic conditions, we performed MD simulations with a high concentration of *rmd*PGS in a random configuration around the enzyme. These simulations are intended to serve as an extended proof of concept for the SASA fast screening approach. The simulations were performed at different temperatures (300, 400, and 500 K) and repeated 3 times with different random orientations of the *rmd*PGS. The total simulation time for these simulations was 75 ps with a 0.15 fs time step. Additionally, they were conducted in a vacuum and in solvent. The results for the interactions of 100 OH atoms with *Cvi*UPO are displayed in Figure 5, and the other species can be found in the SI (Supplementary Figures S10–S14).

**Figure 5:**
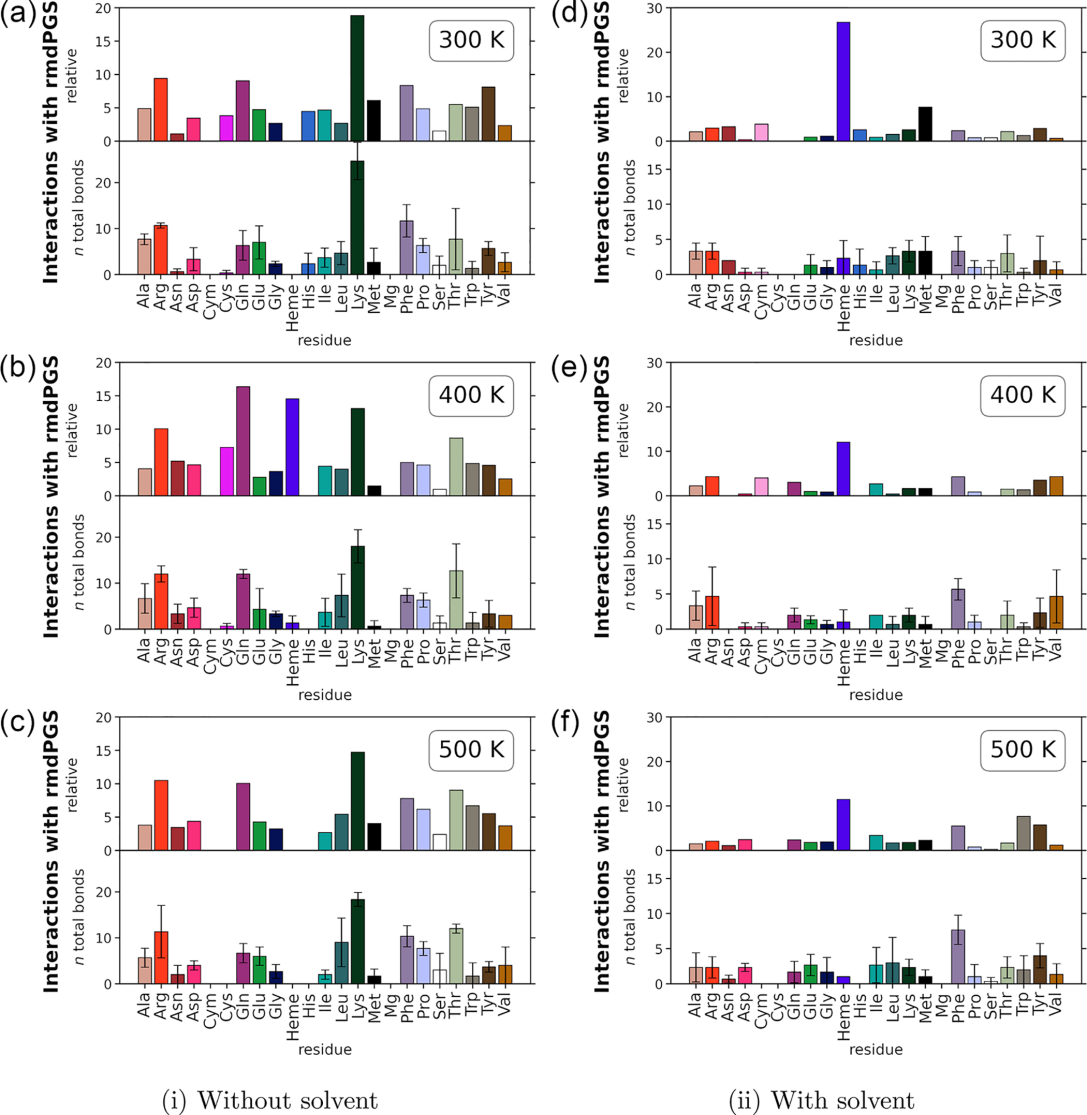
Bond analysis for high concentrations of OH with *Cvi*UPO. All panels show the total and the relative number of bonds to an additional OH molecule per residue. The bar plots below show the relative and total interactions per amino acid. The heme cofactor (Heme), the coordinated Mg ion (Mg), and the cysteine group bound to the heme (Cym) are highlighted separately. The relative interactions are calculated following eq 3. The panels (a) and (d) show the interactions at 300 K, without and with solvent, respectively. (b) and (e) Interactions at 400 K. (c) and (f) Interactions at 500 K. All simulations were conducted as described at the beginning of this section as well as in section Computational Details.

Figure 5a shows that in the absence of solvent, OH binds frequently to Lys, Phe, and Arg at 300 K, which aligns with the SASA predictions (see Figure 3). At higher temperatures, this trend continues, and as predicted by the SASA analysis, the interactions with Gln increase (Figure 5b,c). For the other *rmd*PGS, the SASA predictions also agree well with the MD simulation results. Glu and Lys remain the main partners for H (Figure S10), and the interactions of O closely follow the predictions (Figure S14). H_2_O_2_ has fewer interactions with Ser and Glu than expected, but more with Arg, especially at 500 K (Figure S11). Similar to the other *rmd*PGS, NO mainly interacts with Lys, although Asp and Glu are underrepresented. Interestingly, with rising temperature, this deviation decreases (Figure S12). The only outlier appears to be O_2_, with too few interactions in simulations (Figure S13). Overall, interaction trends and amino acid preferences remain consistent across temperatures, supporting the reliability of SASA predictions.

The trend from the simulations without solvent is also visible in the simulations with solvent (Figure 5d–f), despite the high error bars. A detailed analysis of the newly formed bonds involving the additional OH revealed that, in the solvent simulations, the majority react with the solvent rather than with the enzyme surface. This is in accordance with the short validation simulations from section SASA Interaction Analysis *Cvi*UPO. The surrounding water molecules protect the enzyme from being modified by the *rmd*PGS.

The solvent simulations with the other *rmd*PGS also follow the trend from the SASA predictions (compare Figures S10–S14). However, this observation should be taken with care, because of the relatively large error bars. The analysis of the high concentration simulations emphasizes that not only do properties of the protein surface play a role but also the initial position and the diffusion pathways of the *rmd*PGS toward the protein.

Some differences between the SASA predictions and the MD simulations are expected, as the SASA method works with predetermined points and considers only the most favorable interactions, while the MD simulations work with a fixed concentration of *rmd*PGS in a random distribution. However, the reliability of the SASA prediction is encouraging.

To achieve more reliable statistics, this type of simulation would need to be repeated more often under varying initial conditions. While this approach would improve statistical accuracy, it is also highly time-intensive. Given that the trends observed in these simulations align with the SASA results, the SASA method is certainly an interesting rapid approach for the fast screening of protein–plasma interactions.

The same behavior can also be observed with the enzymes *Aae*UPO and GapA, and a detailed description of their interaction can be found in the SI.

### Mass Spectrometry of Plasma-Treated *Cvi*UPO

In order to investigate the interactions of amino acids of the *Cvi*UPO with PGS, the r*Cvi*UPO was treated with the DBD for 5 min since most free proteins are typically inactivated after 5 min of treatment time and was then digested with trypsin for MS measurements. Besides Met and Cys, the amino acids Phe, Arg, and Lys were chosen for the modification analysis because of their predicted strong interaction with ROS, such as OH (SASA interaction analysis). Initially, the predicted reactions with ROS were investigated experimentally. The mass spectrometric analysis showed highly significant oxidation of Phe51, Phe67, Phe208 (Phe+O), Met42, and Met220 (Met+O) in all three replicates after 5 min of plasma treatment (Table 1).

**Table 1:**
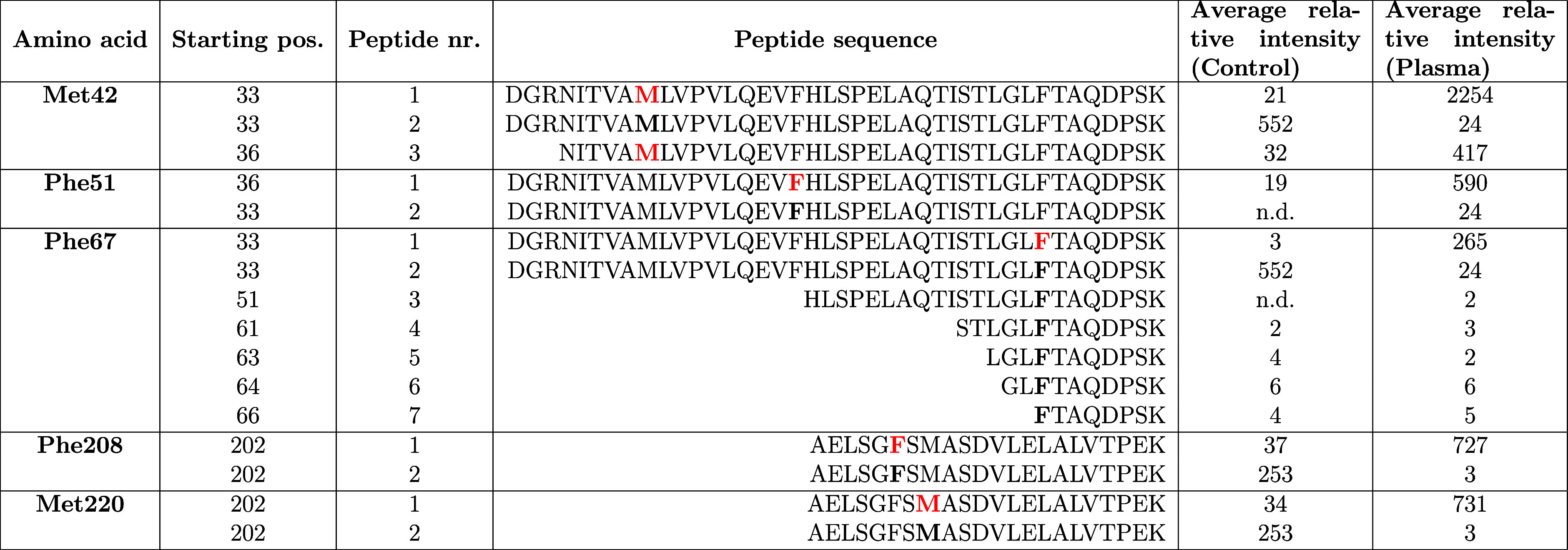
Amino Acid Residues of *Cvi*UPO Oxidized by Plasma Treatment^a^

a Protein samples were treated with plasma (Cinogy DBD) for 5 min and further processed for LC-MS/MS measurement. Five amino acid residues were found to be oxidized by plasma treatment. The average relative intensities of the peptides containing these five amino acids in unmodified form (bold, black) or oxidized (bold, red) in untreated controls and plasma-treated samples are shown, sorted by the starting position of the peptide (*N* = 3). Modifications were detected in all three replicate experiments with relative intensities >100. n.d.: not detected.

The strong interaction with PGS and Phe may be explained by the fact that many Phe residues in *Cvi*UPO are exposed to the outside of the protein. The aromatic ring of Phe seems to react with OH or other highly reactive oxygen species. Two of the modified phenylalanines, Phe51 and Phe67, are located near the substrate channel, and their modification could influence the enzyme activity. The chemical reactions of free amino acids with plasma-derived RONS have been extensively studied, and it has been shown that Phe is rapidly oxidized in the aromatic ring and hydroxylation or nitration could be detected after longer plasma treatment of 10 min.^58^ Although experimental investigations have confirmed specific modifications, including the oxidation of aromatic amino acids such as Phe in DBD plasma-treated glycinin,^59^ plasma-induced amino acid modifications in proteins have not been extensively studied to date. Hence, SASA-based interaction predictions are highly relevant, as the observed modifications at Phe were significant and in agreement with our experimental data.

Since the active site of an enzyme is responsible for substrate and cosubstrate binding and thus also for enzymatic activity, the investigation after plasma treatment is of particular importance. The amino acids His90, Thr158, Phe88, Glu162, Tyr166, and Cys19 are primarily involved in the catalytic cycle.^56^ The residues His90, Glu162, and Tyr166 are conserved and located distal to the catalytic center, while the proximal Cys19 is involved in the reaction. Residues Phe88 and Thr158 are also involved in substrate–heme interactions close to the heme access channel. Although interactions with amino acids in the active center of the *Cvi*UPO like Phe88 were not observed in the experimental study, this could occur with longer plasma treatment times.

While Phe can undergo hydroxylation reactions, sulfur-containing amino acid residues in proteins like Cys or Met exhibit greater susceptibility to oxidative modifications and rapidly react with ROS.^60^ The thiol group of cysteine readily reacts with ROS, leading to the formation of disulfide bonds and sulfenic (−SOH), sulfinic (−SO_2_H), or sulfonic (−SO_3_H) acids, significantly altering protein structure and function. Changes in Cys residues, particularly those engaging in disulfide bonds or those participating in the catalytic reaction or cofactor binding, often lead to a loss of stability and protein unfolding and thus a total loss of function, that is, enzyme activity.^60,61^ In addition, amino acid modifications can also lead to degradation of the protein due to cleavage of the peptide bonds.^62^ Methionine, with its thioether group, is also a prime target for oxidation, resulting in the formation of methionine sulfoxide and, under prolonged oxidative stress, methionine sulfone.^58^ MS analysis showed modified Met42 and Met220 in all replicates, resulting in the majority of Met being oxidized after 5 min treatment (Met+O). However, interaction profiles from SASA showed no interactions between *rmd*PGS and Met or Cys, which might be explained by the fact that these are buried in the protein structure and not accessible on the solvent accessible surface, or that they are already engaged in covalent bonds (Cys19-Heme).

SASA interaction profiles predicted strong interactions between *rmd*PGS such as OH, H_2_O_2_, and O_2_ and the basic amino acids Lys and Arg, which are both known to be proton donor residues. However, amino acid modifications such as dehydrogenation of arginine, oxidation, and/or dehyrogenation of lysine were detected only in some replicates and at low relative intensity. Due to the positive charge, these amino acids could be modified by reactive nitrogen species (i.e., nitration reactions), which has not yet been investigated experimentally. For more detailed analyses, further possible modifications and plasma treatment times should be included. In addition, modifications to other amino acids should be investigated, and the sequence coverage should be increased from 70% in the current analysis. If amino acids in the active center of the enzyme are modified, this could lead to enzyme inactivation. Depending on the residues affected, enzyme engineering could be a viable approach to selectively exchange amino acids, thereby improving protein plasma stability.

## Conclusions

In conclusion, the SASA analysis method proves to be a valuable tool for identifying potential interaction sites on protein surfaces for small reactive species. In the scope of this study, the interactions of various plasma-generated species and three enzymes, *Cvi*UPO, *Aae*UPO, and GapA, were analyzed in detail, predicting potential modifications primarily on Lys, Arg, and Phe residues. A subsequent validation of any predicted interactions is advised because a high interaction energy does not necessarily translate into covalent binding at the respective position. Extended MD simulations were performed to evaluate the SASA analysis results. They highlighted that the presence of the solvent plays an important role. These calculations indicated that the reactive species react more frequently with water molecules than with the protein surface. This finding aligns well with the experimental results of Yayci et al.^63^ who found that an experimental setup that prolongs the diffusion path of reactive species in the solvent leads to a reduction in modifications of the enzymes. In addition, the longer MD simulations confirmed that even in a random configuration with enough time, some of the reactive species will bind to the enzymes on similar residues as predicted by the SASA analysis method, thereby supporting the reliability of the SASA analysis. Notably, the comparison with mass spectrometry data from plasma-treated *Cvi*UPO validated Phe modifications that were predicted by the SASA analysis, suggesting that the PGS ·OH and ·O play an important role in the *Cvi*UPO inactivation. While the Phe modification brought into focus by the SASA analysis was verified experimentally, modifications of Arg and Lys could not be confirmed by the experiment. This may be due to insufficient plasma treatment times to induce permanent modifications. Hydrogen abstractions may, e.g., have occurred, but the missing hydrogen could have been replaced by a solvent-derived hydrogen, effectively reversing the modification. Although Cys and Met modifications were predicted rarely by the SASA analysis, they were searched for in the MS analysis because these modifications are known to occur in plasma-treated proteins.^19,20,22^ The difference between our results and those of other studies in this field can be explained by the limited solvent accessibility of Met and Cys in *Cvi*UPO. However, Met modifications have been experimentally demonstrated, but they may have occurred when the enzyme was unfolded, and this residue was exposed.

Overall, combining SASA predictions with MS analysis provides valuable knowledge of plasma-induced enzyme modifications and highlights the potential of the SASA method to inform MS data interpretation. In the future, further experimental studies should be carried out to test the general validity of the SASA model by applying it to other systems. In addition, this study provides initial insights into which residue could be replaced in order to engineer a more plasma-stable biocatalyst.

## Supplementary Material





## Notes

The amino acid sequence of *Cvi*UPO (ZIP)
Detailed results of the SASA interaction analysis and the interaction analysis with high*rmd*PGS concentrations for all tested enzymes (PDF)

## Abbreviations

MD: molecular dynamics
RMD: reactive molecular dynamics
UPO: unspecific peroxygenase
PGS: plasma-generated species
rmdPGS: reactive molecular dynamics plasma-generated species
CPJ: capillary plasma jet
μAPPJ: mcroscale atmospheric pressure plasma jet
DBD: dielectric barrier discharge
SASA: solvent accessible surface analysis
N: number of particles, P: volume, T: absolute temperature: NVT ensemble
N: number of particles, P: pressure, T: absolute temperature: NPT ensemble
