# Supplementary figures and images for: Phenylalanine Modification in Plasma-Driven Biocatalysis Revealed by Solvent Accessibility and Reactive Dynamics in Combination with Protein Mass Spectrometry
Source: J Phys Chem B. 2025 Oct 23;129(44):11374–86. doi: 10.1021/acs.jpcb.5c03518 (PMC12598871; doi:10.1021/acs.jpcb.5c03518)

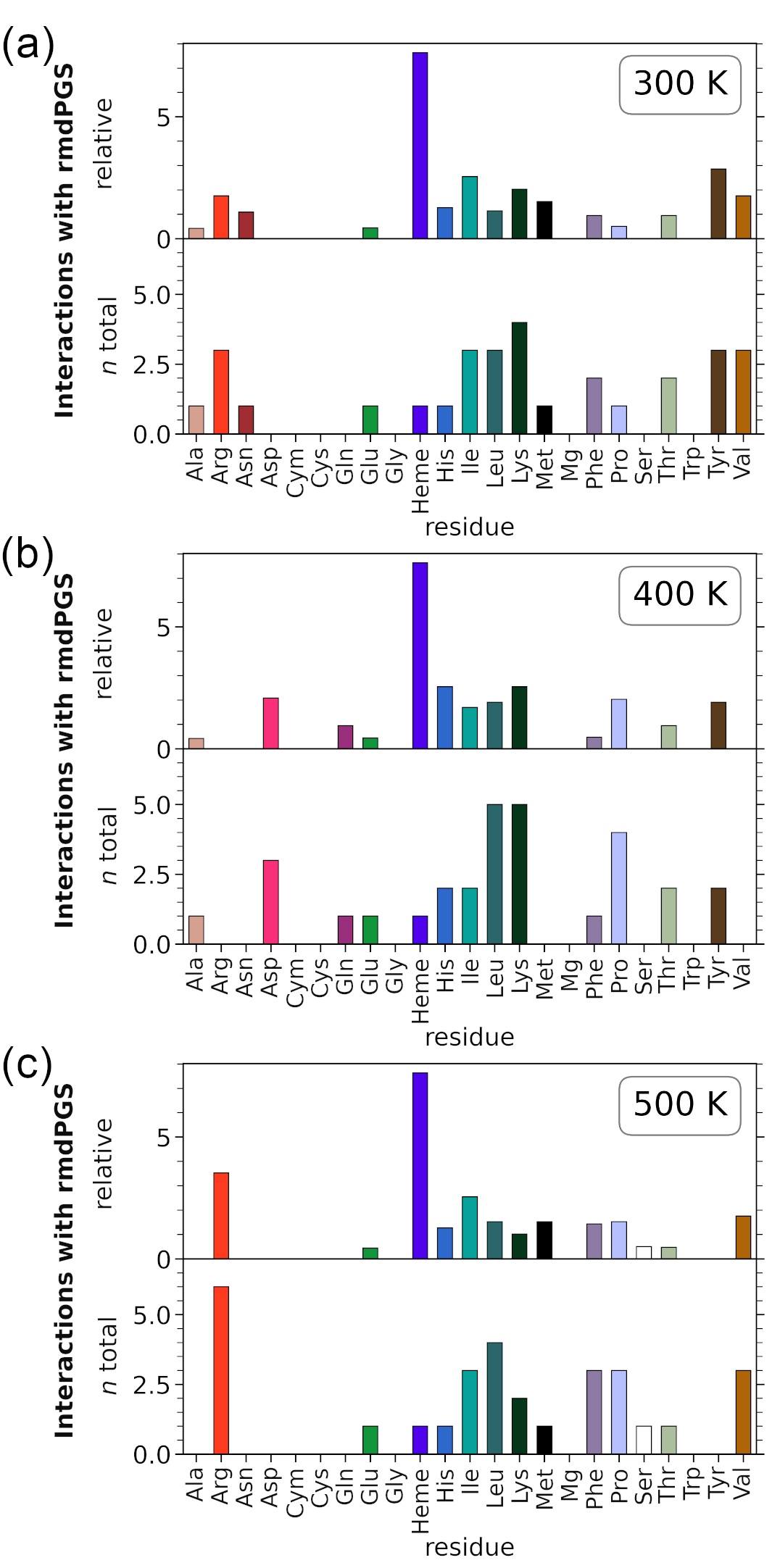

Supplement: Supplementary file 1 [file jp5c03518_si_001.zip › SI-Images/SASA_temps_Cvi_o2.png]

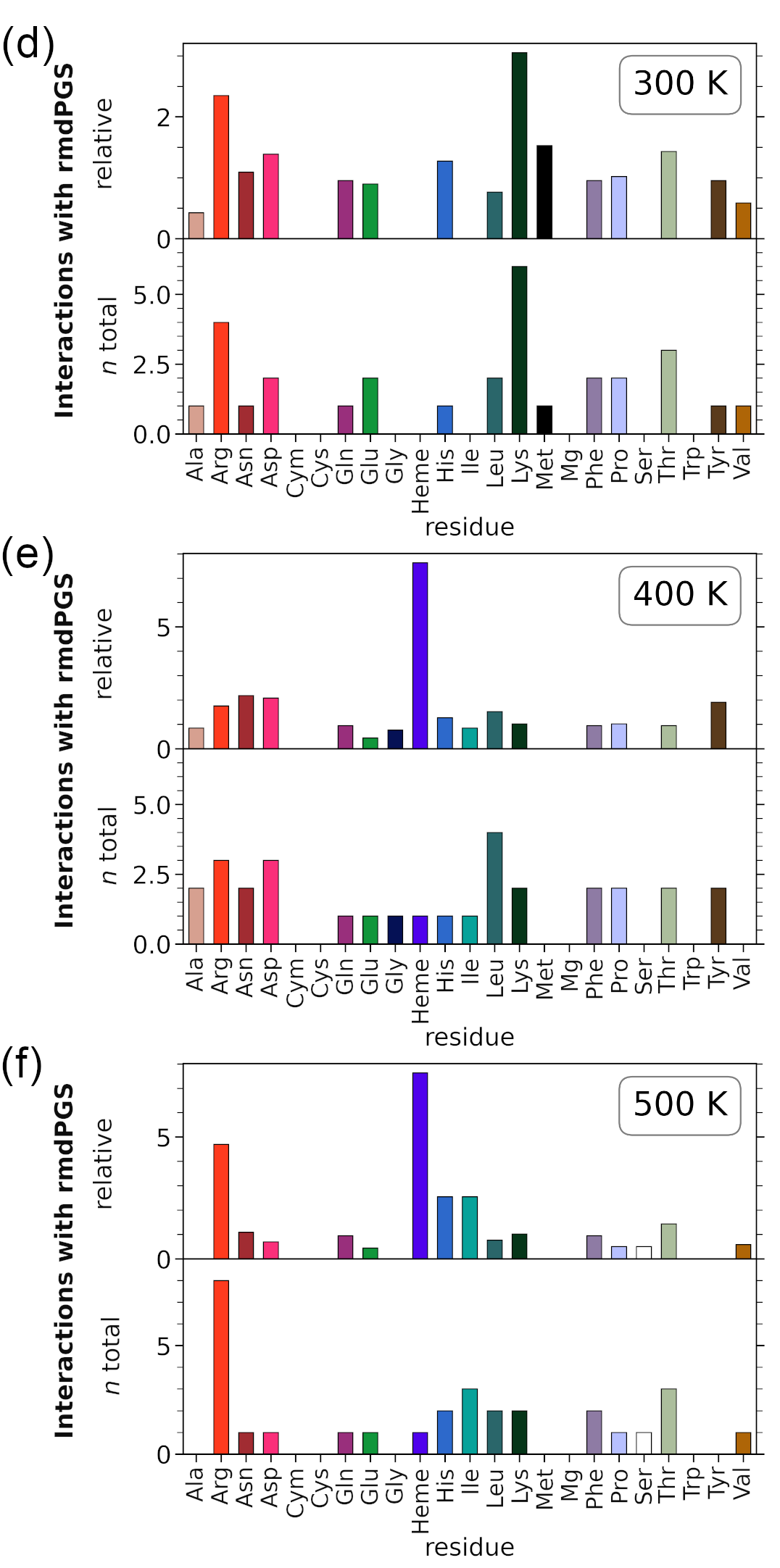

Supplement: Supplementary file 1 [file jp5c03518_si_001.zip › SI-Images/SASA_temps_Cvi_o.png]

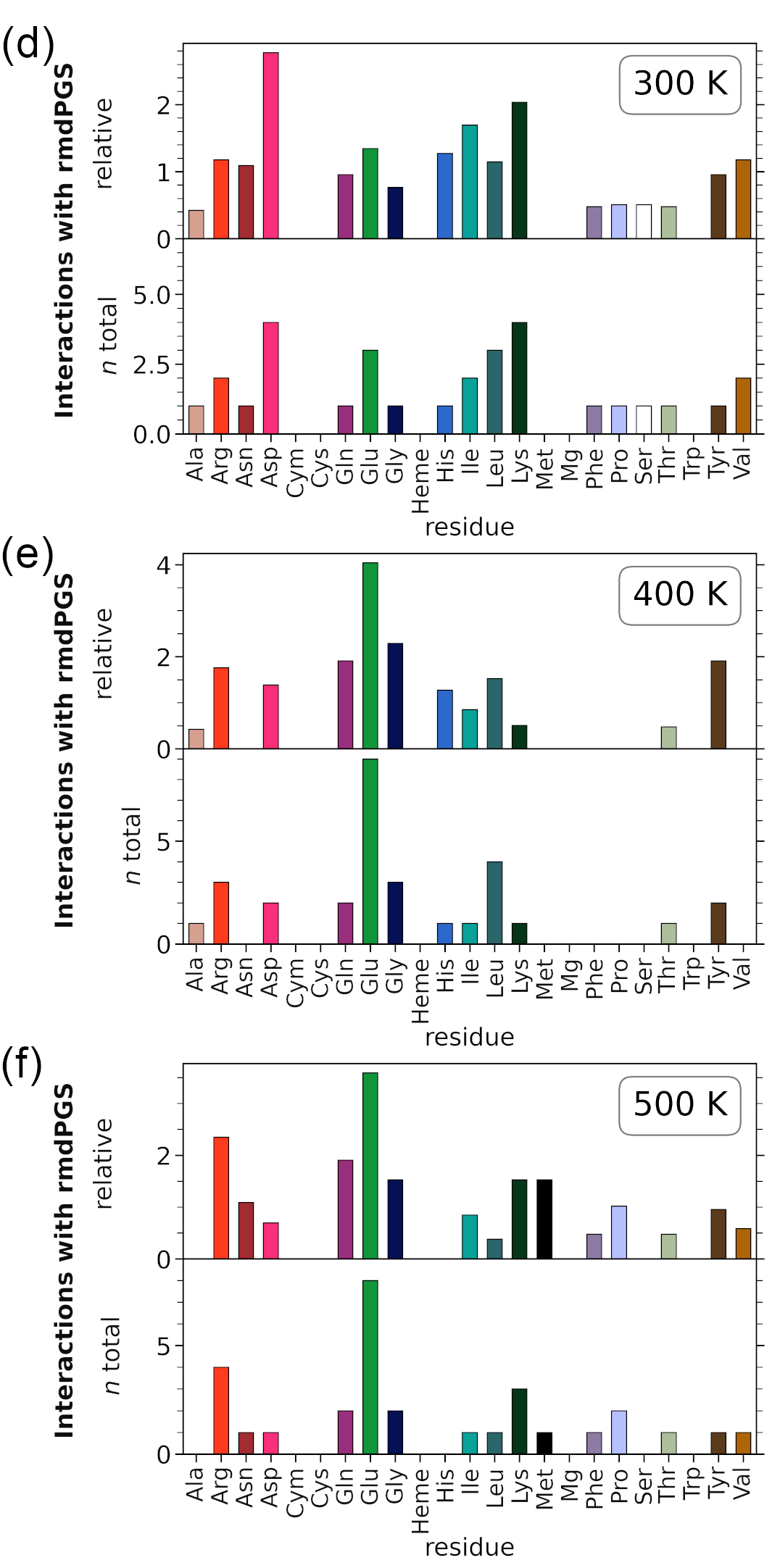

Supplement: Supplementary file 1 [file jp5c03518_si_001.zip › SI-Images/SASA_temps_Cvi_no.png]

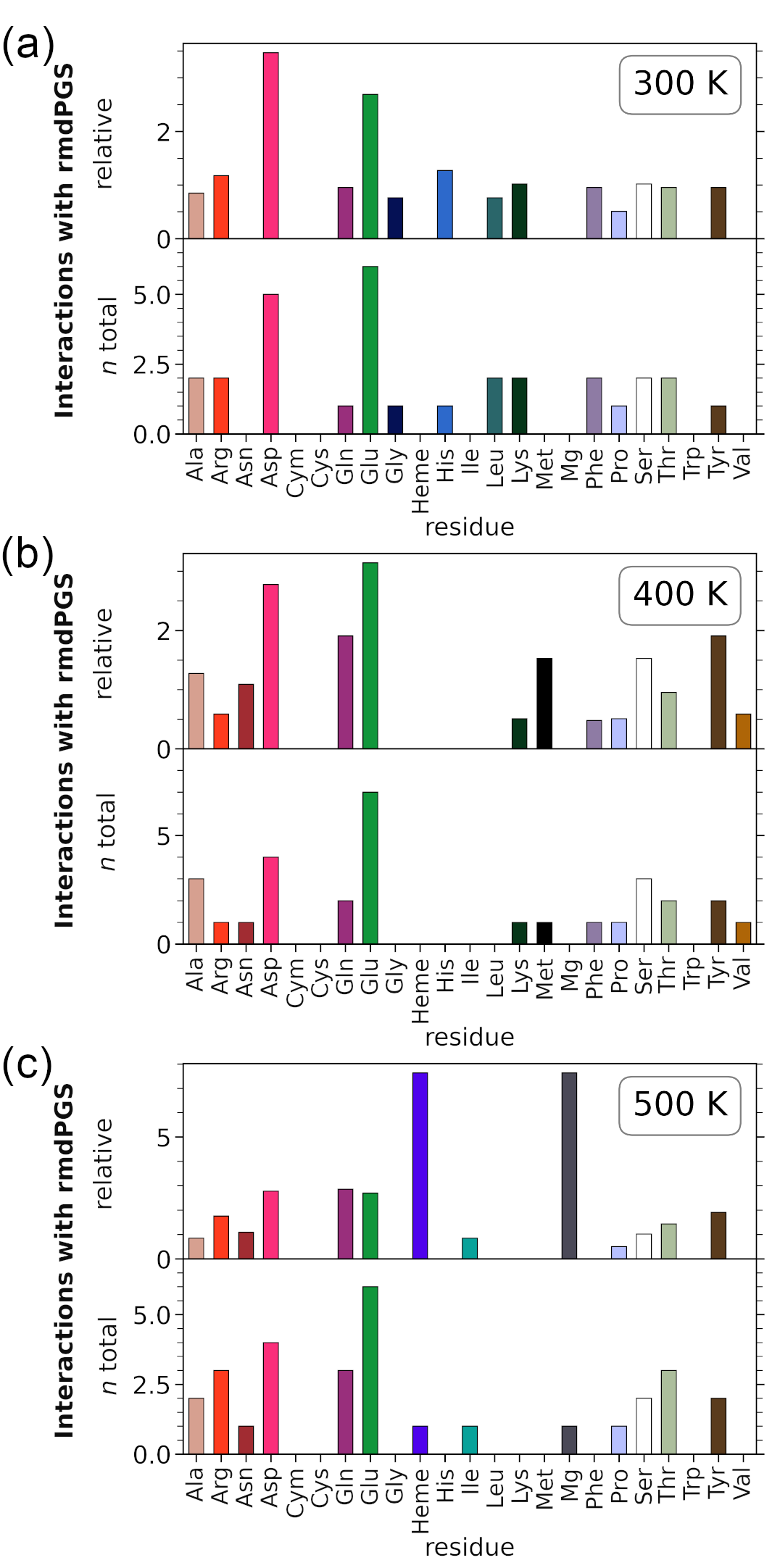

Supplement: Supplementary file 1 [file jp5c03518_si_001.zip › SI-Images/SASA_temps_Cvi_h.png]

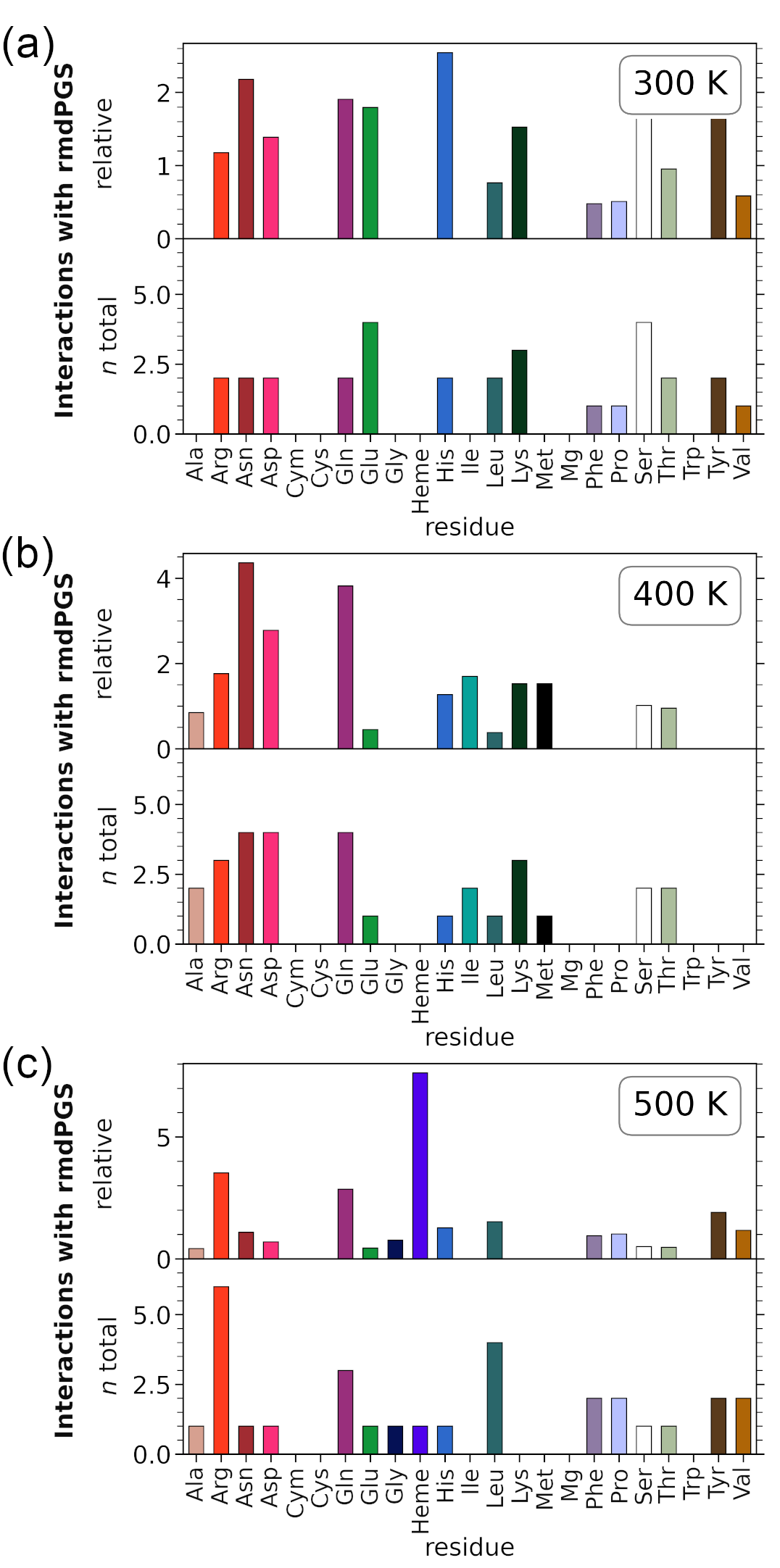

Supplement: Supplementary file 1 [file jp5c03518_si_001.zip › SI-Images/SASA_temps_Cvi_h2o2.png]

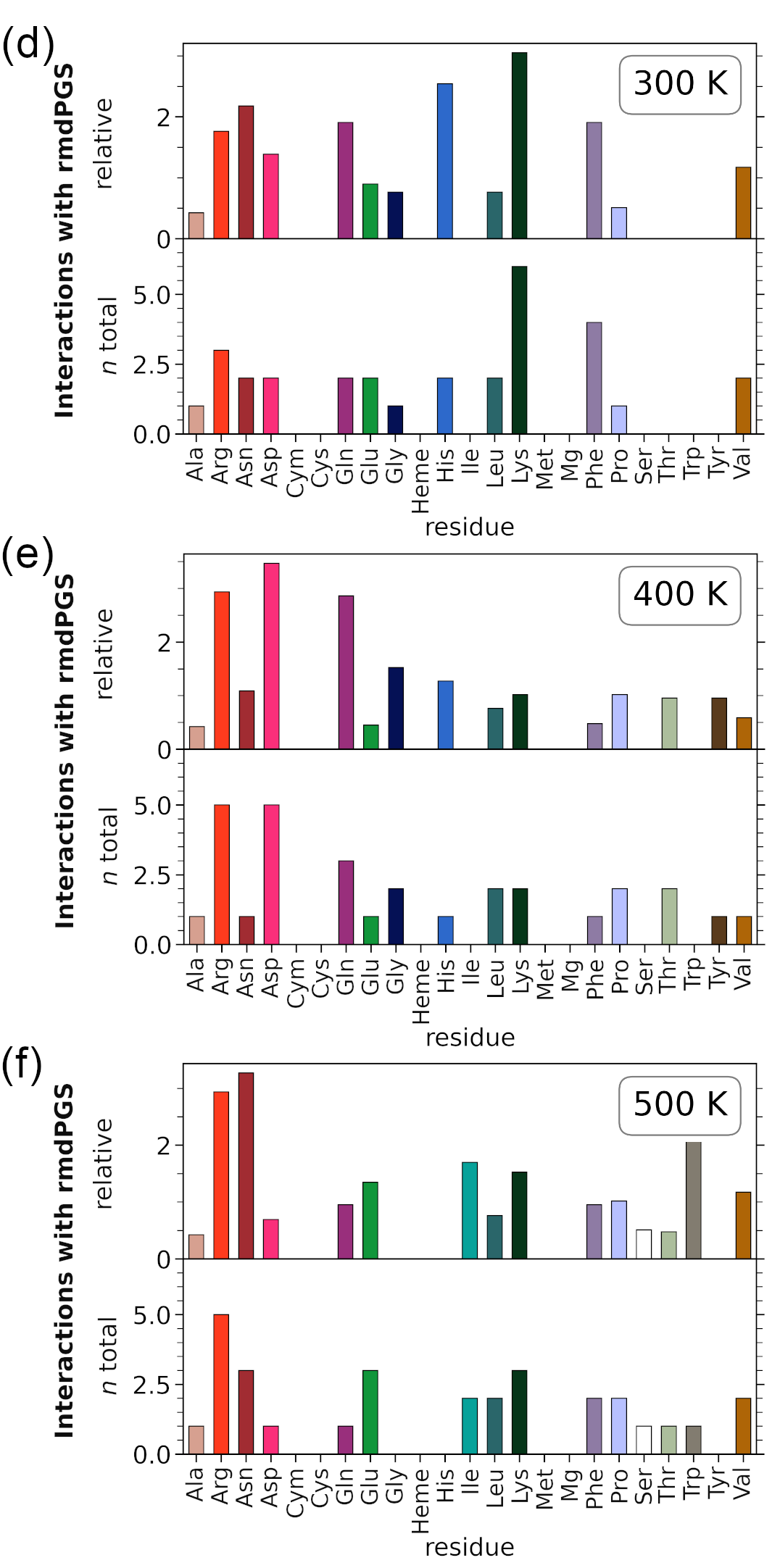

Supplement: Supplementary file 1 [file jp5c03518_si_001.zip › SI-Images/SASA_temps_Cvi_oh.png]

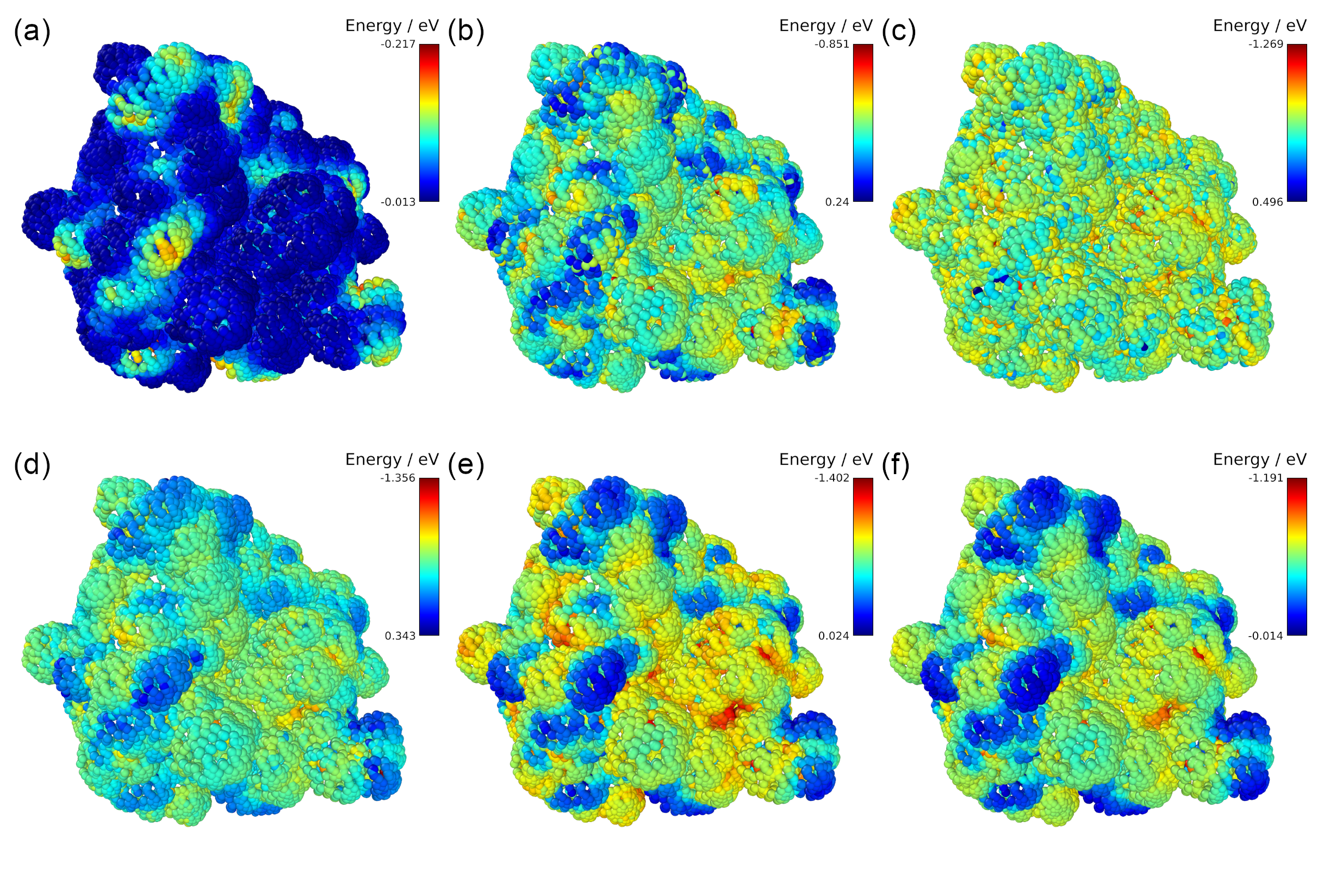

Supplement: Supplementary file 1 [file jp5c03518_si_001.zip › SI-Images/SASA_maps_overview_Cvi.png]

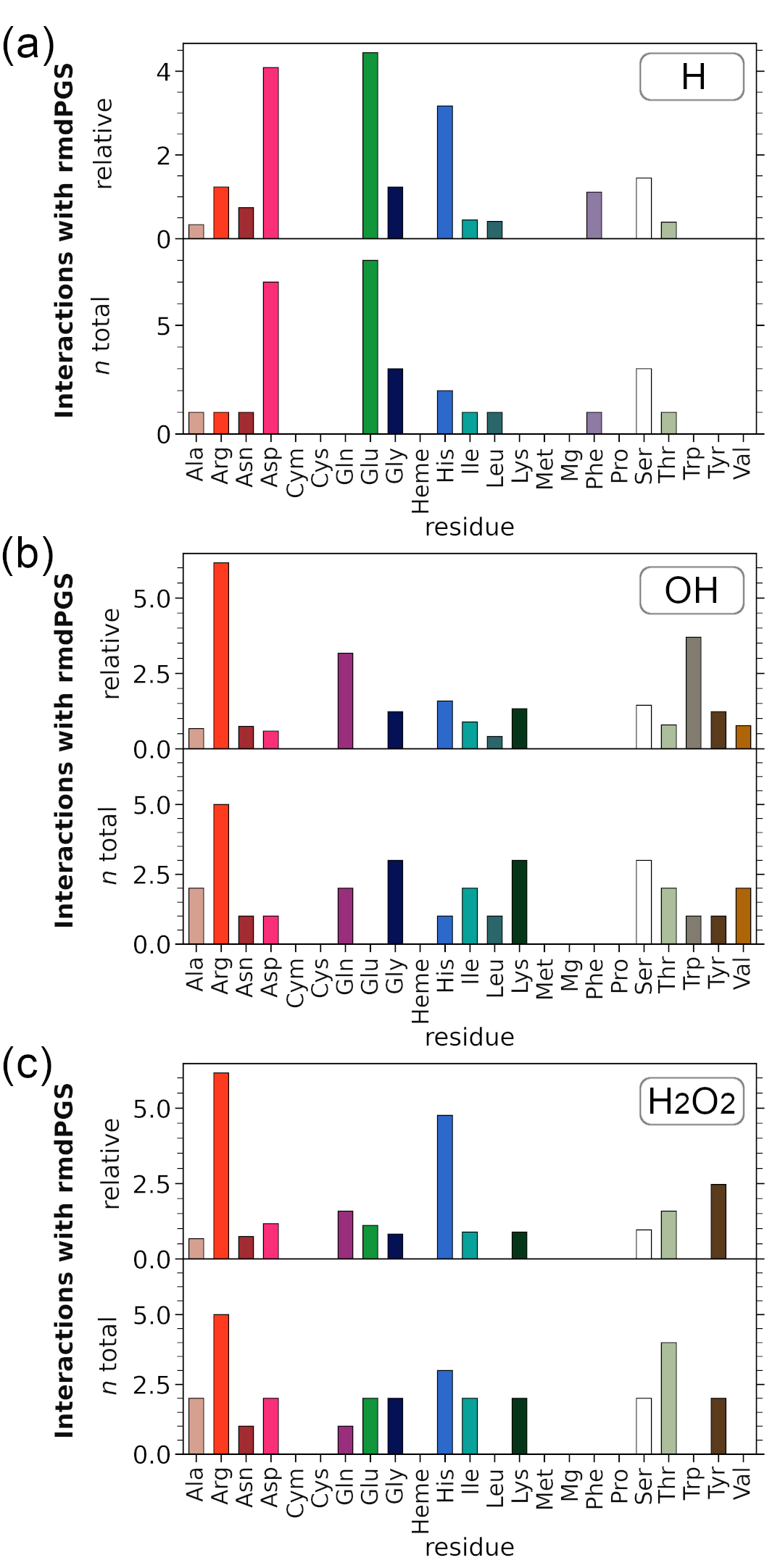

Supplement: Supplementary file 1 [file jp5c03518_si_001.zip › SI-Images/SASA_GapA_h_h2o2_oh.png]

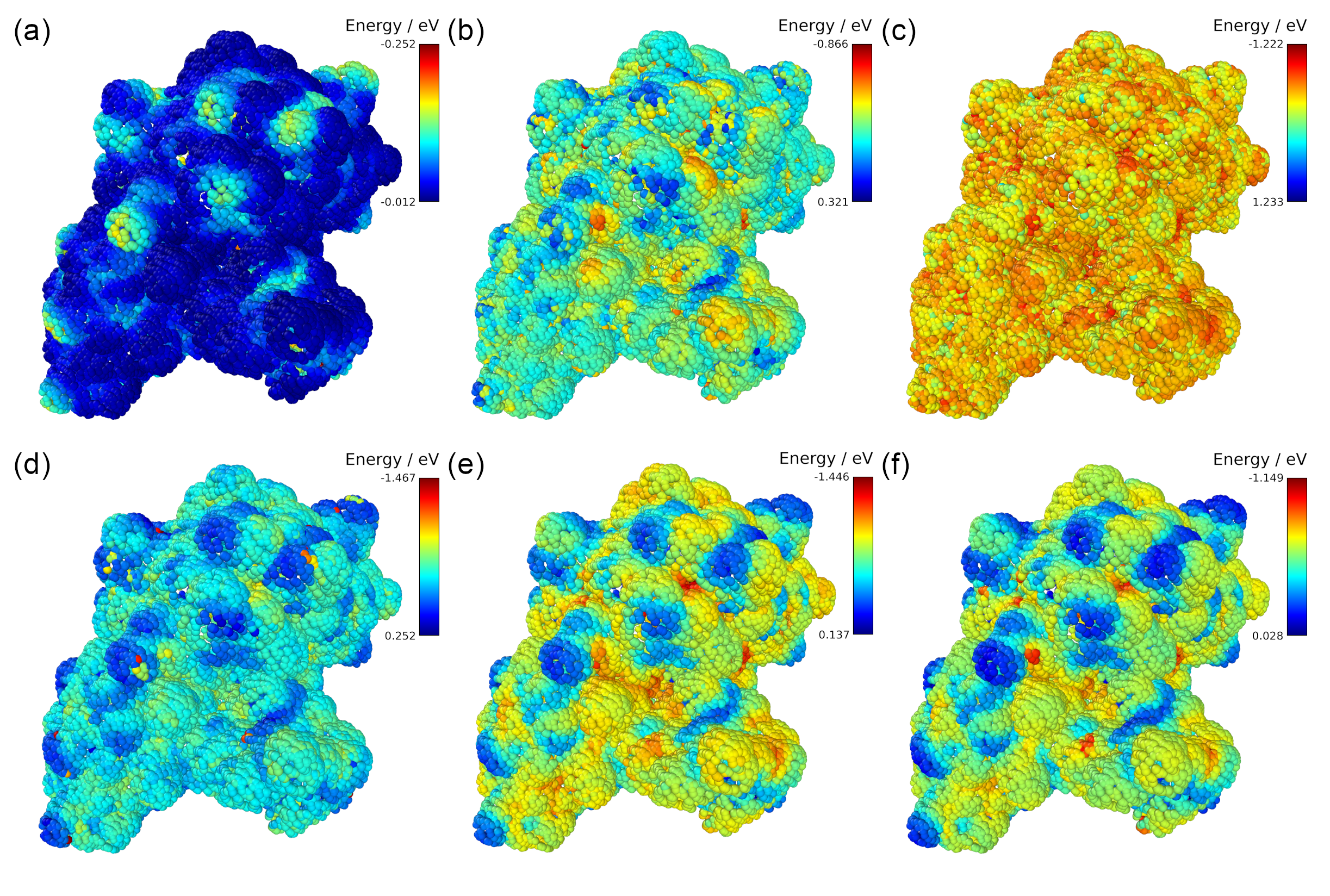

Supplement: Supplementary file 1 [file jp5c03518_si_001.zip › SI-Images/SASA_maps_overview_GapA.png]

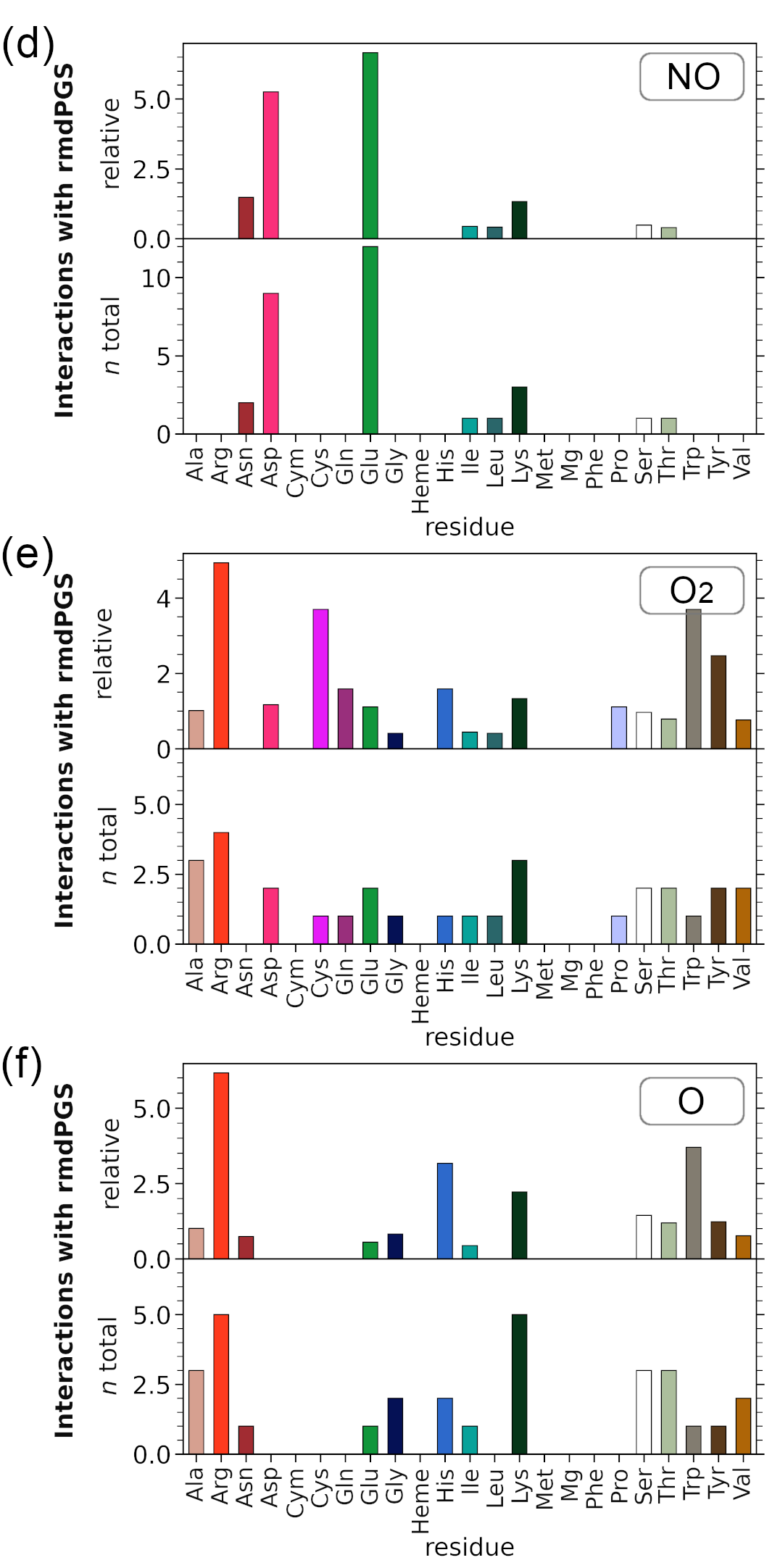

Supplement: Supplementary file 1 [file jp5c03518_si_001.zip › SI-Images/SASA_GapA_no_o2_o.png]

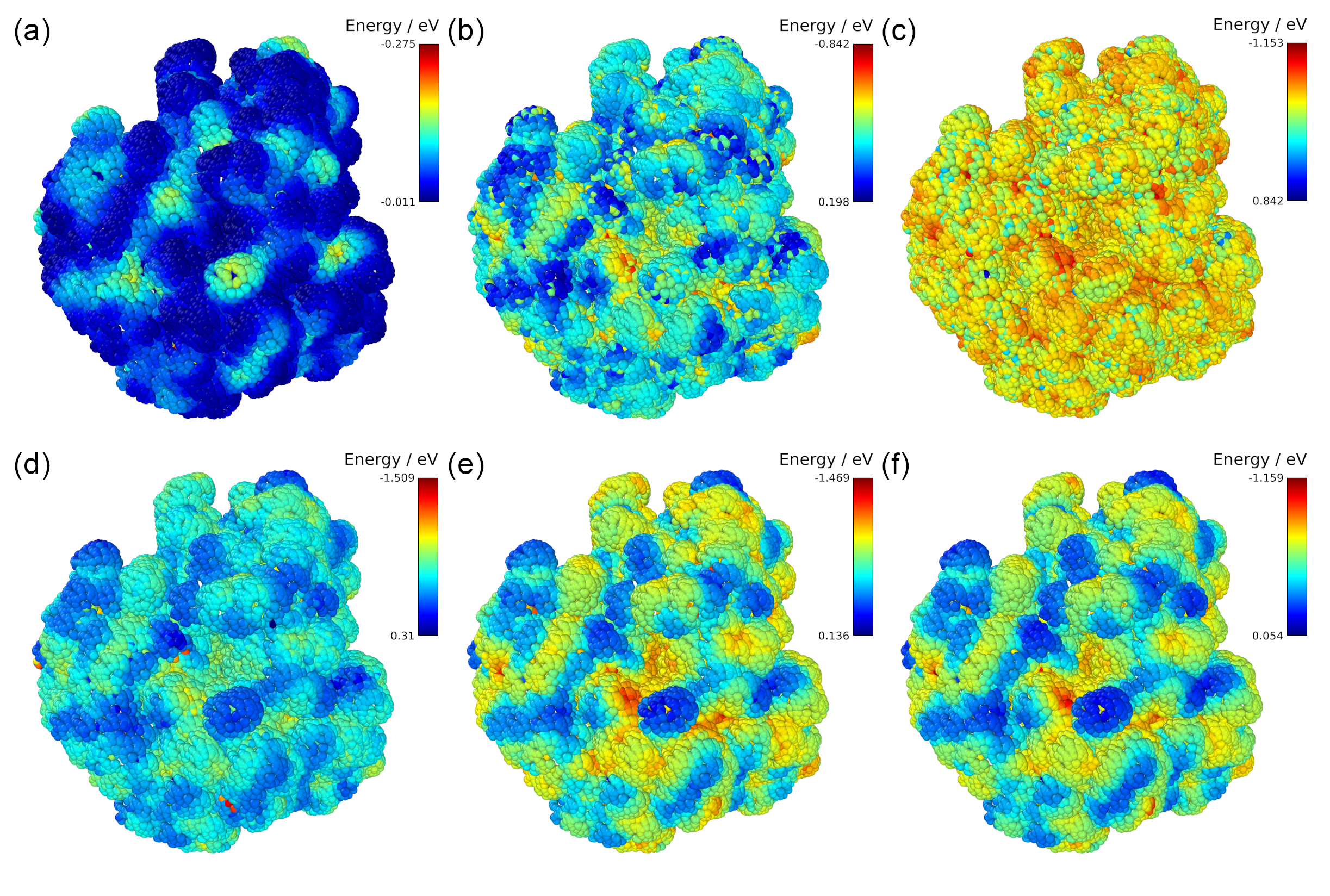

Supplement: Supplementary file 1 [file jp5c03518_si_001.zip › SI-Images/SASA_maps_overview_Aae.png]

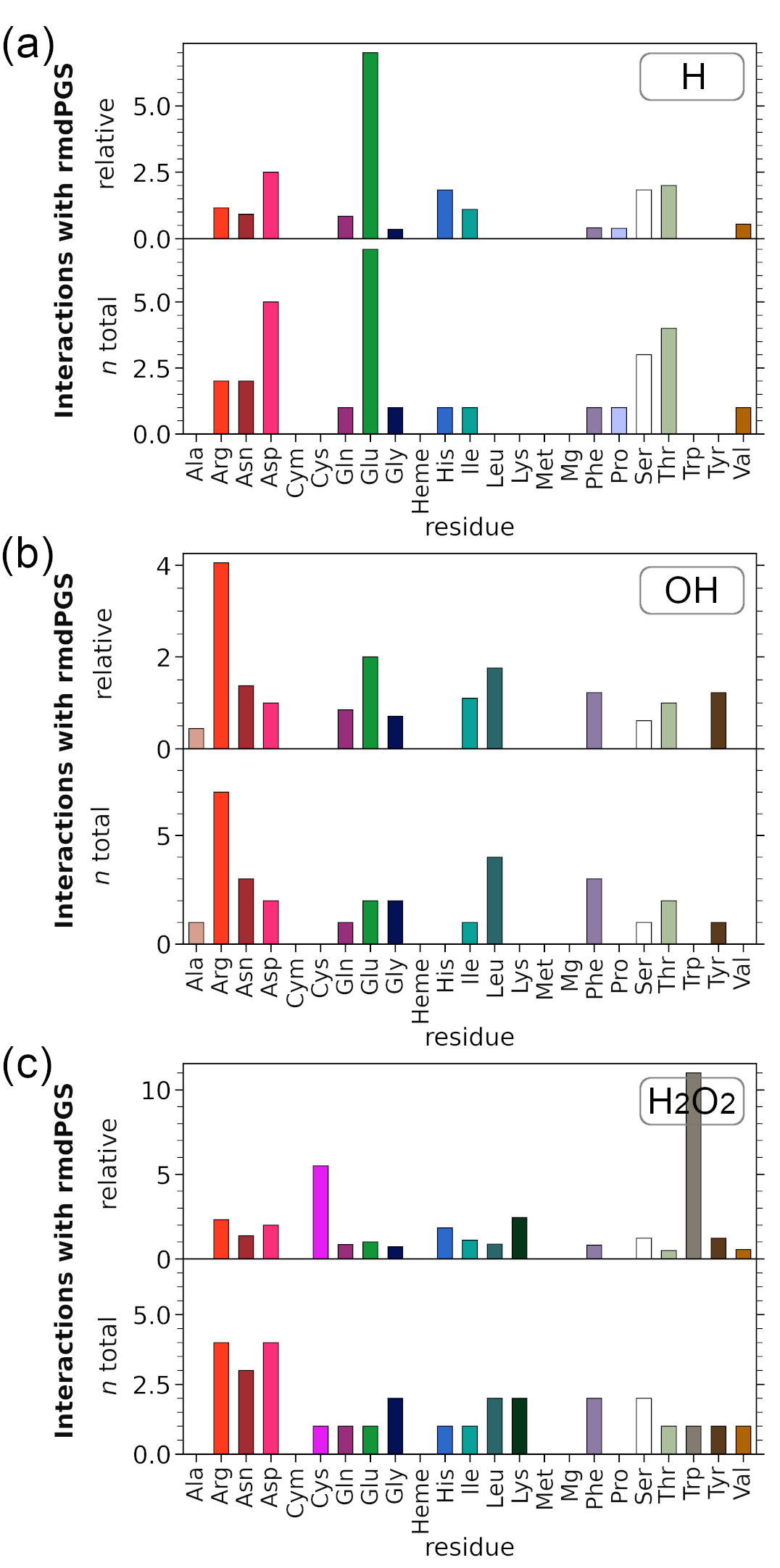

Supplement: Supplementary file 1 [file jp5c03518_si_001.zip › SI-Images/SASA_Aae_h_h2o2_oh.png]

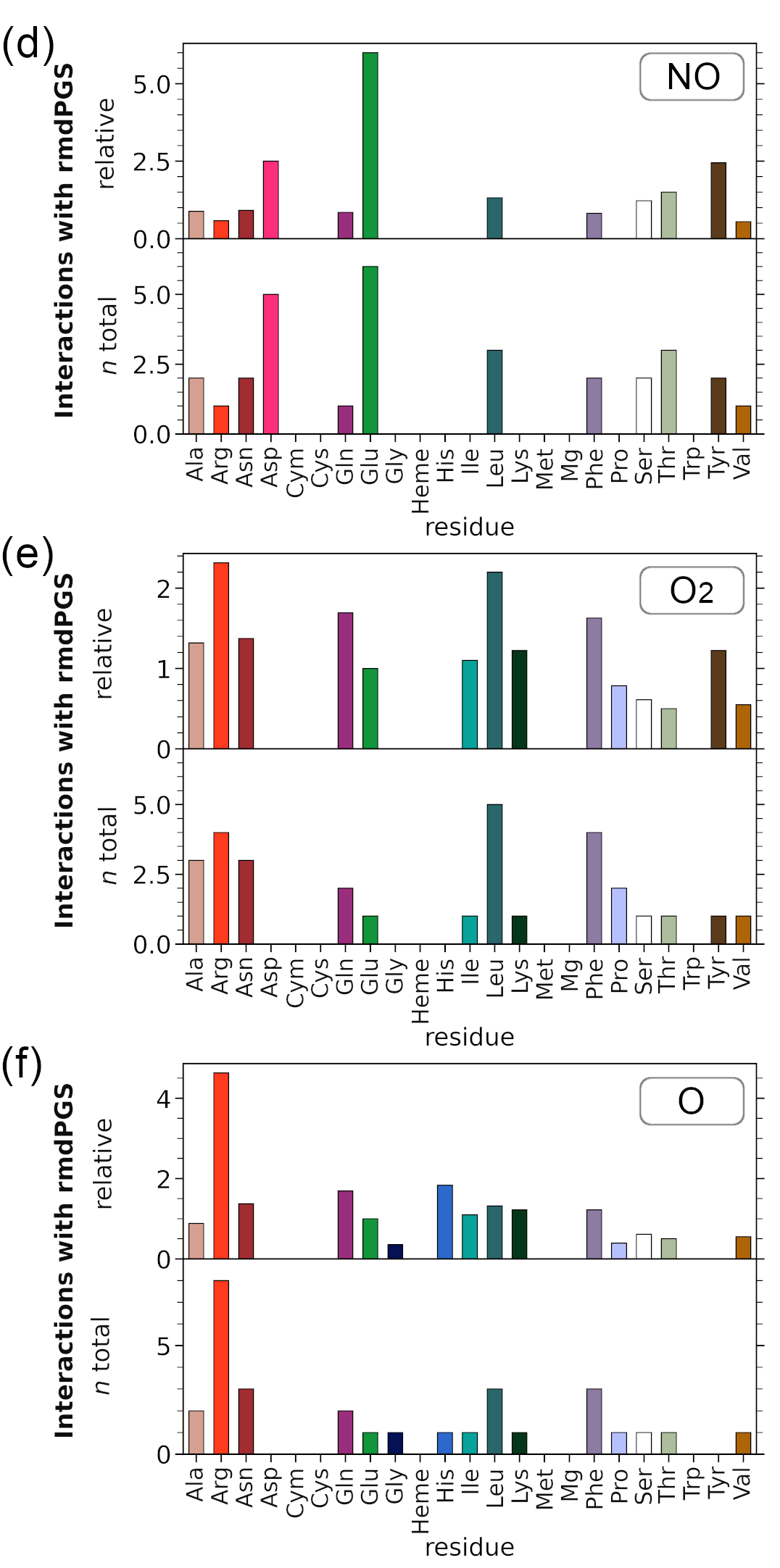

Supplement: Supplementary file 1 [file jp5c03518_si_001.zip › SI-Images/SASA_Aae_no_o2_o.png]

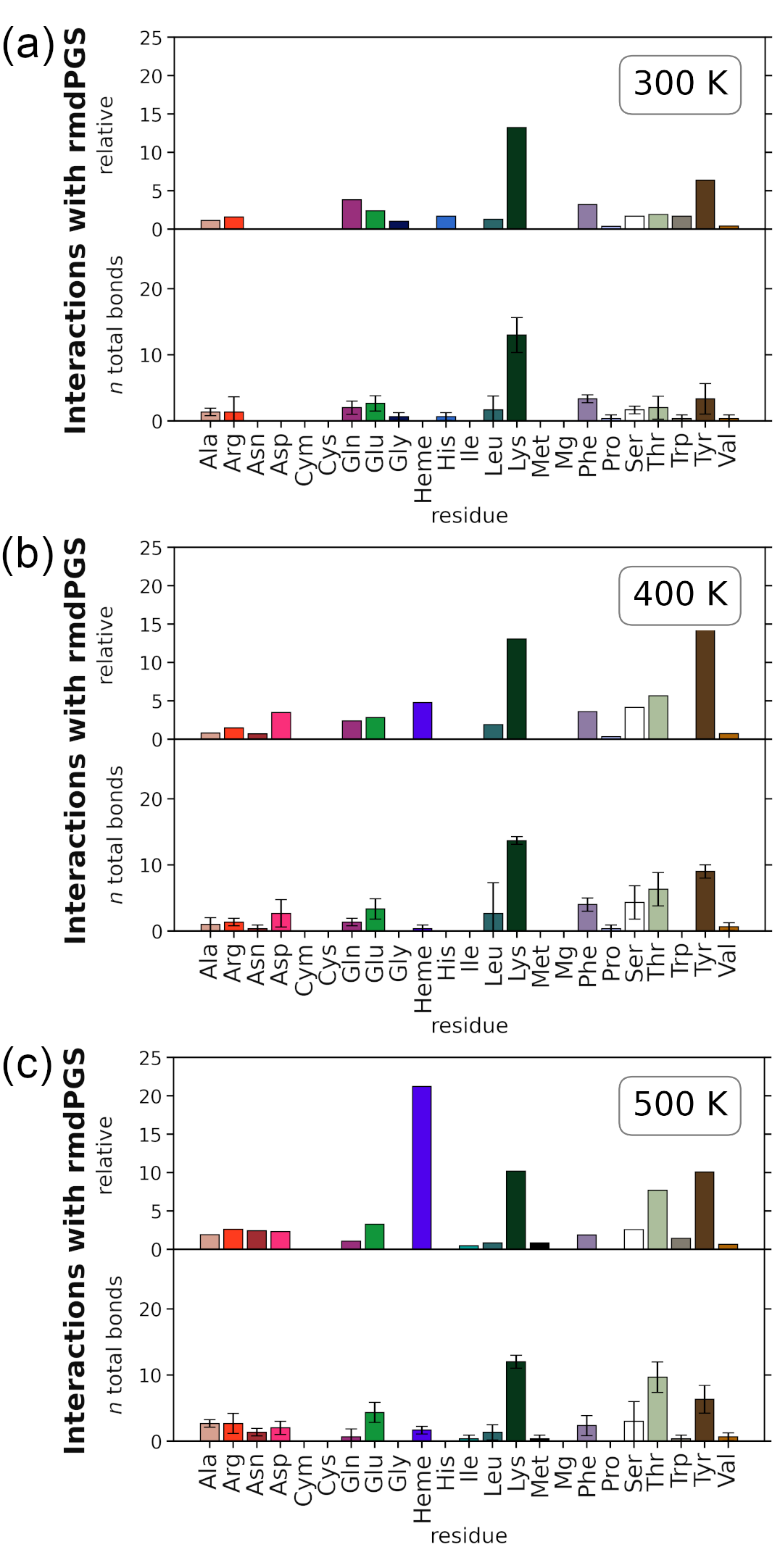

Supplement: Supplementary file 1 [file jp5c03518_si_001.zip › SI-Images/Conc_MD_Cvi_no_vac.png]

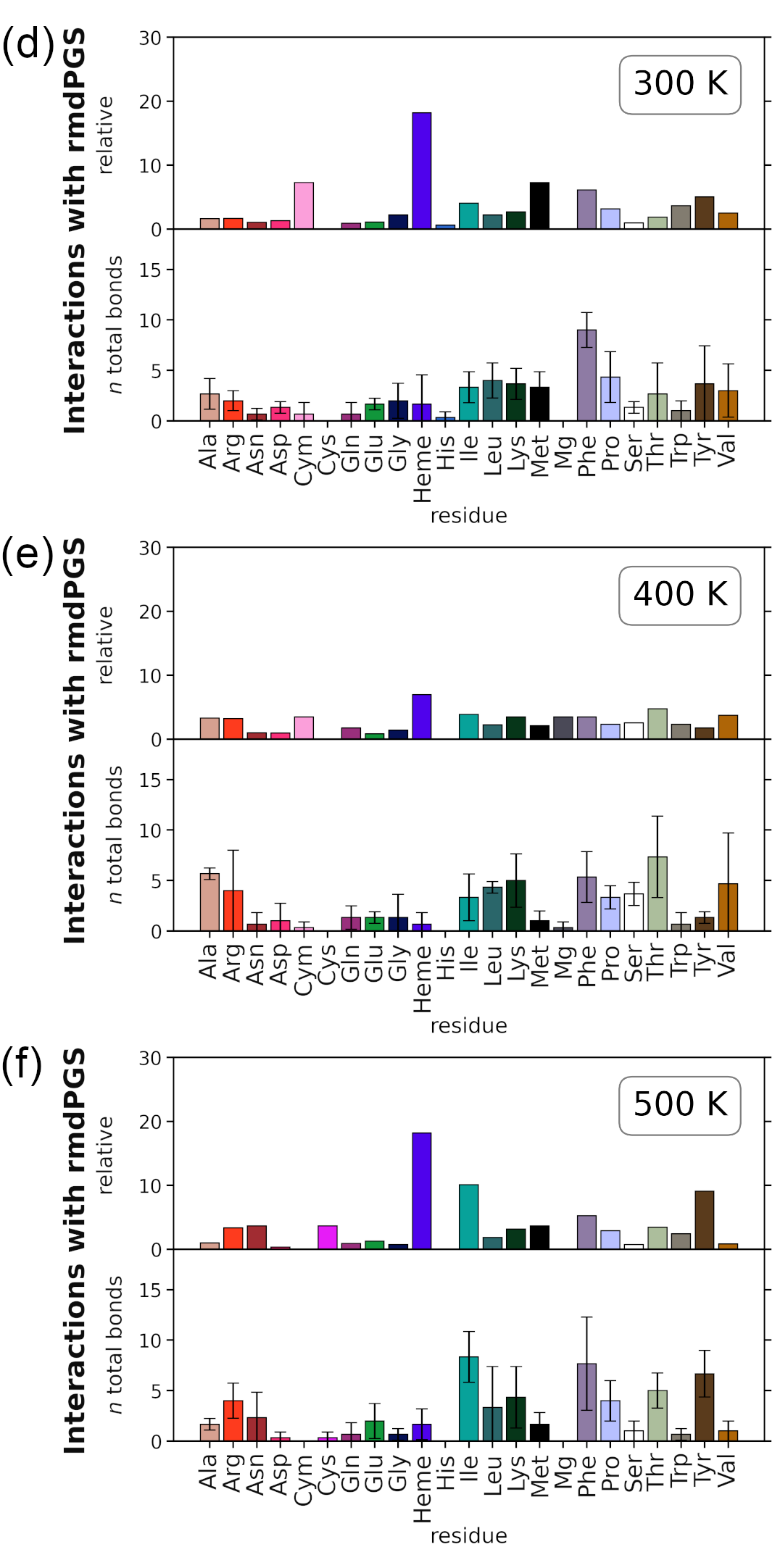

Supplement: Supplementary file 1 [file jp5c03518_si_001.zip › SI-Images/Conc_MD_Cvi_o_solv.png]

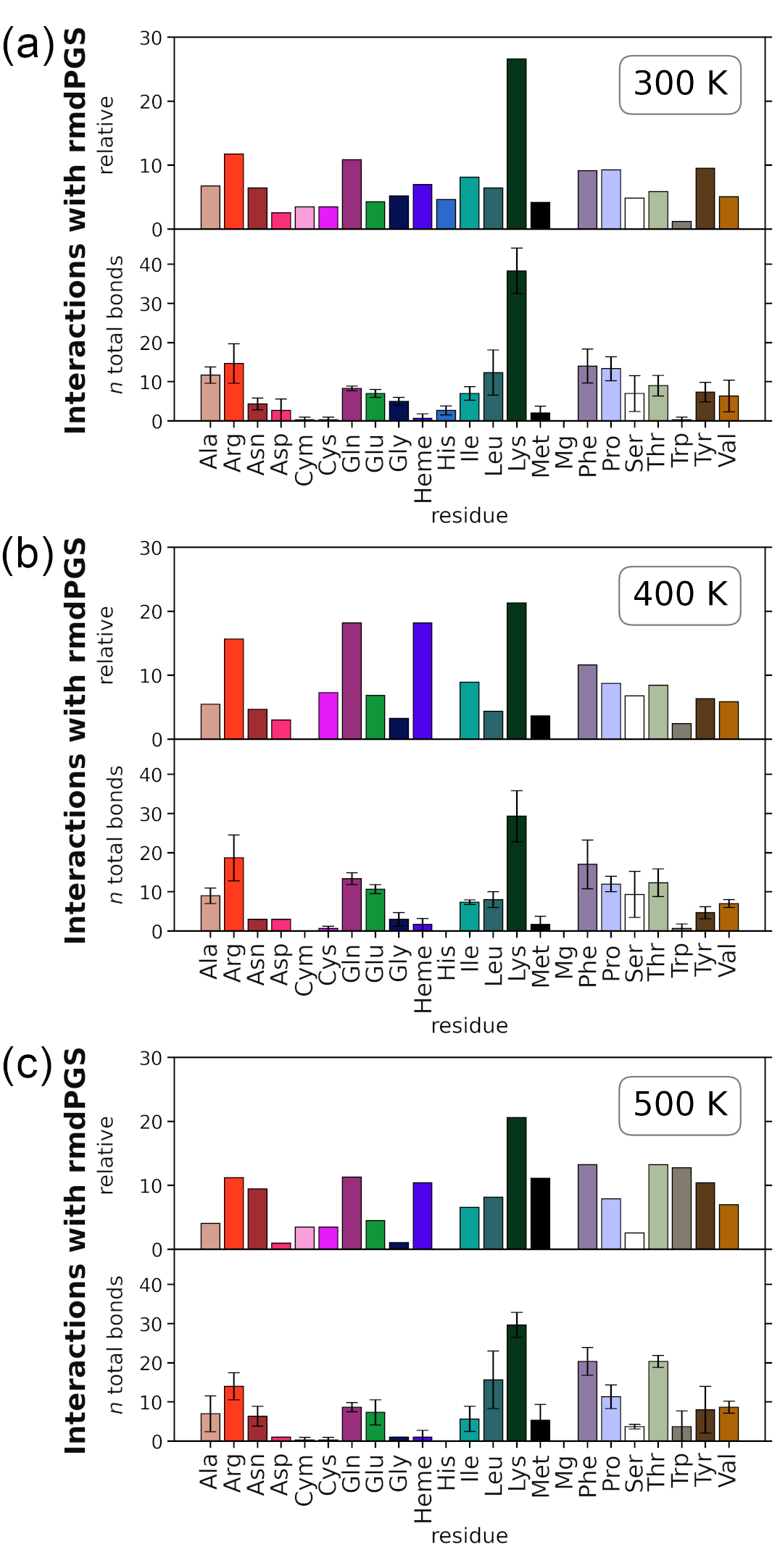

Supplement: Supplementary file 1 [file jp5c03518_si_001.zip › SI-Images/Conc_MD_Cvi_o_vac.png]

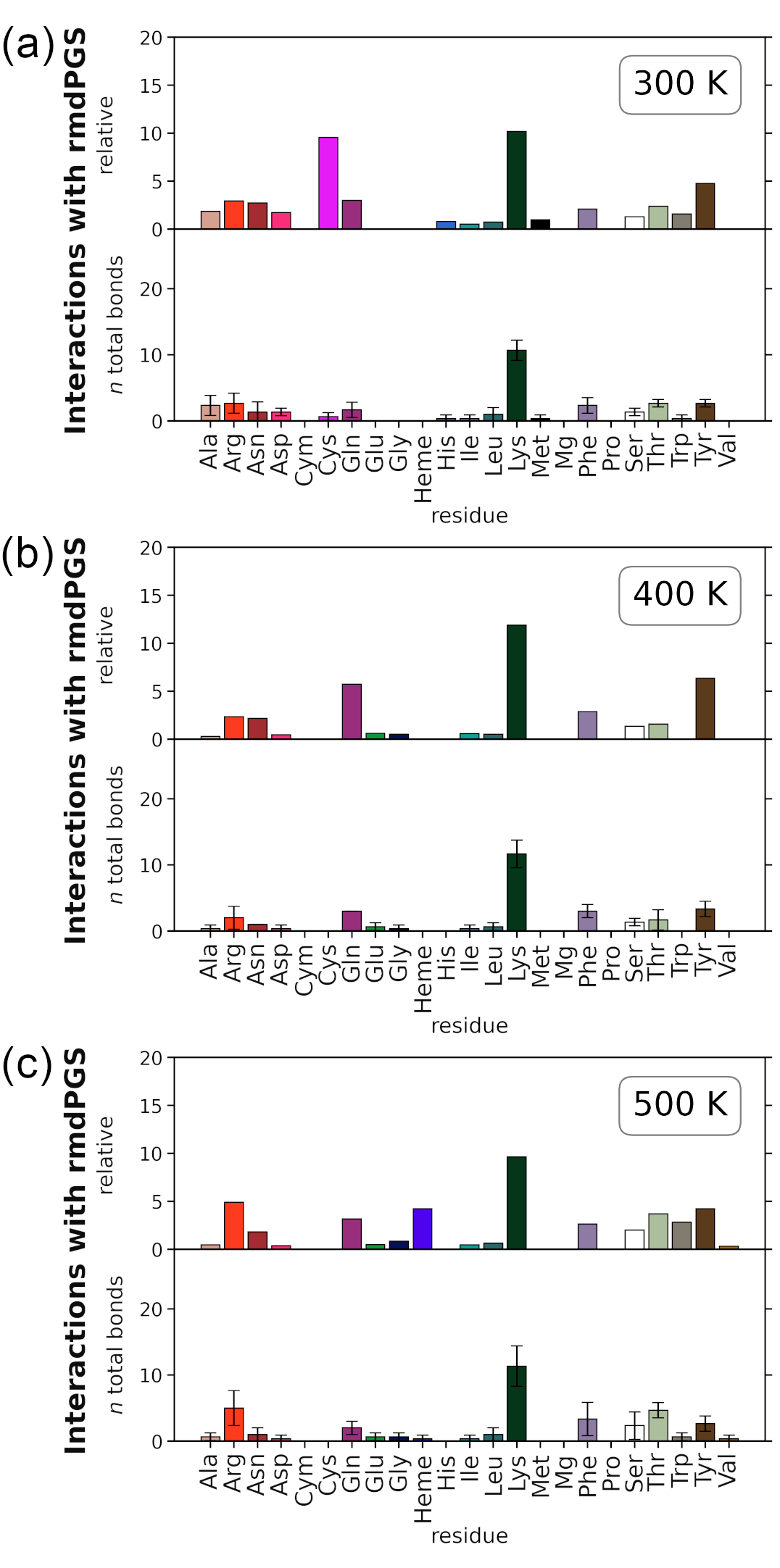

Supplement: Supplementary file 1 [file jp5c03518_si_001.zip › SI-Images/Conc_MD_Cvi_o2_vac.png]

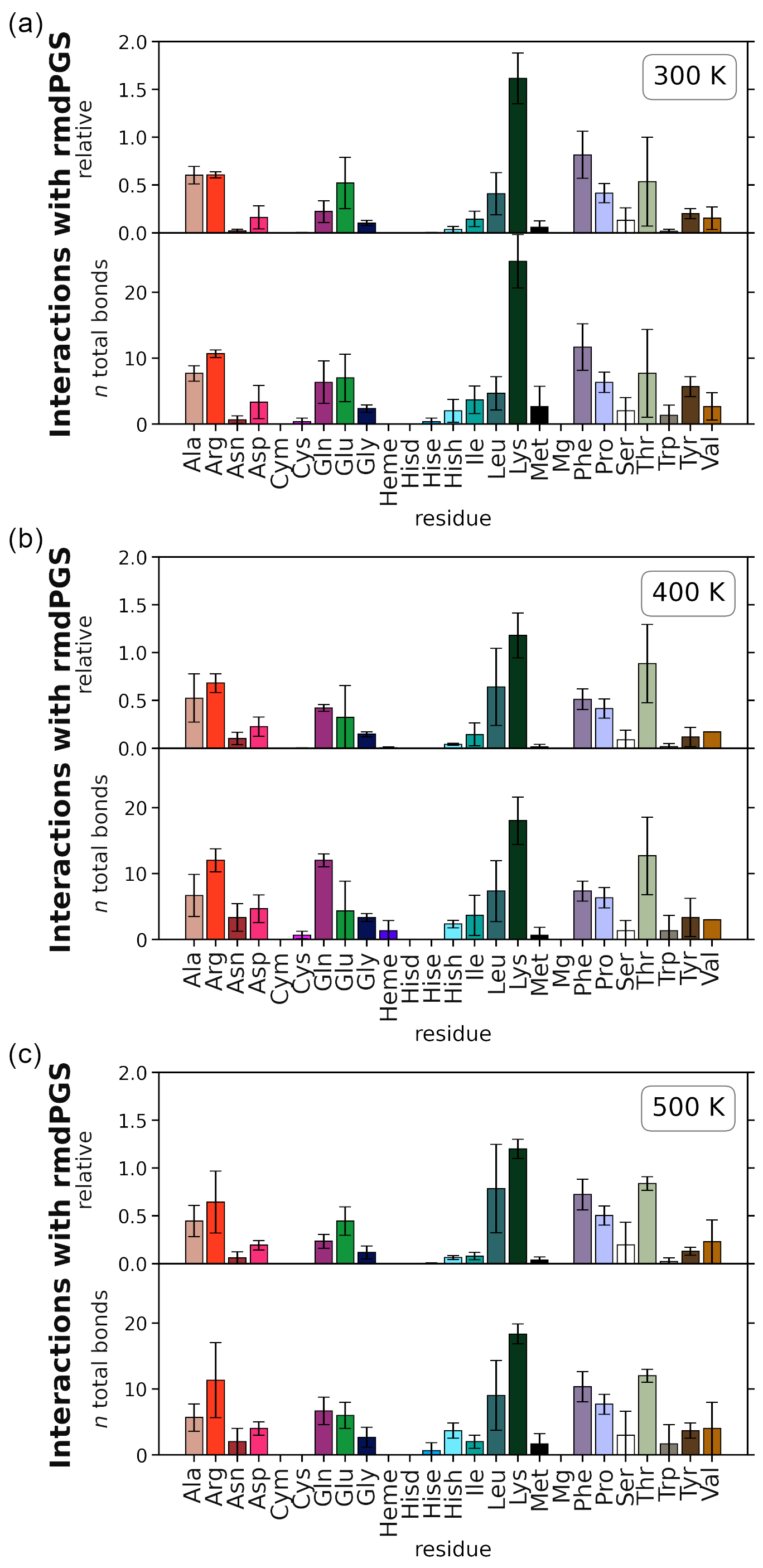

Supplement: Supplementary file 1 [file jp5c03518_si_001.zip › SI-Images/Conc_MD_Cvi_oh_vac.png]

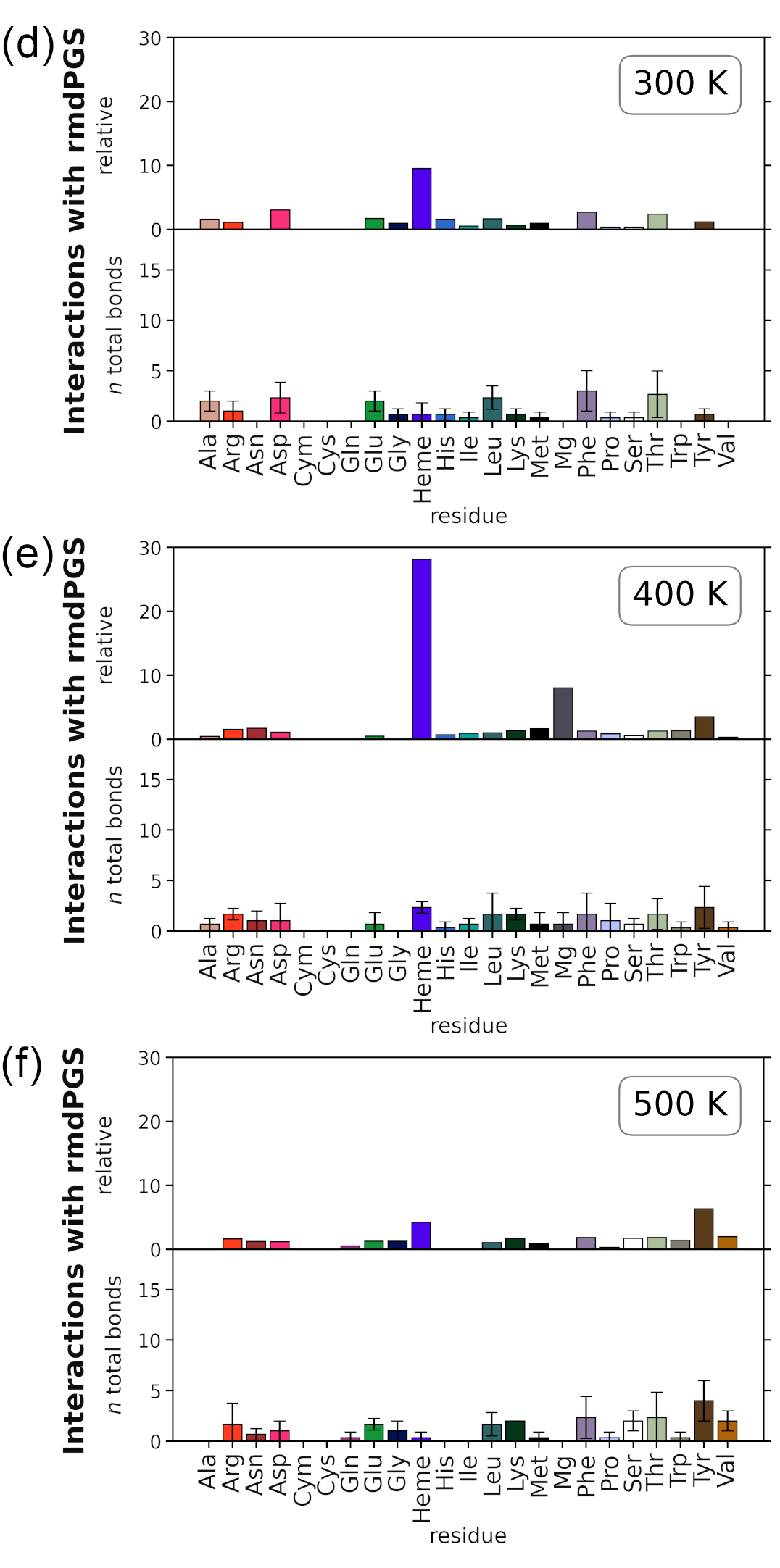

Supplement: Supplementary file 1 [file jp5c03518_si_001.zip › SI-Images/Conc_MD_Cvi_no_solv.png]

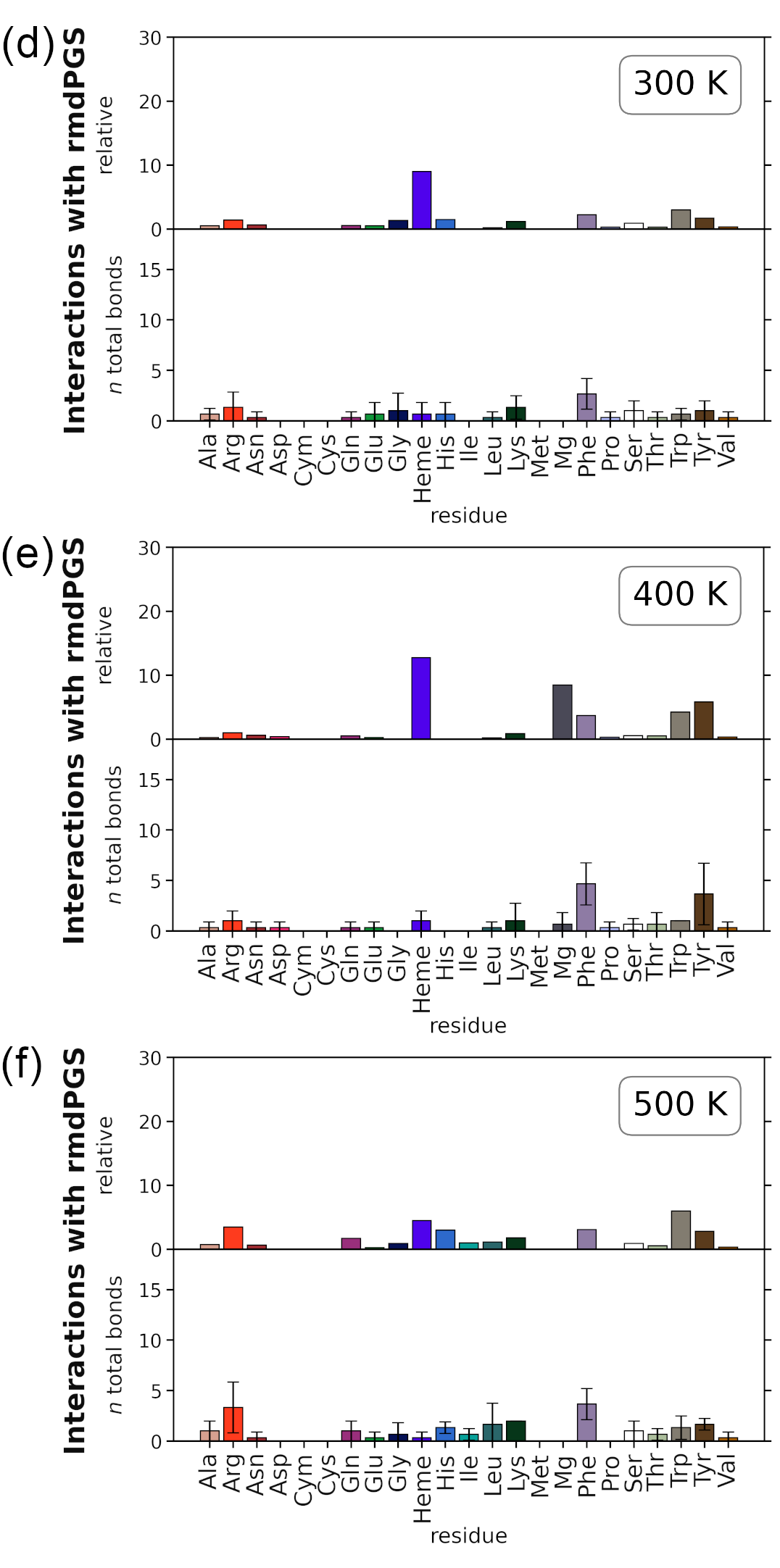

Supplement: Supplementary file 1 [file jp5c03518_si_001.zip › SI-Images/Conc_MD_Cvi_o2_solv.png]

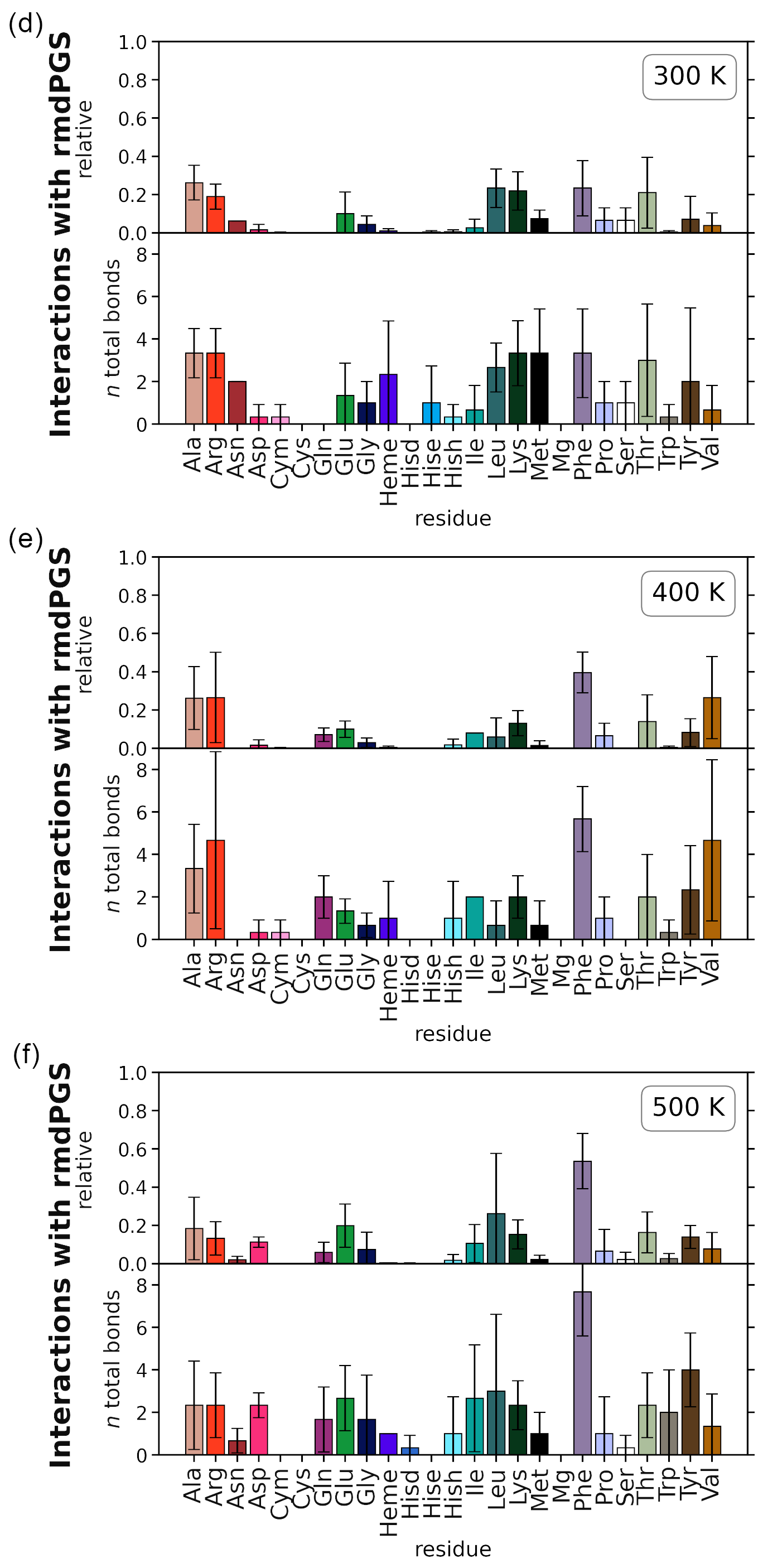

Supplement: Supplementary file 1 [file jp5c03518_si_001.zip › SI-Images/Conc_MD_Cvi_oh_solv.png]

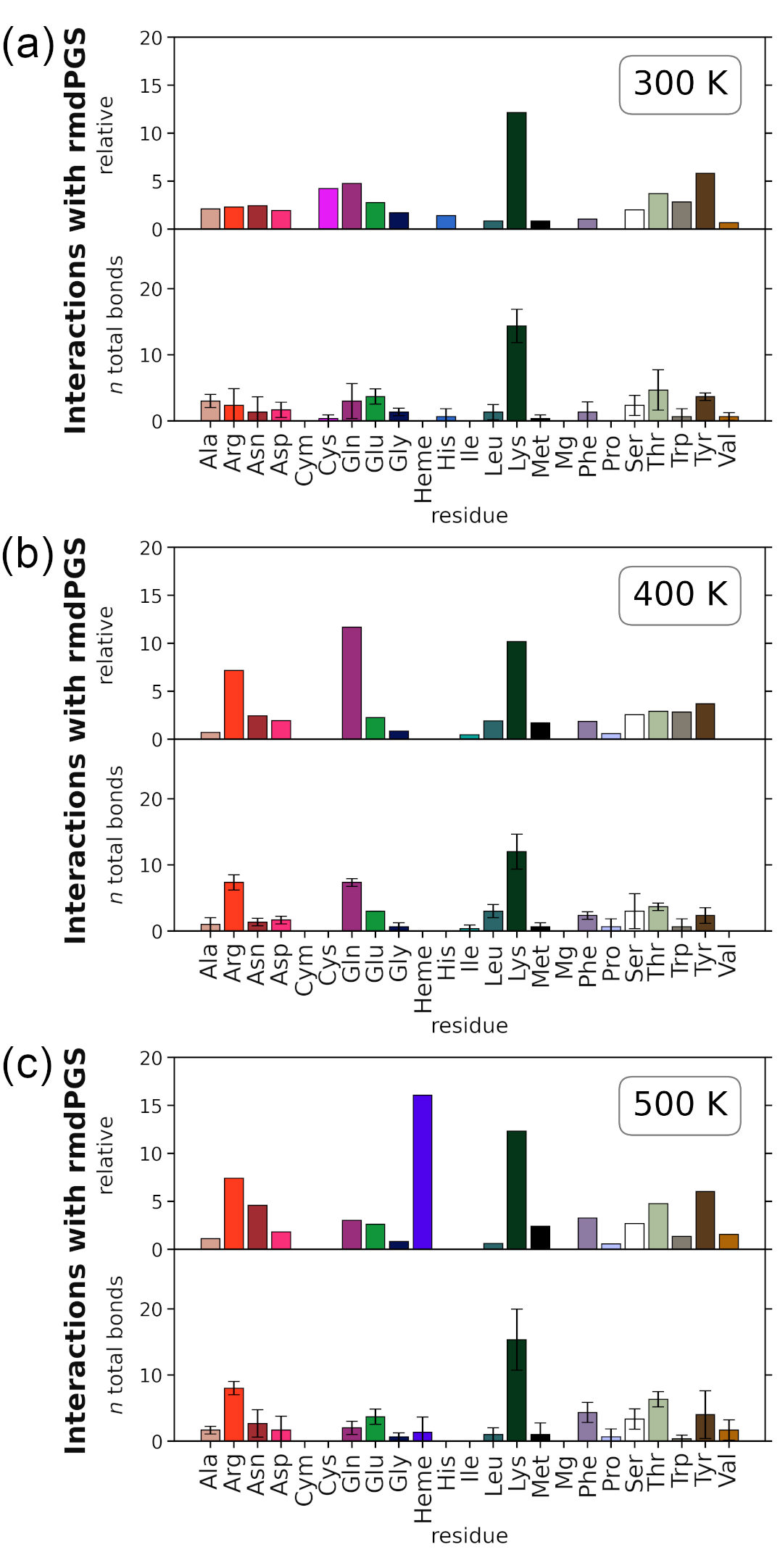

Supplement: Supplementary file 1 [file jp5c03518_si_001.zip › SI-Images/Conc_MD_Cvi_h2o2_vac.png]

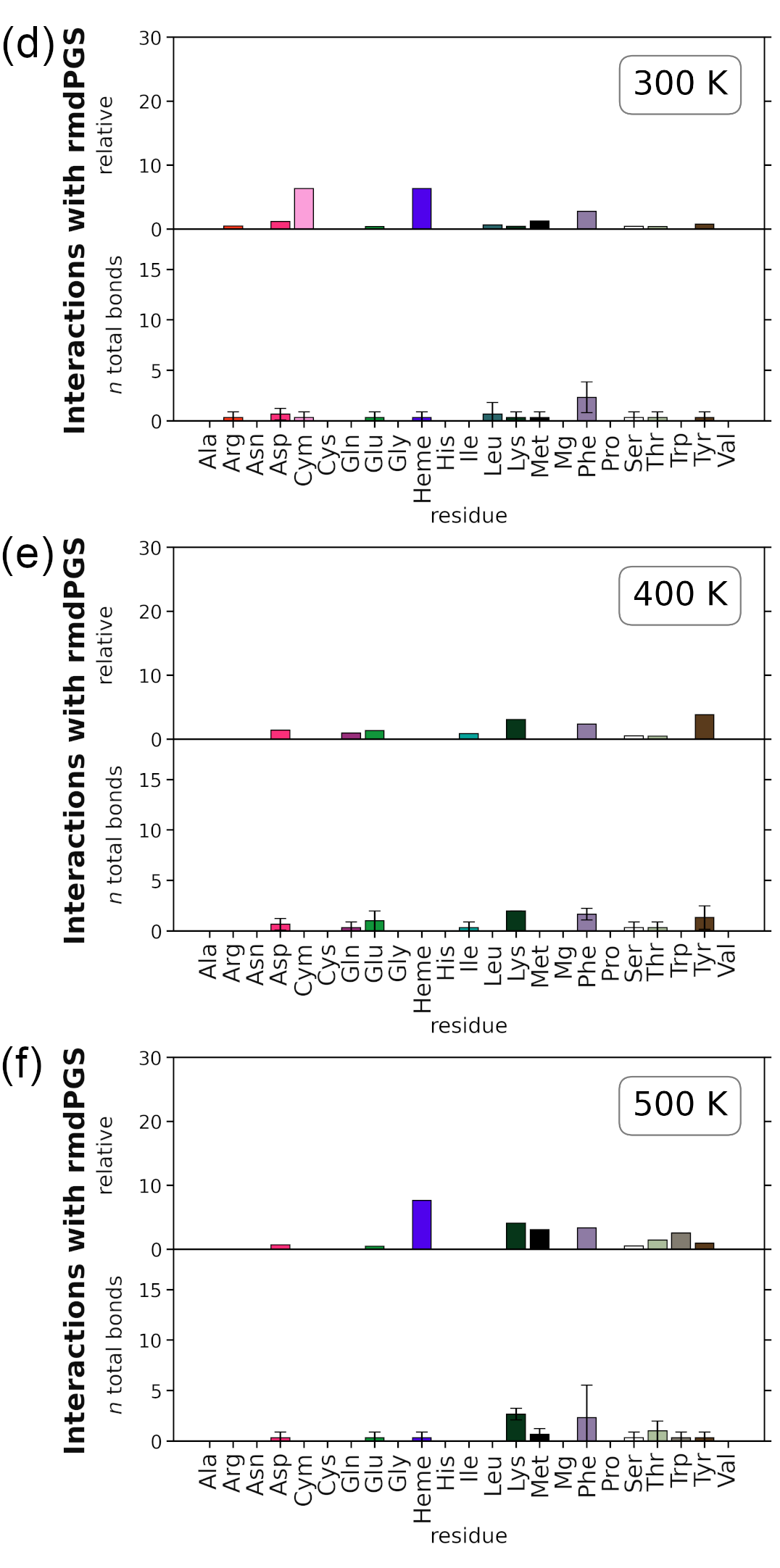

Supplement: Supplementary file 1 [file jp5c03518_si_001.zip › SI-Images/Conc_MD_Cvi_h2o2_solv.png]

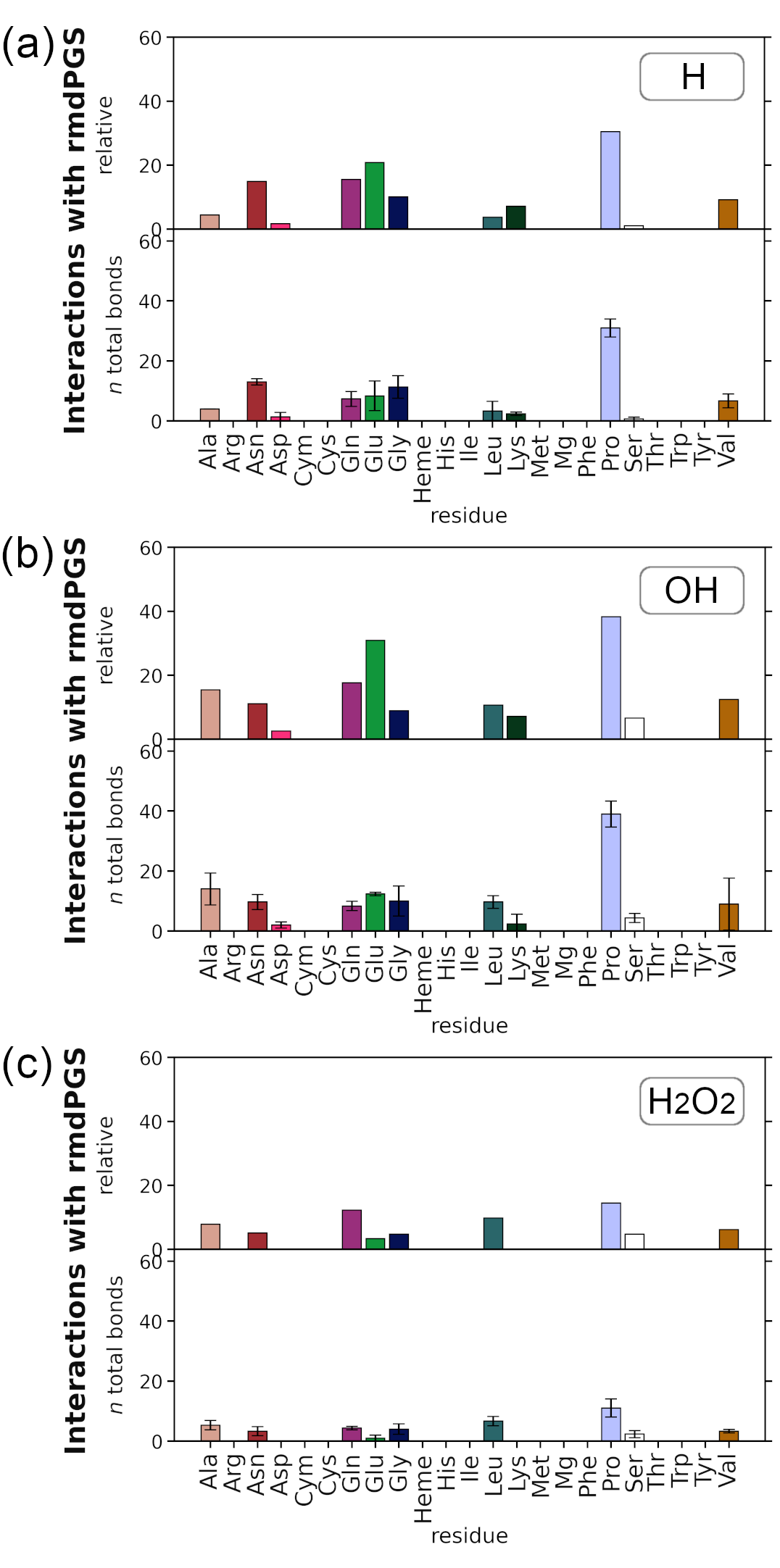

Supplement: Supplementary file 1 [file jp5c03518_si_001.zip › SI-Images/Conc_MD_Aae_h_h2o2_oh_vac.png]

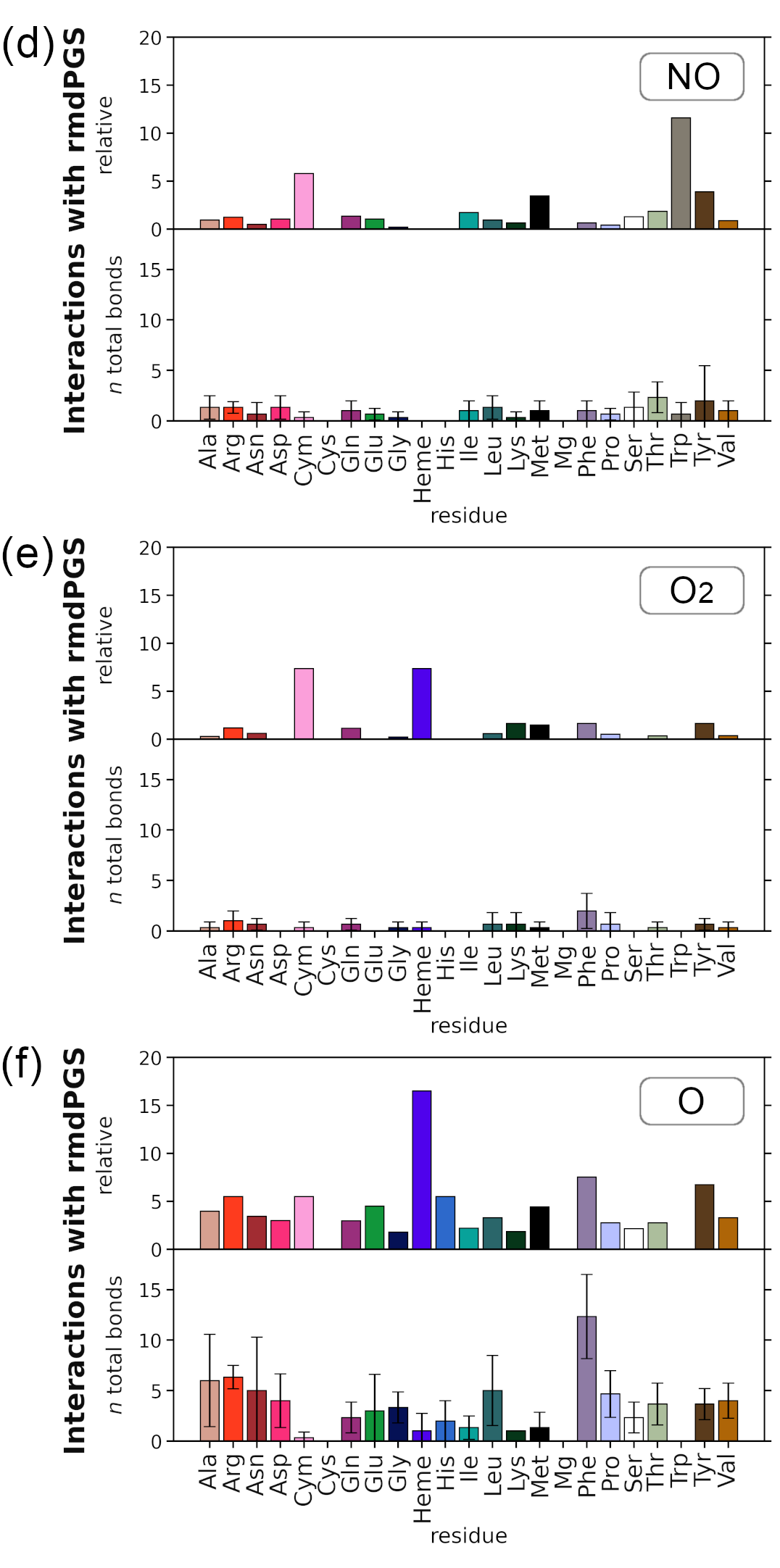

Supplement: Supplementary file 1 [file jp5c03518_si_001.zip › SI-Images/Conc_MD_Aae_no_o2_o_solv.png]

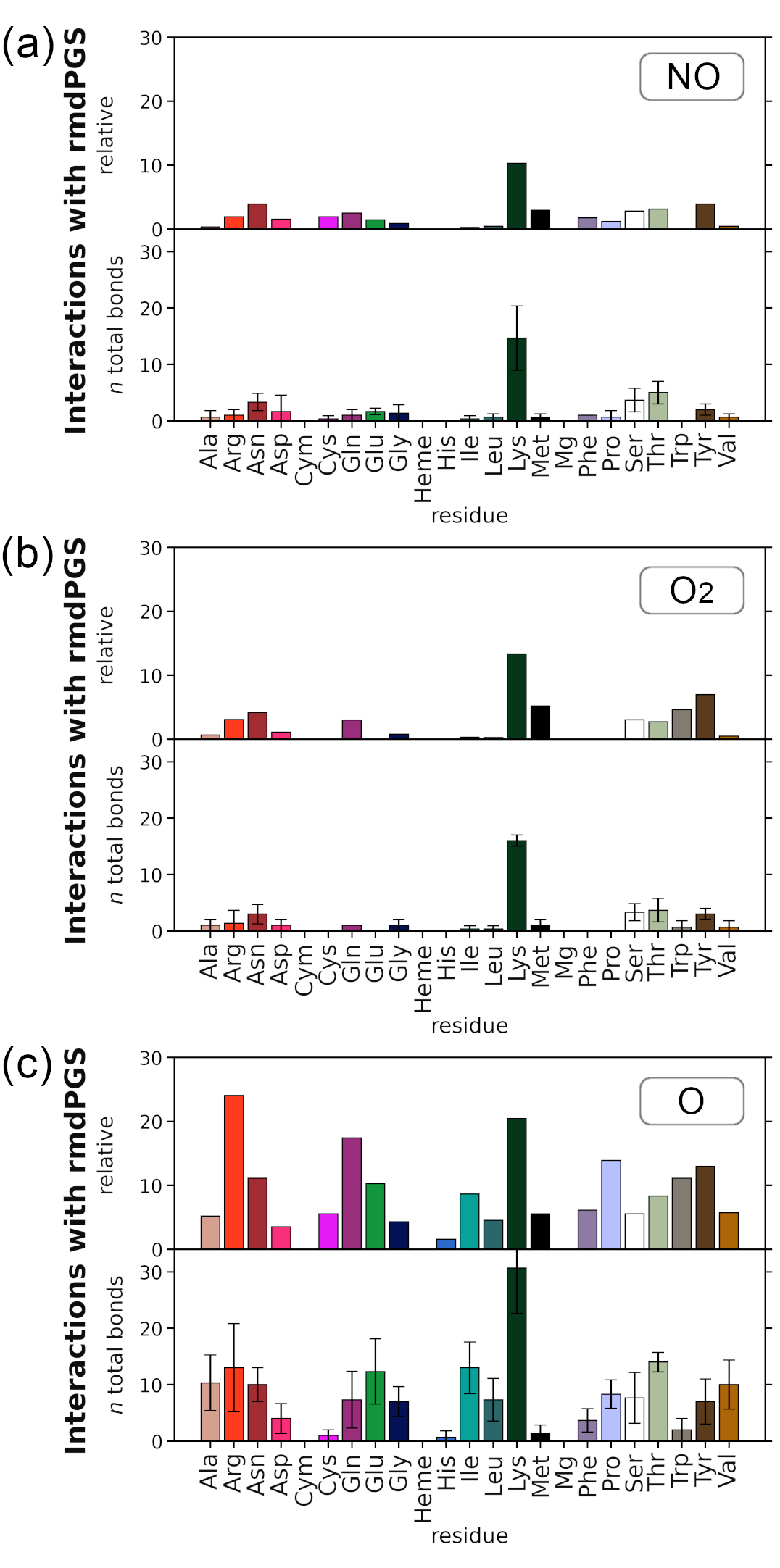

Supplement: Supplementary file 1 [file jp5c03518_si_001.zip › SI-Images/Conc_MD_GapA_no_o2_o_vac.png]

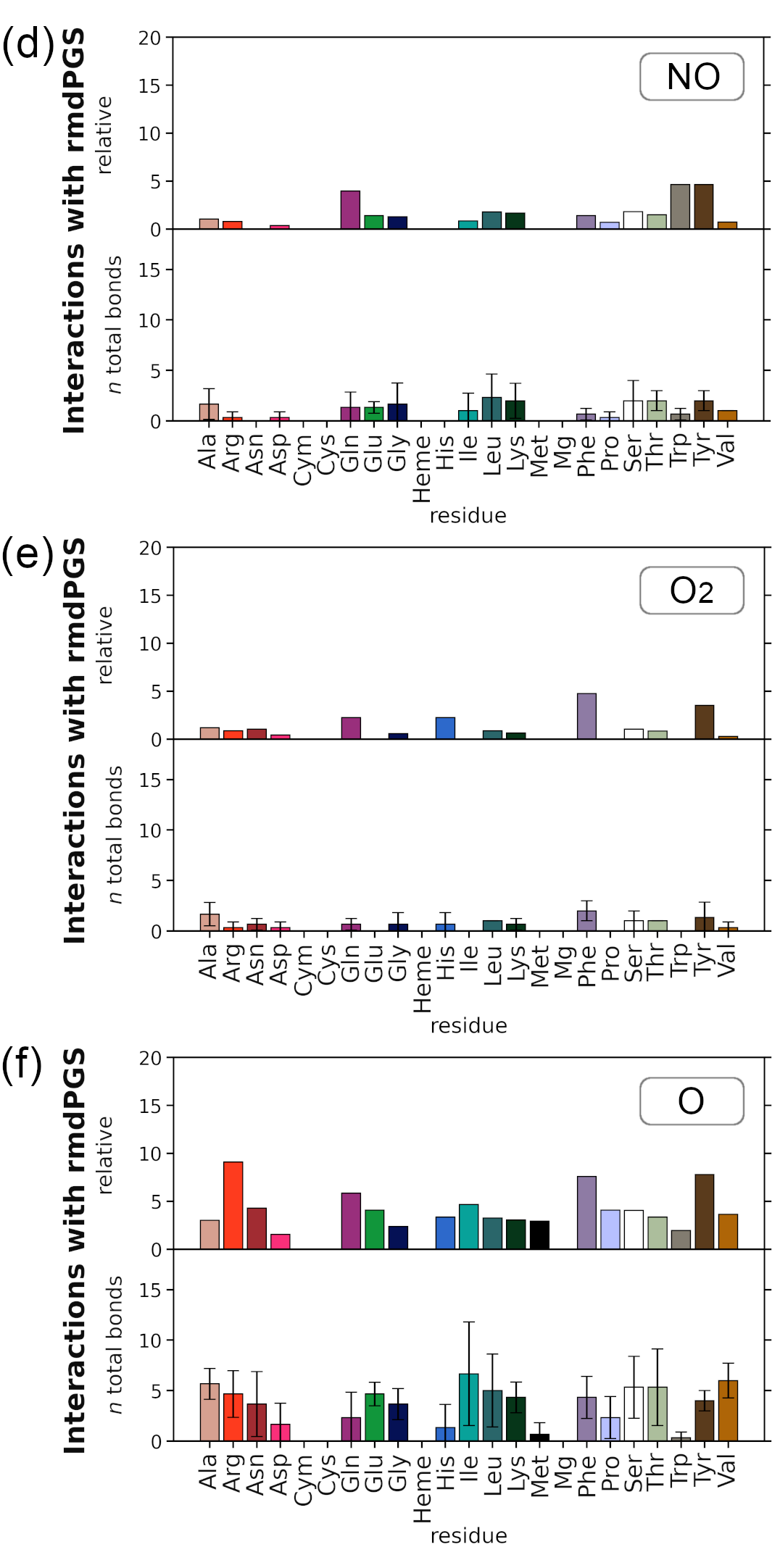

Supplement: Supplementary file 1 [file jp5c03518_si_001.zip › SI-Images/Conc_MD_GapA_no_o2_o_solv.png]

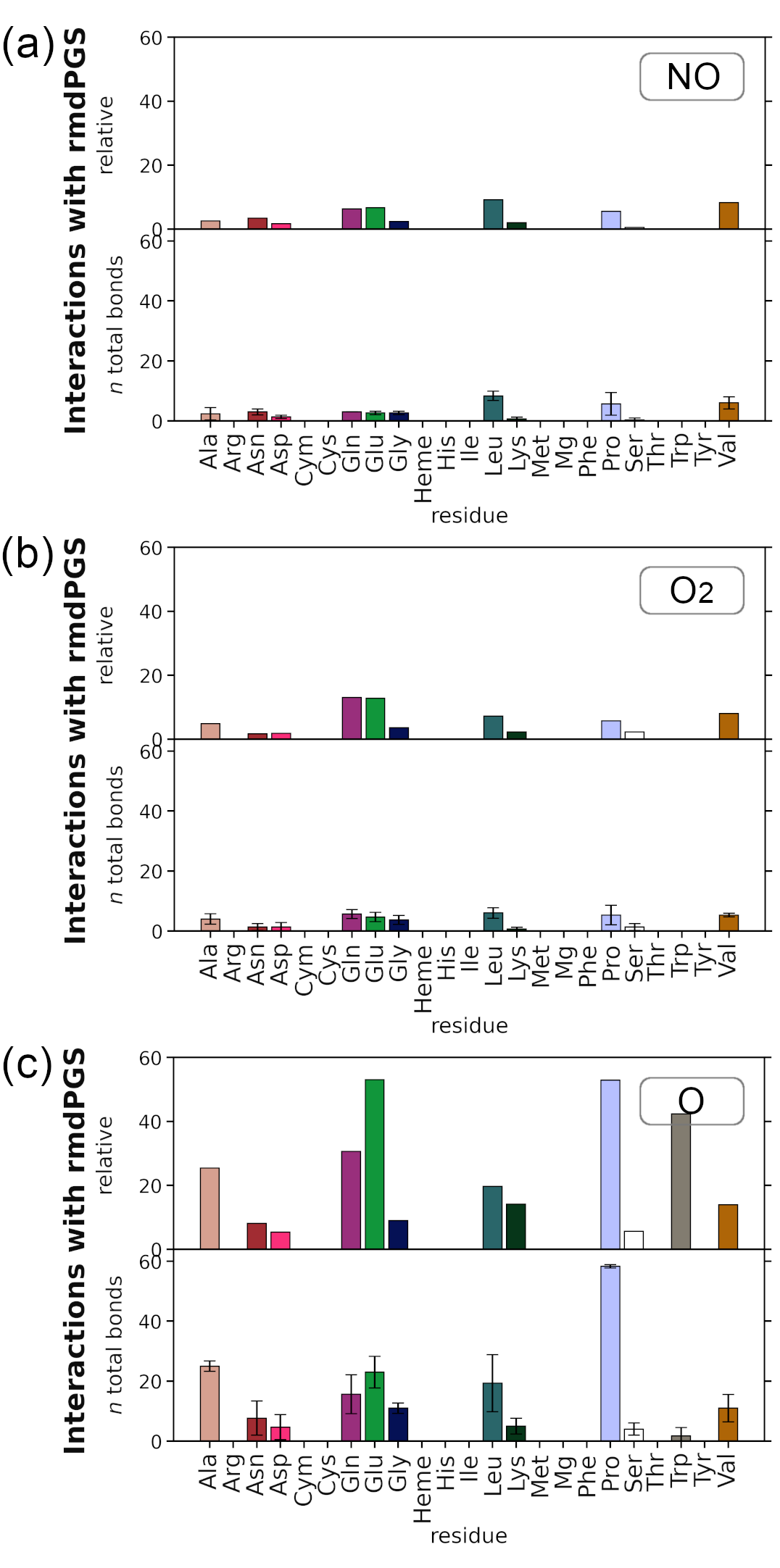

Supplement: Supplementary file 1 [file jp5c03518_si_001.zip › SI-Images/Conc_MD_Aae_no_o2_o_vac.png]

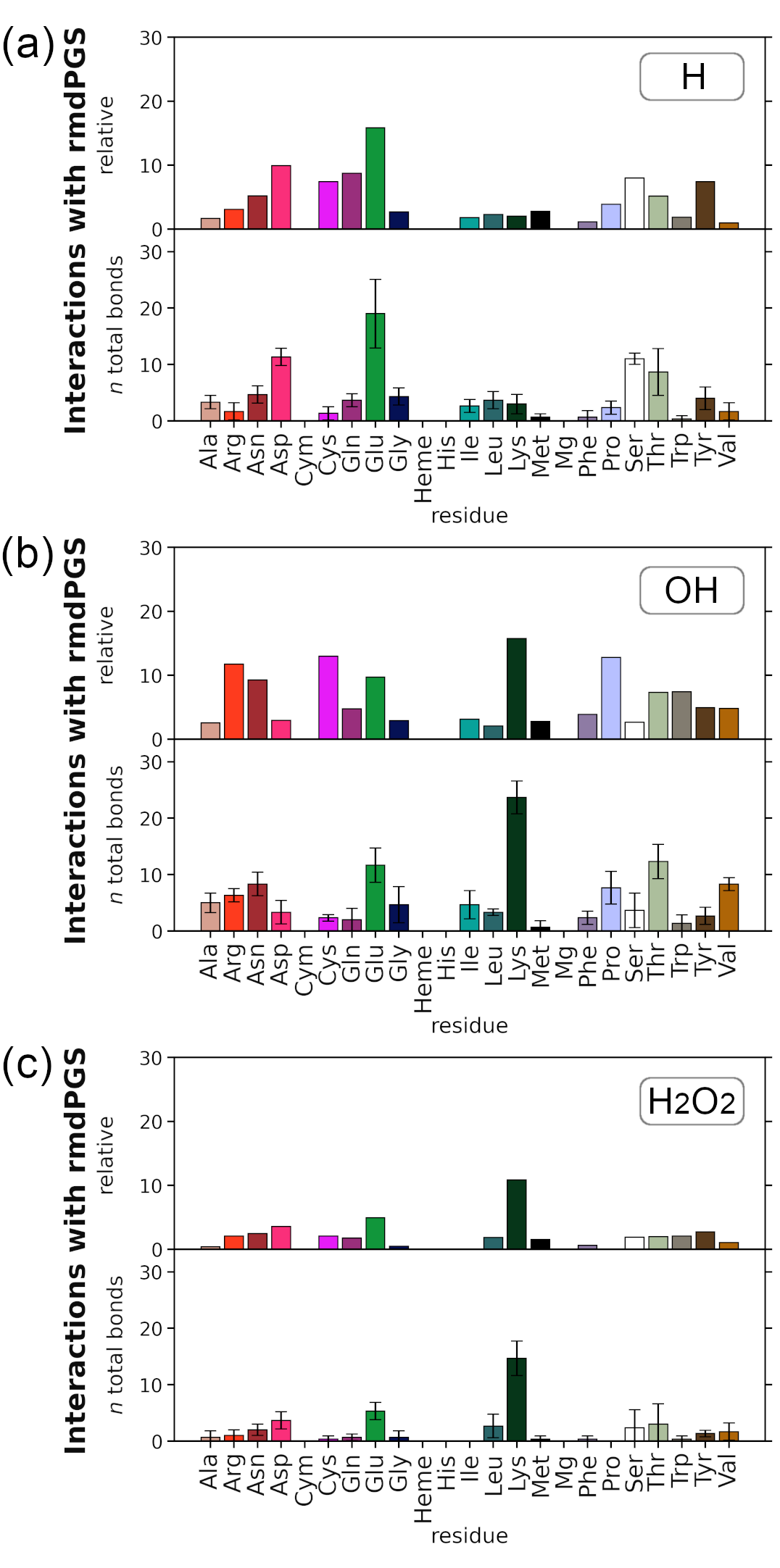

Supplement: Supplementary file 1 [file jp5c03518_si_001.zip › SI-Images/Conc_MD_GapA_h_h2o2_oh_vac.png]

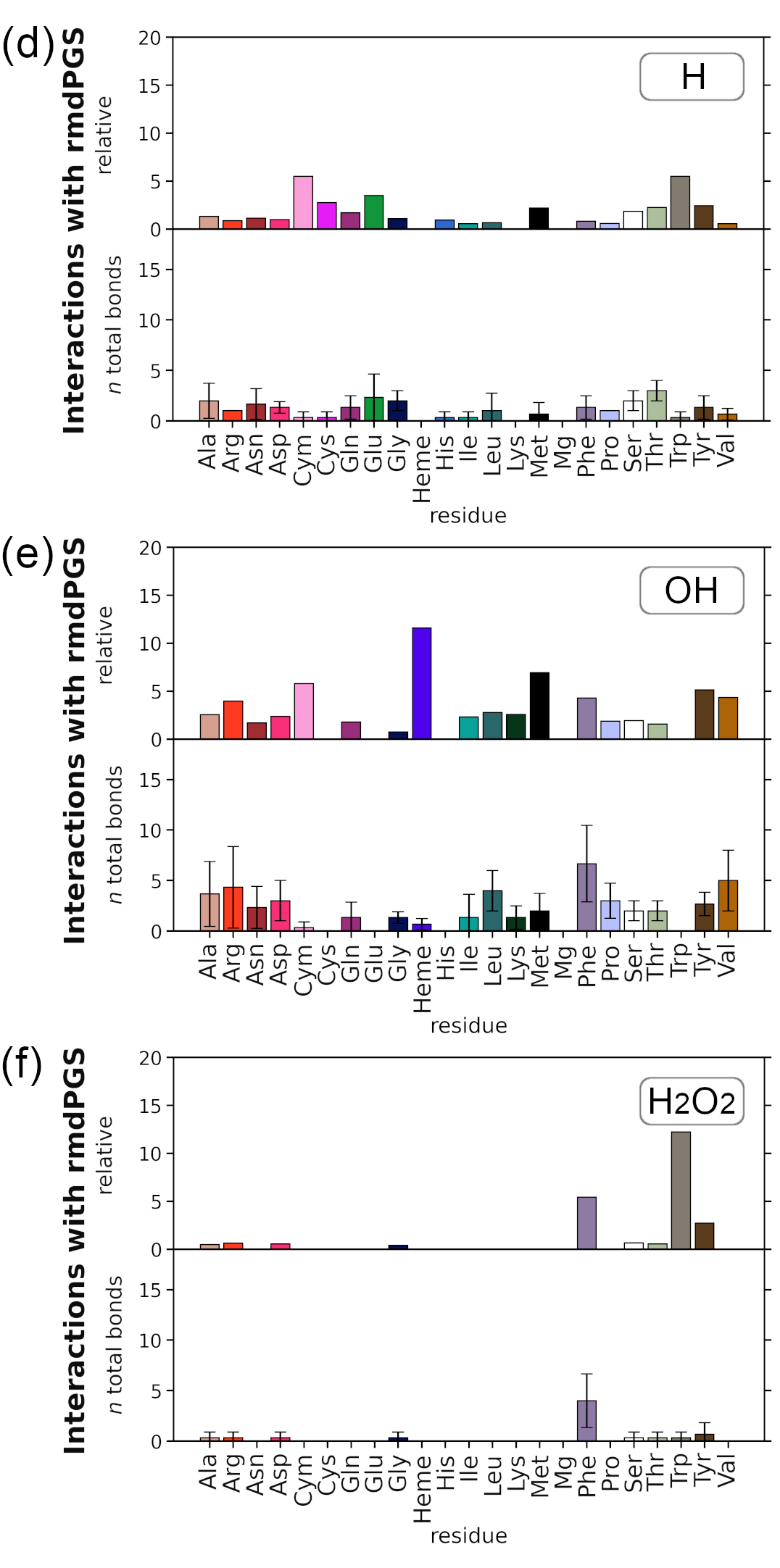

Supplement: Supplementary file 1 [file jp5c03518_si_001.zip › SI-Images/Conc_MD_Aae_h_h2o2_oh_solv.png]

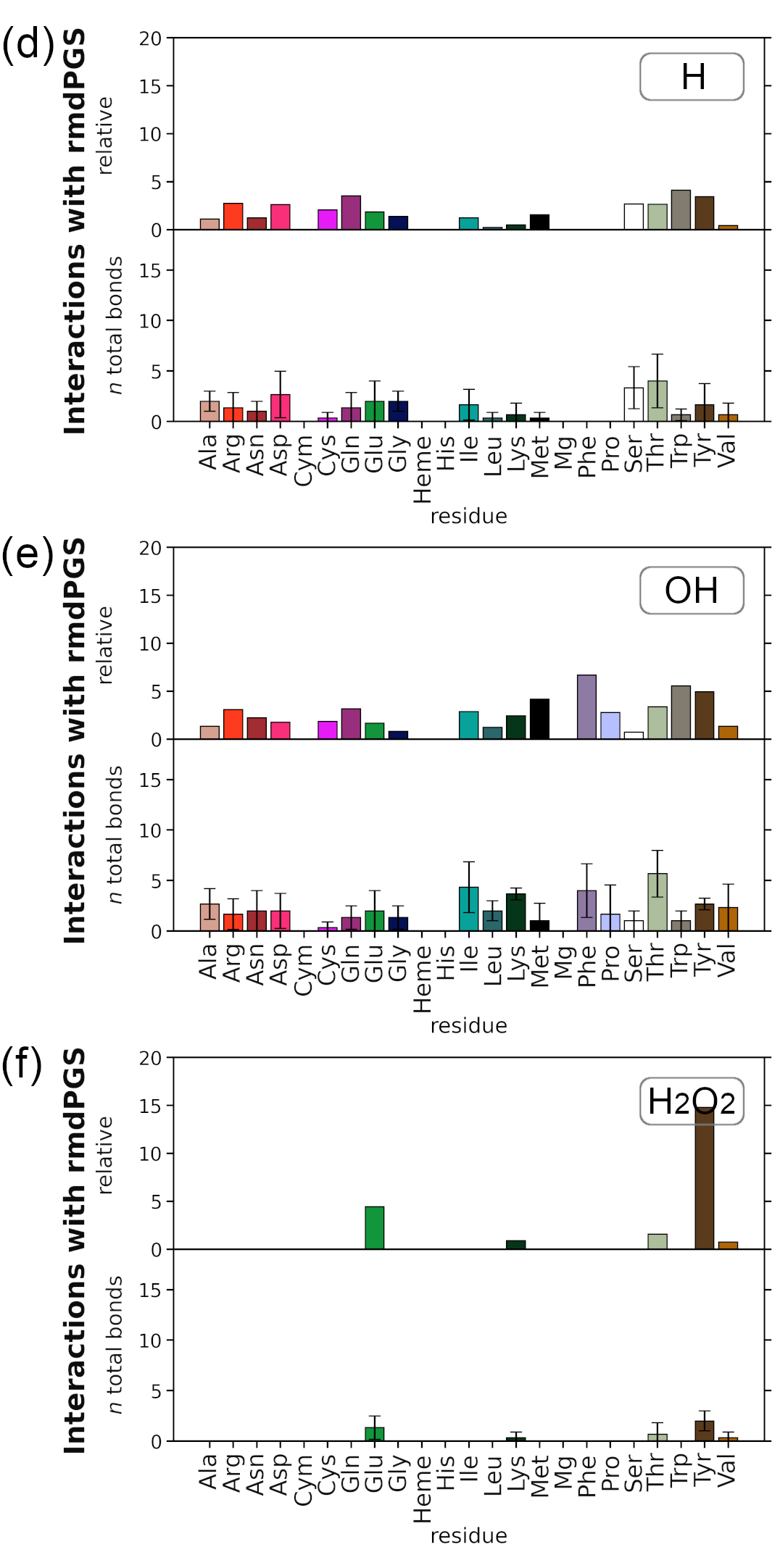

Supplement: Supplementary file 1 [file jp5c03518_si_001.zip › SI-Images/Conc_MD_GapA_h_h2o2_oh_solv.png]

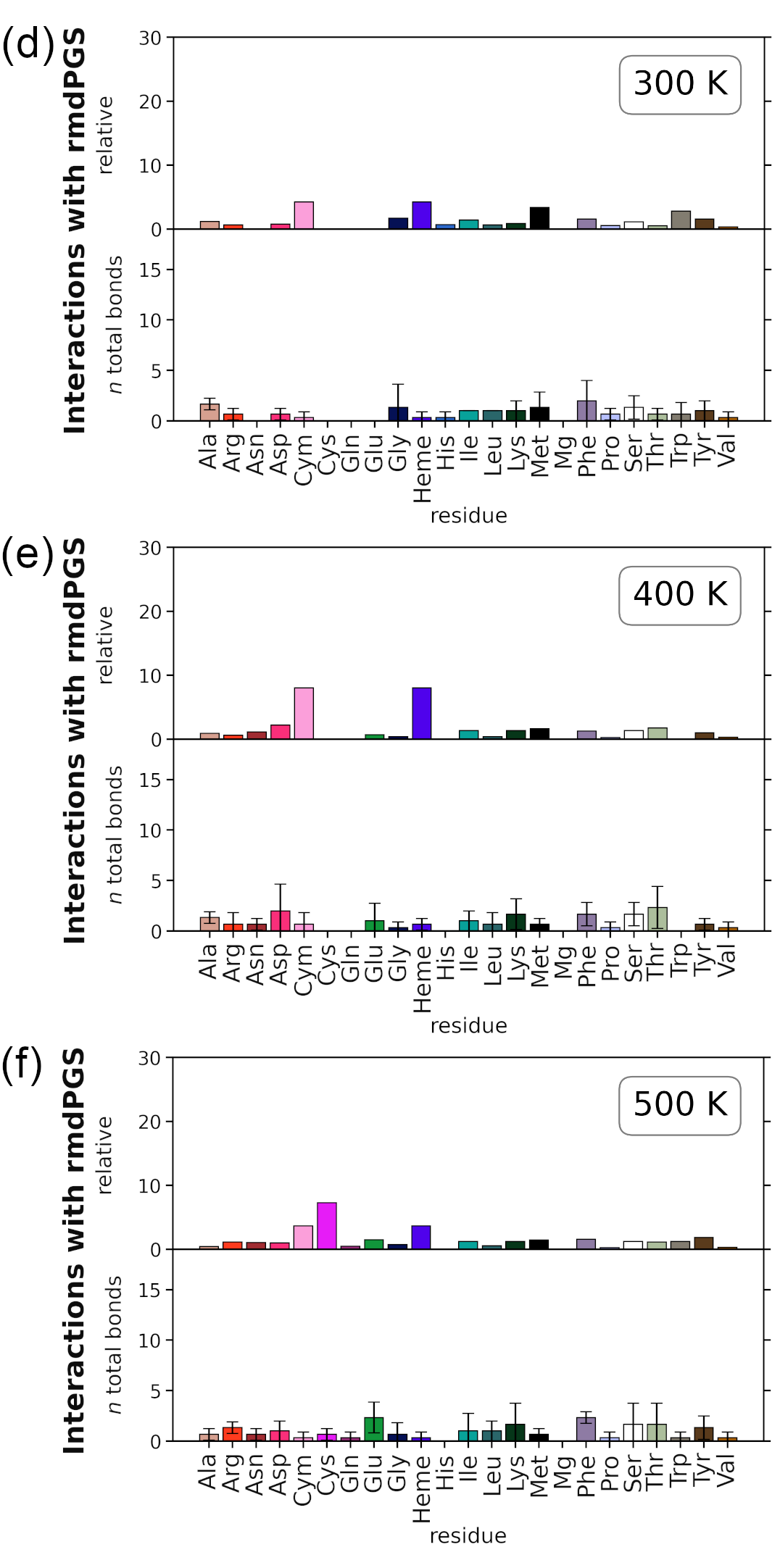

Supplement: Supplementary file 1 [file jp5c03518_si_001.zip › SI-Images/Conc_MD_Cvi_h_solv.png]

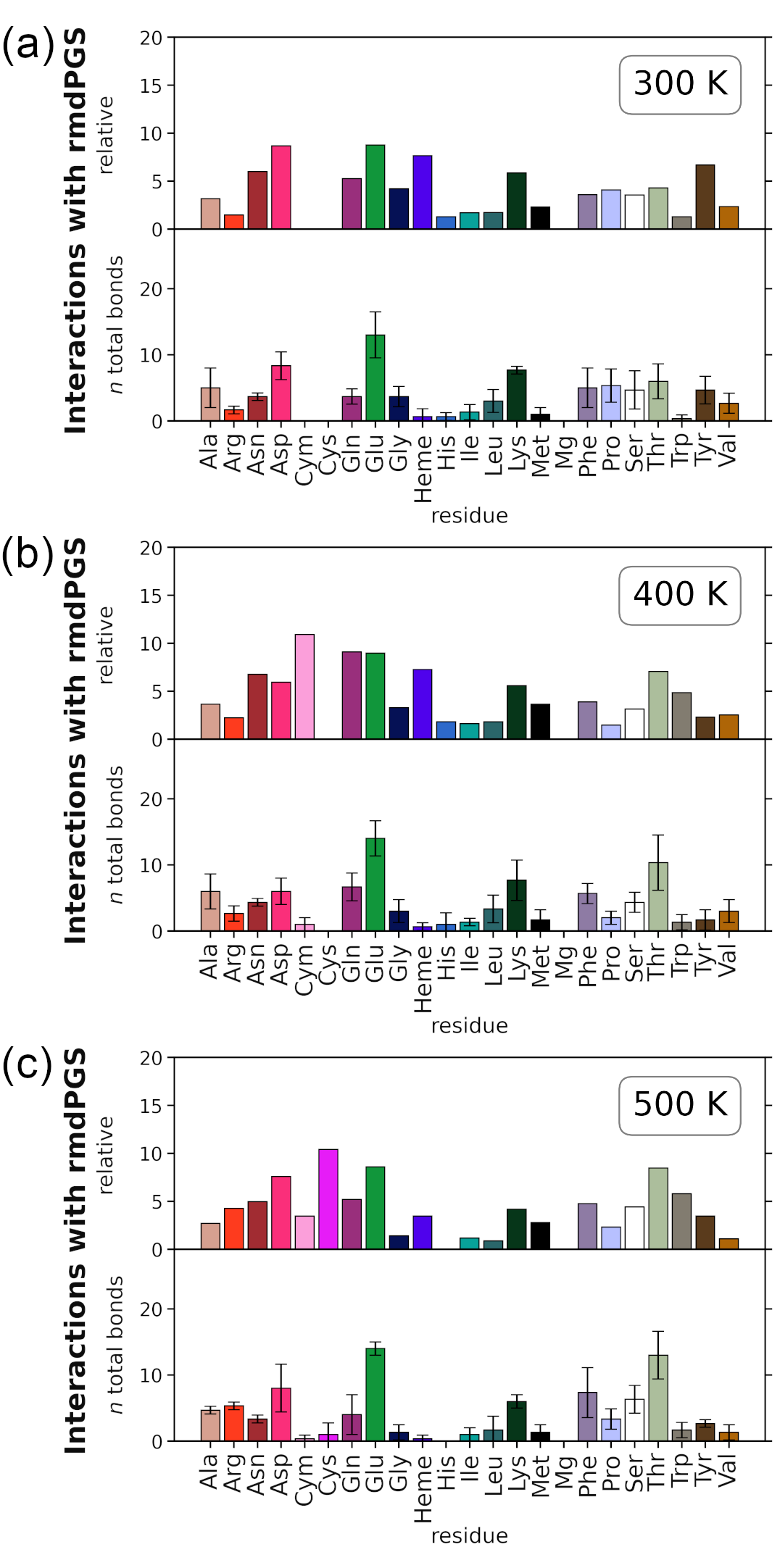

Supplement: Supplementary file 1 [file jp5c03518_si_001.zip › SI-Images/Conc_MD_Cvi_h_vac.png]

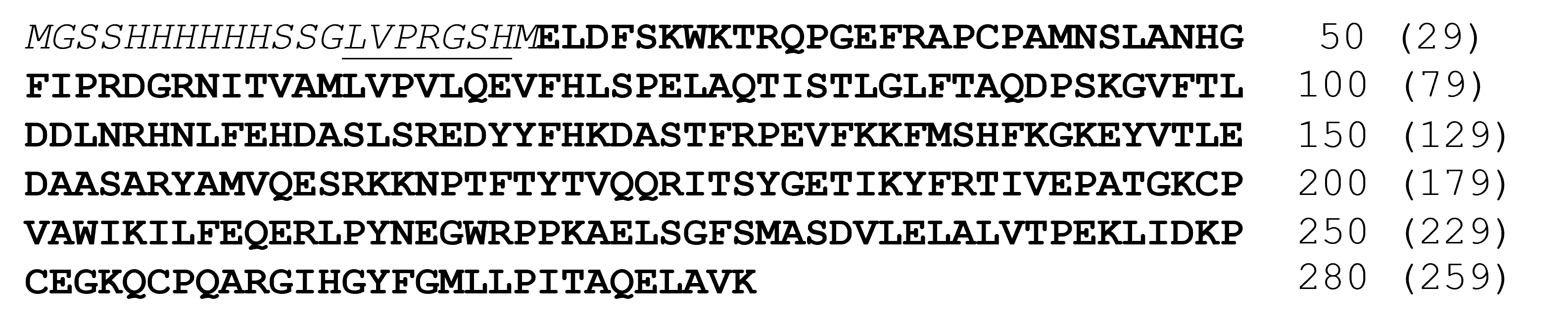

Supplement: Supplementary file 1 [file jp5c03518_si_001.zip › SI-Images/Sequence_CviUPO.png]
