# Supplementary material for: Phenylalanine Modification in Plasma-Driven Biocatalysis Revealed by Solvent Accessibility and Reactive Dynamics in Combination with Protein Mass Spectrometry
Source: J Phys Chem B. 2025 Oct 23;129(44):11374–86. doi: 10.1021/acs.jpcb.5c03518 (PMC12598871; doi:10.1021/acs.jpcb.5c03518)
Supplement: Supplementary file 2 [file jp5c03518_si_002.pdf]

Phenylalanine modification in plasma-driven biocatalysis revealed by solvent accessibility and reactive dynamics in combination with protein mass spectrometry

Hanna-Friederike Poggemann,[†] Sabrina Klopsch,[‡] Simon Homann,[†] Tim Dirks,[‡] Sina Schäkermann,[‡] Julia E. Bandow,[‡] Timo Jacob,[†,¶,§] and Christoph Jung[*,†,¶,§]

†*Institute of Electrochemistry, Ulm University, D-89081 Ulm, Germany*

‡*Applied Microbiology, Faculty of Biology and Biotechnology, Ruhr University Bochum, D-44801 Bochum, Germany*

¶*Karlsruhe Institute of Technology (KIT), D-76049 Karlsruhe, Germany*

§*Helmholtz Institute Ulm (HIU) Electrochemical Energy Storage, D-89081 Ulm, Germany*

E-mail: christoph.jung@kit.edu

Supporting information

Amino acid sequence of *Cvi*UPO

*MGSSHHHHHHSSG<u>LVPRGSH</u>M***ELDFSKWKTRQPGEFRAPCPAMNSLANHG**	50	(29)
**FIPRDGRNITVAMLVPVLQEVFHLSPELAQTISTLGLFTAQDPSKGVFTL**	100	(79)
**DDLNRHNLFEHDASLSREDYYFHKDASTFRPEVFKKFMSHFKGKEYVTLE**	150	(129)
**DAASARYAMVQESRKKNPTFTYTVQQRITSYGETIKYFRTIVEPATGKCP**	200	(179)
**VAWIKILFEQERLPYNEGWRPPKAELSGFSMASDVLELALVTPEKLIDKP**	250	(229)
**CEGKQCPQARGIHGYFGMLLPITAQELAVK**	280	(259)

Figure S1: Amino acid sequence of the *Collariella virescens* UPO used in the experimental study. The poly-His tail sequence is shown in italics and thrombin recognition sequence is underlined. The amino acid positions without the poly-His tail sequence (utilized in the text) are shown in brackets.

Explanation to the species nature in ReaxFF

It is important to note that, due to the limitations of the reactive molecular dynamics approach, modeling real plasma species remains a significant challenge. Fundamentally, the primary limitation arises from the nature of the datasets used to parameterize force fields (FFs). High-quality FFs are typically derived from quantum mechanical (QM) calculations, which are inherently restricted to ground-state descriptions. Consequently, any molecular dynamics (MD)-scale representation of plasma species is necessarily limited in accuracy. While the explicit treatment of plasma species within the QM regime is a distinct and important topic, it falls outside the scope of the present study. In a similar context, and most importantly, the ReaxFF employed in this study was not developed by us.[1] Therefore, we have no direct knowledge of whether specific species, such as OH radicals or OH ions, were included in the original training dataset. Even if such species were incorporated, the current ReaxFF framework does not explicitly treat electrons, making a rigorous distinction between radicals and ions fundamentally challenging. Efforts to address this issue, such as the development of eReaxFF, are ongoing, but they represent a separate and complex research direction. By

design, ReaxFF relies on the bond-order formalism to simulate bond dissociation and formation, setting it apart from conventional FFs. In addition to bond order, ReaxFF employs dynamic charge equilibration, allowing for the tracking of charge redistribution. For OH species, this means that charge variations in the surrounding environment or counter ions may provide some insights into partial charge changes.[2–4] For instance, an analysis of the bond order and partial charge variations among OH species could suggest possible differences in their chemical nature. However, at a fundamental level, the reliability of ReaxFF is entirely dependent on the quality of the training dataset and the methodology used in its parametrization. In particular, charge-related properties are known to be less accurate within this framework. As a result, distinguishing between OH ions and OH radicals in our study remains highly uncertain.

In summary, the ReaxFF employed here lacks the precision required to unambiguously differentiate OH radicals from OH ions. This issue was also addressed in a study by W. Zhang and A. van Duin, where they find that in reaction of unsolvated OH– with methane the OH anion reacts like the OH radical.[5] Consequently, any conclusions drawn from these simulations should be interpreted with caution.

While the limitations of the force field should be considered, they only moderately affect the interpretation of the results. This is because, in a real system, it can be assumed that species from the plasma effluent diffuse through several milliliters of solvent before interacting with the enzyme, likely losing their excitation and possibly their radical characteristics by the time they reach the enzyme.

SASA interaction analysis

*Cvi*UPO

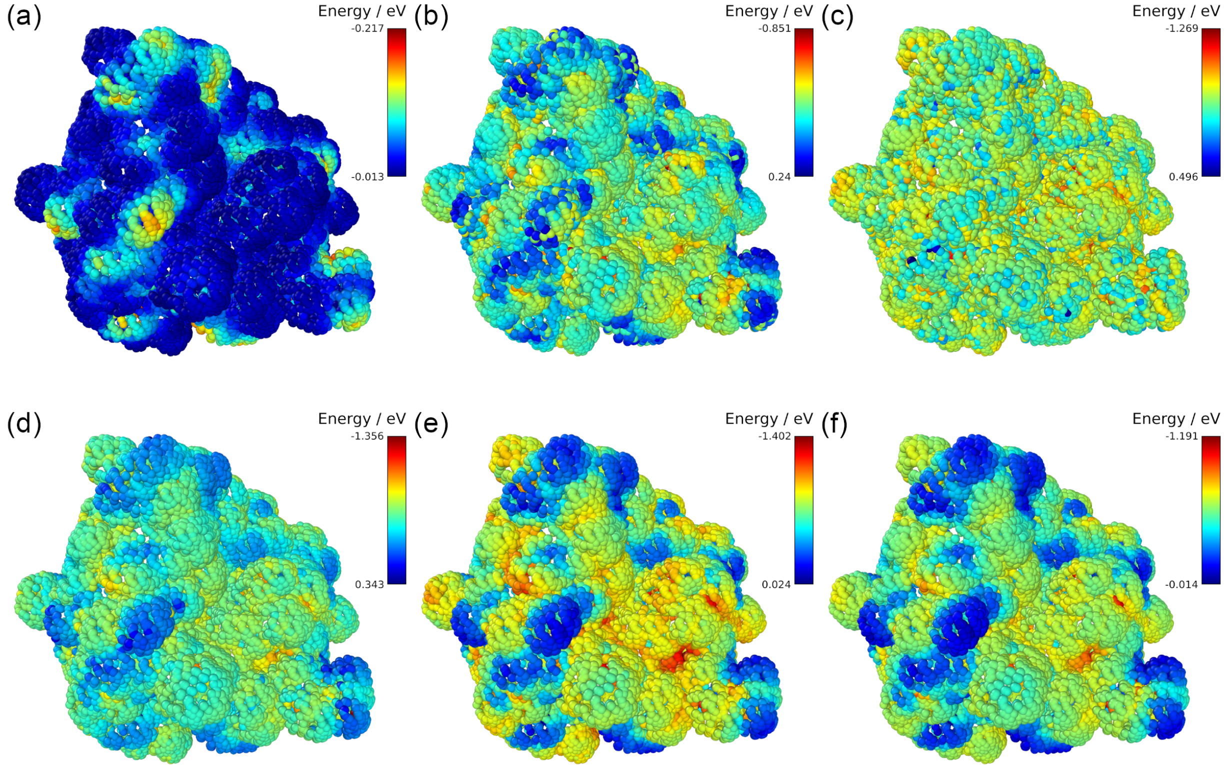

Figure S2: Interaction maps from SASA analysis for the enzyme *Cvi*UPO at 300 K with (a) H and (b) OH, (c) H_2O_2, (d) NO, (e) O_2 and (f) O. The colour bar in the upper right corner of each interaction map indicates the value of the interaction energy. More negative values are indicated in red, less negative values in blue.

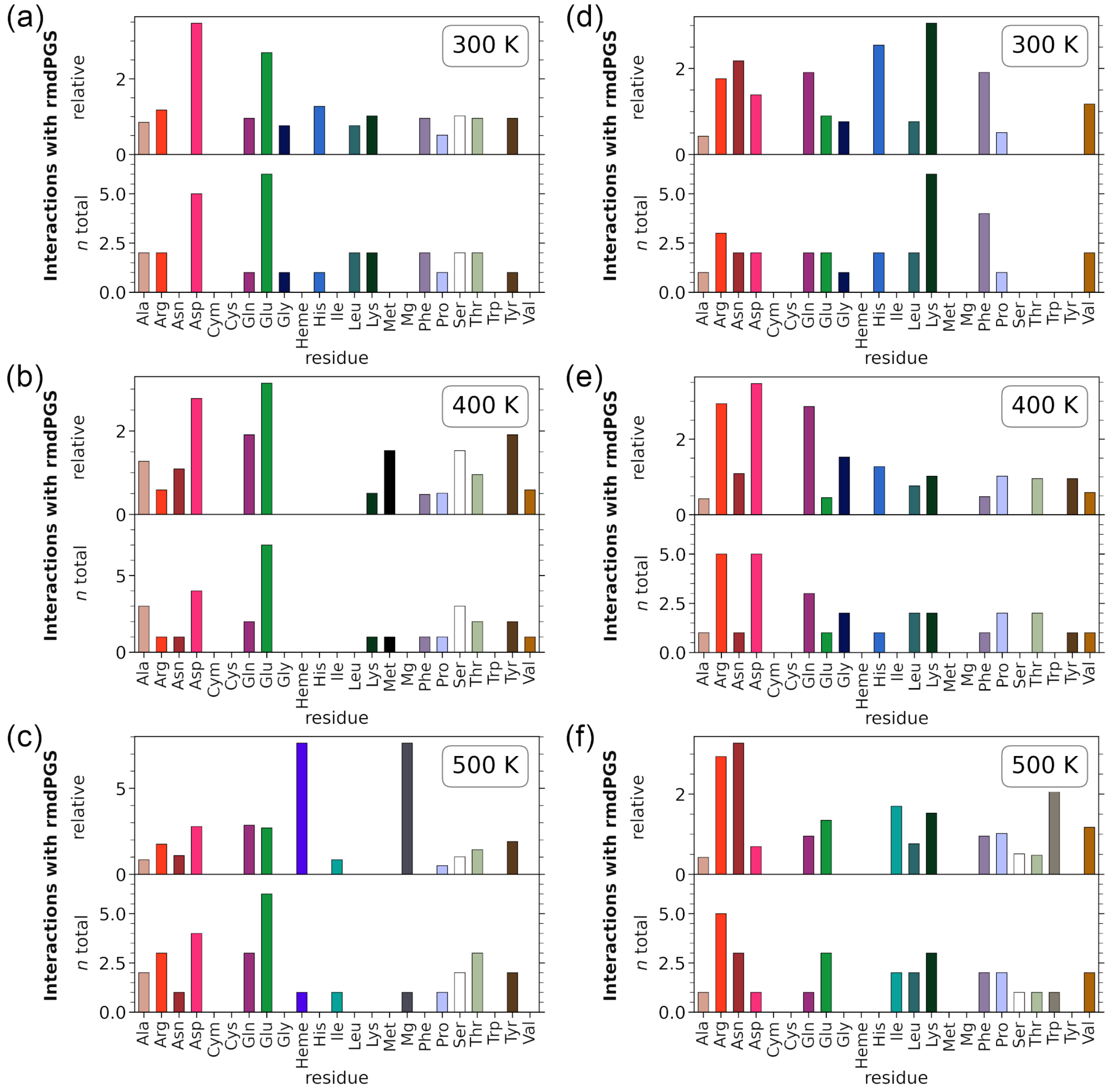

(i) H (ii) OH

Figure S3: SASA interaction bar plots for H and OH with *Cvi*UPO at different temperatures: (a) at 300 K, (b) 400 K, and (c) at 500 K. The bar plots below show the relative and total interactions per amino acid. The heme cofactor (Heme) and the coordinated Mg ion (Mg) are highlighted separately, as is the cysteine group (Cym) bound to the heme center, which is chemically different from a Cys with a free thiol.

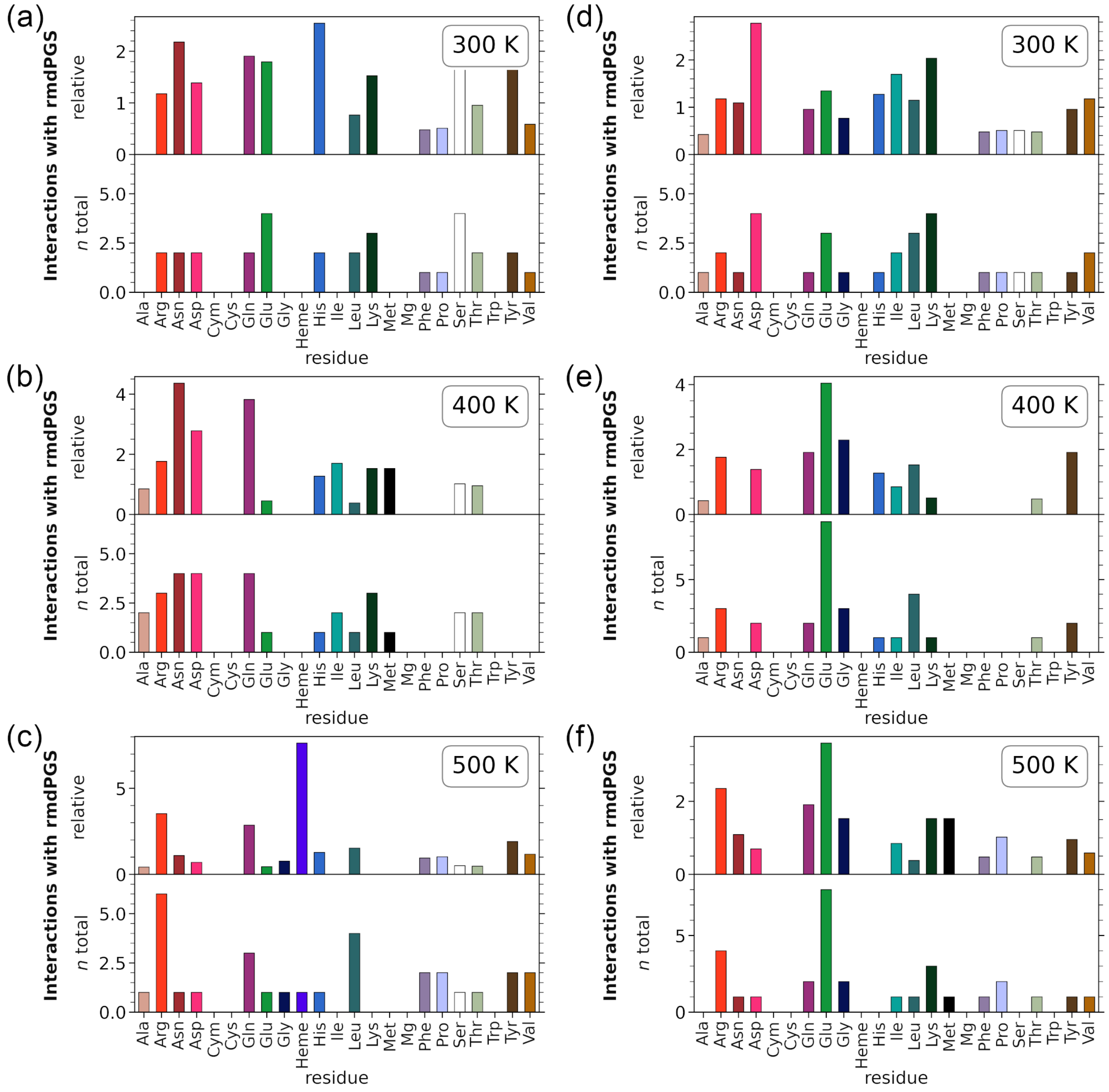

(i) H_2O_2 (ii) NO

Figure S4: SASA interaction bar plots for H_2O_2 and NO with *Cvi*UPO at different temperatures: (a) at 300 K, (b) 400 K, and (c) at 500 K. The bar plots below show the relative and total interactions per amino acid. The heme cofactor (Heme), the coordinated Mg ion (Mg) and the cysteine group bound to the heme (Cym) are highlighted separately.

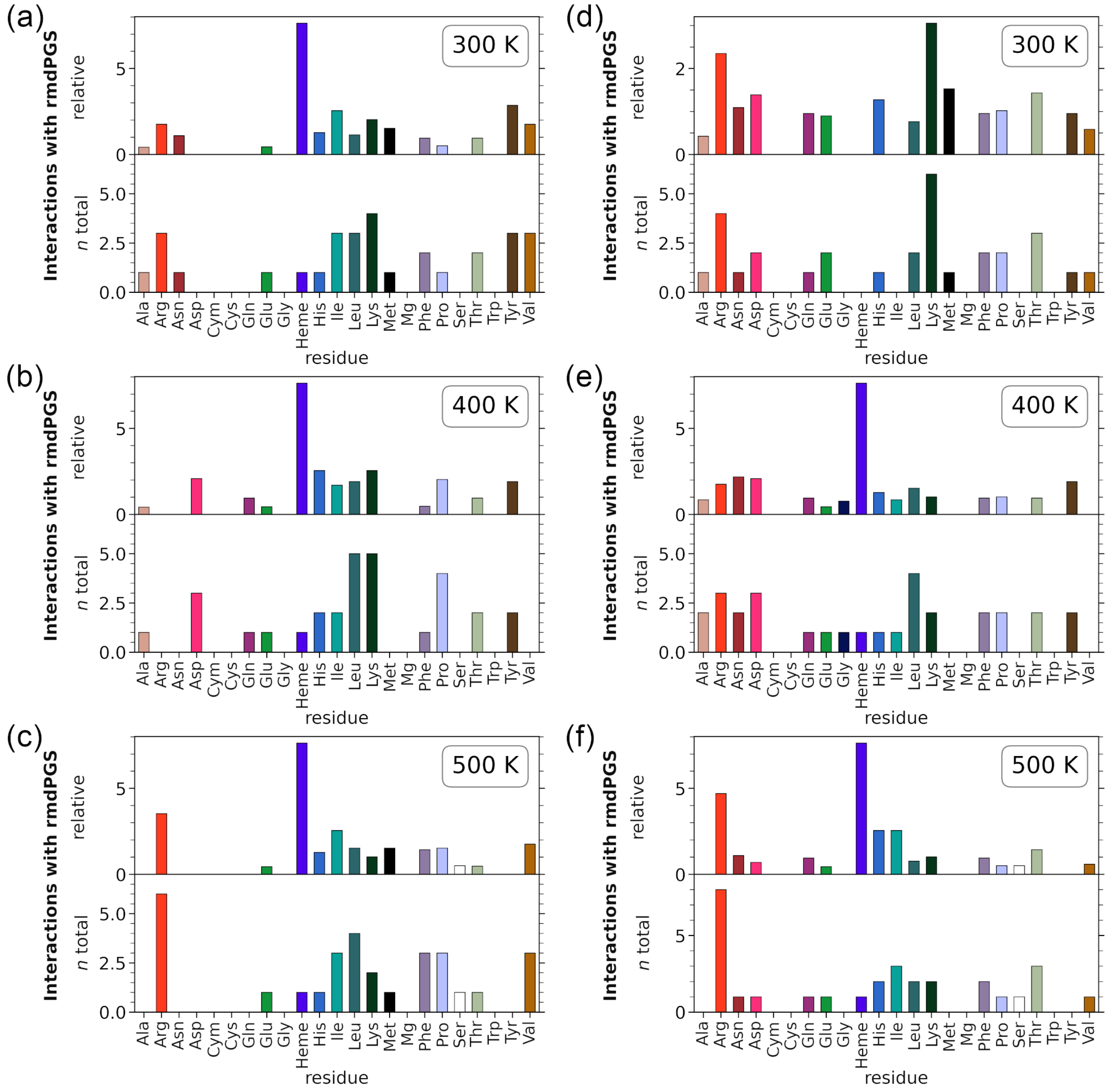

(i) O_2 (ii) O

Figure S5: SASA interaction bar plots for O_2 and O with *Cvi*UPO at different temperatures: (a) at 300 K, (b) 400 K, and (c) at 500 K. The bar plots below show the relative and total interactions per amino acid. The heme cofactor (Heme), the coordinated Mg ion (Mg) and the cysteine group bound to the heme (Cym) are highlighted separately.

Table S1: Data for the short validation MD simulations from Section "SASA interaction analysis *Cvi*UPO" for all tested *rmd*PGS at 300 K in **vacuum**. Of the ten tested SASA positions (with the lowest interaction energies) only those SASA points are in the table listed where an immediate reaction occurred. Therefore, the rows of H_2O_2 and O_2 do not contain any data. The full data set is available and has been provided alongside the main paper.

**H**	**xyz**	**SASA closest residue**	**SASA E_int / eV**	**MD interaction**
	0.025 2.826 -23.8	Glu124	-0.217	OE1 (Glu124)
	-15.765 -12.366 11.894	Asp104	-0.212	OD1 (Asp104)
	-0.381 19.075 1.27	Thr221	-0.207	O (221)
	-13.936 -12.142 12.812	Lys103	-0.202	OD1 (Asp104)
	0.803 1.108 -22.84	Tyr125	-0.201	OH (Tyr125)
	-0.298 20.662 10.614	Leu225	-0.191	O (Leu225)
	10.015 11.442 -7.978	Glu216	-0.186	O (Glu216)
	-11.175 0.619 5.562	Arg96	-0.183	O (Arg96)
**OH**	**xyz**	**SASA closest residue**	**SASA E_int / eV**	**MD interaction**
	1.107 21.172 3.397	Leu225	-0.825	HN (Leu225)
	-4.136 1.205 13.22	Asp91	-0.793	HN (Asp91)
	-2.516 -8.267 19.134	Arg84	-0.728	HE (Arg84)
	2.024 12.392 16.171	Val153	-0.728	HG23 (Val153)
**H2O2**	**xyz**	**SASA closest residue**	**SASA E_int / eV**	**MD interaction**
				no reaction
**NO**	**xyz**	**SASA closest residue**	**SASA E_int / eV**	**MD interaction**
	-6.19 -22.057 -9.084	Thr9	-1.01	HN (Thr9)
**O**	**xyz**	**SASA closest residue**	**SASA E_int / eV**	**MD interaction**
	1.107 21.172 3.397	Leu225	-1.175	HN (Leu225), HN (Ile18)
	-18.717 -3.891 -8.046	Lys114	-1.127	HN (Lys114)
	-6.255 20.747 6.826	Lys224	-1.125	HZ2 (Lys224)
	-6.493 20.708 6.454	Glu223	-1.12	HN (Lys224)
	3.565 -21.510 12.105	Asn37	-1.081	HN (Asn37)
	-14.064 -7.808 -13.948	Lys184	-1.071	HZ2 (Lys184)
**O2**	**xyz**	**SASA closest residue**	**SASA E_int / eV**	**MD interaction**
				no reaction

Table S2: Data for the short validation MD simulations from Section "SASA interaction analysis *Cvi*UPO" for all tested *rmd*PGS at 300 K in **solvent**. Of the ten tested SASA positions (with the lowest interaction energies) only those SASA points are in the table listed where an immediate reaction occurred. Therefore, the rows of H_2O_2 and O_2 do not contain any data. The full data set is available and has been provided alongside the main paper.

**H**	**xyz**	**SASA closest residue**	**SASA E_int / eV**	**MD interaction**
	-0.296 20.962 11.561	Asp227	-0.19	OD2 (Asp227)
**OH**	**xyz**	**SASA closest residue**	**SASA E_int / eV**	**MD interaction**
				no reaction
**H2O2**	**xyz**	**SASA closest residue**	**SASA E_int / eV**	**MD interaction**
	-4.654 3.113 10.773	Gln155	-1.269	HE21 (Gln155)
	-4.425 0.721 13.495	Asp91	-1.146	H (Asp91)
	-3.29 -0.177 13.716	Hish85	-1.081	HE1 (Hish85)
**NO**	**xyz**	**SASA closest residue**	**SASA E_int / eV**	**MD interaction**
	-0.437 -20.604 -15.493	Lys8	-1.018	HG1, CG (Lys8)
	2.763 -21.539 12.001	Arg36	-1.001	HA (Arg36)
**O**	**xyz**	**SASA closest residue**	**SASA E_int / eV**	**MD interaction**
				no reaction
**O2**	**xyz**	**SASA closest residue**	**SASA E_int / eV**	**MD interaction**
	2.79 -21.421 11.992	Thr78	-1.368	HB (Thr78), HG1 (Arg36)
	2.763 -21.539 12.001	Arg36	-1.357	HB (Thr78), HG1 (Arg36)

The results for higher temperatures were excluded from this section to minimize the length of the Supplementary Information. However, the corresponding data are available and have been provided alongside the main paper.

***Aae*UPO**

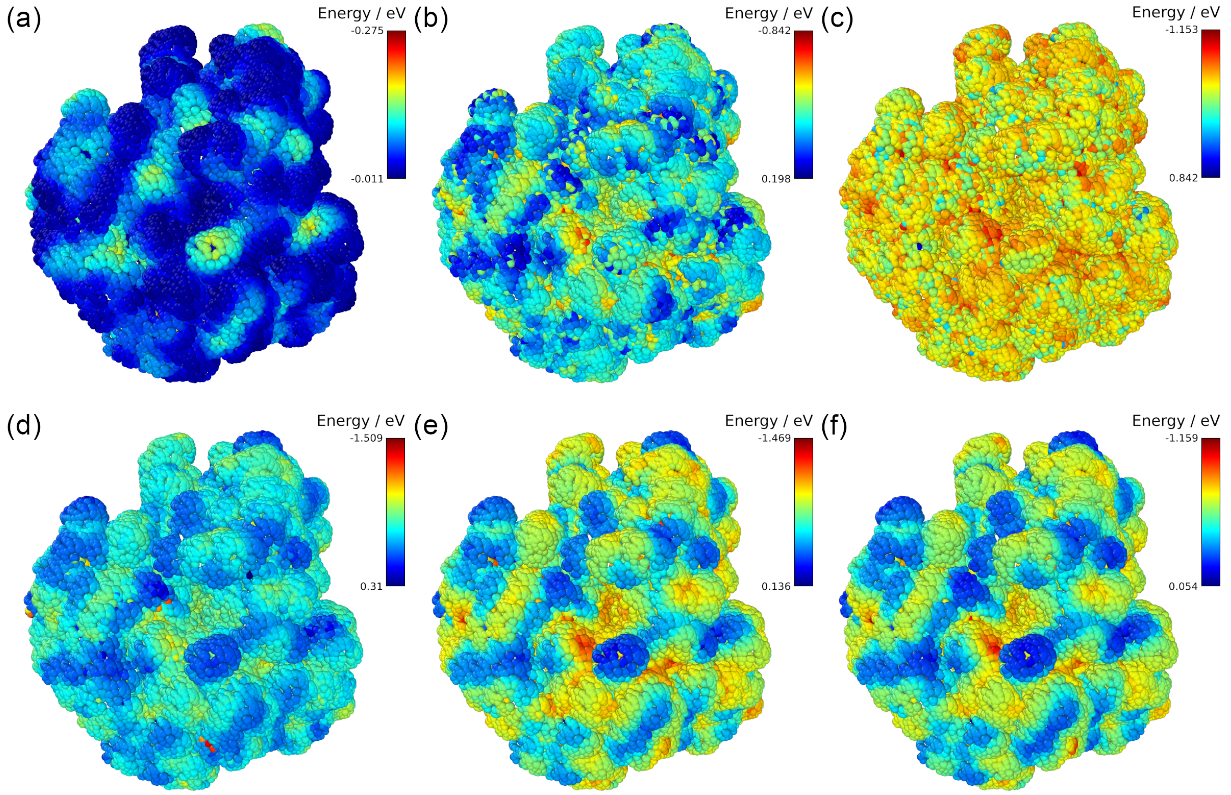

Figure S6: Interaction maps from SASA for the enzyme *Aae*UPO at 300 K. (a) shows the interaction map with H and (b), (c), (d), (e) and (f) show the interaction maps for OH, H_2O_2, NO, O_2 and O, respectively. The colour bar in the upper right corner of each interaction map indicates the value of the interaction energy. More negative values are indicated in red, less negative values in blue.

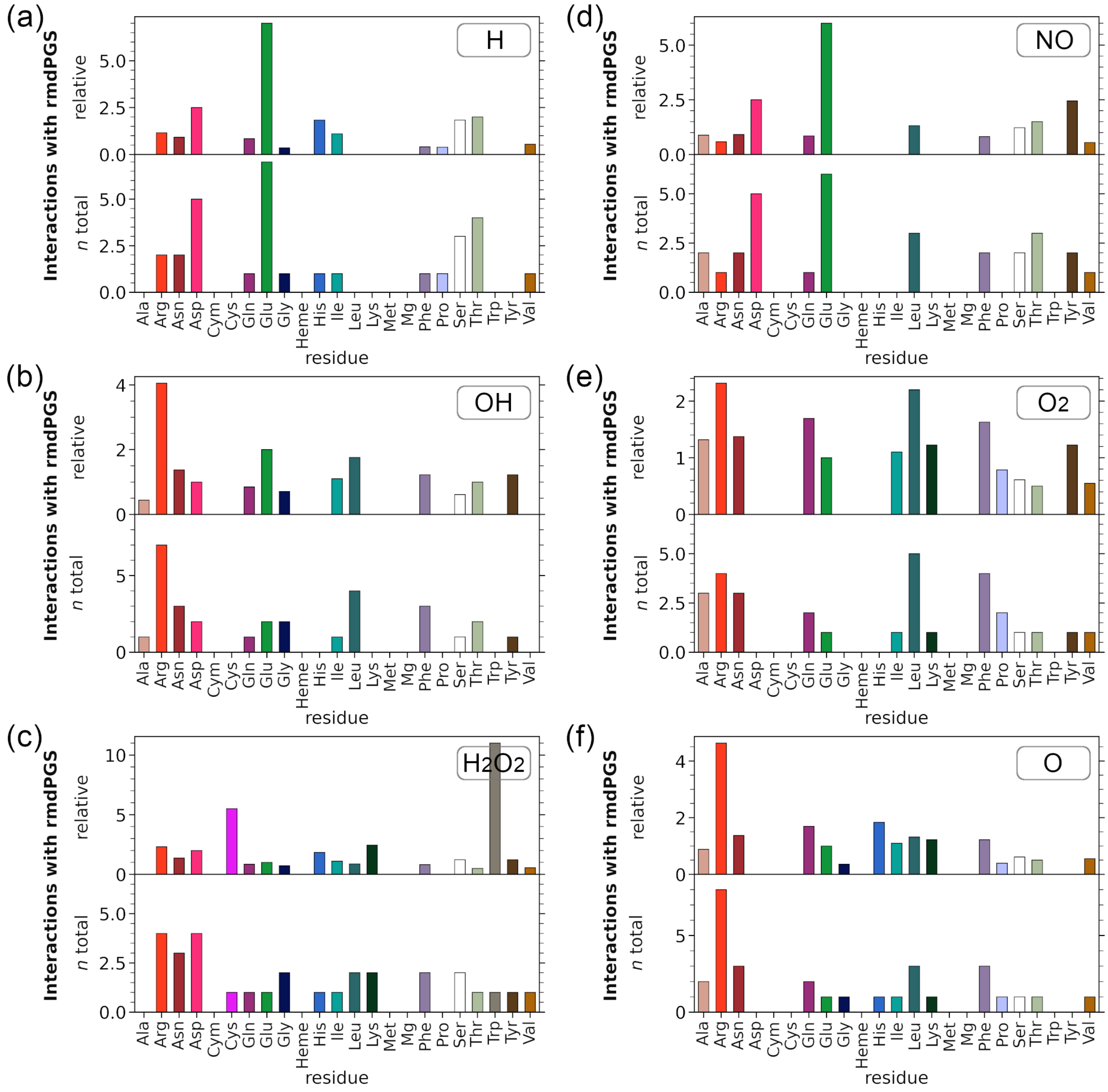

Figure S7: SASA interaction bar plots at 300 K for *Aae*UPO. The bar plots below show the relative and total interactions per amino acid. The heme cofactor (Heme), the coordinated Mg ion (Mg) and the cysteine group bound to the heme (Cym) are highlighted separately. (a) shows the interactions with H and (b), (c), (d), (e) and (f) show the interactions for OH, H_2O_2, NO, O_2 and O, respectively.

Table S3: Data for the short validation MD simulations from Section "Comparison to *Aae*UPO and GapA" for all tested *rmd*PGS at 300 K in **vacuum**. Of the ten tested SASA positions (with the lowest interaction energies) only those SASA points are in the table listed where an immediate reaction occurred. Therefore, the rows of H_2O_2, NO and O_2 do not contain any data. The full data set is available and has been provided alongside the main paper.

**H**	**xyz**	**SASA closest residue**	**SASA E_int / eV**	**MD interaction**
	1.941 -7.982 -26.657	Glu146	-0.275	OE2 (Glu146)
	1.026 -7.549 -26.845	Glu142	-0.247	OE2 (Glu146)
	-2.554 -5.693 19.374	Asp91	-0.223	OD1 (Asp91)
	4.14 14.58 12.59	Gln313	-0.192	O (Phe321)
	-2.328 -8.81 18.342	Thr90	-0.189	O (Thr90)
	3.225 15.249 12.77	Val315	-0.183	O (Phe321)
	-11.902 21.905 -3.468	Ser271	-0.182	O (Ser271)
	14.725 -5.608 8.976	Asn61	-0.180	OD1 (Asn61)
**OH**	**xyz**	**SASA closest residue**	**SASA E_int / eV**	**MD interaction**
	-7.076 6.975 -8.468	Arg189	-0.842	HH21 (Arg189)
	15.438 6.689 6.478	Leu9	-0.832	HN (Leu9)
	12.739 12.509 -13.144	Leu162	-0.804	HN (Leu162)
	-21.881 4.895 -20.556	Ile262	-0.769	HN (Ile262)
**H2O2**	**xyz**	**SASA closest residue**	**SASA E_int / eV**	**MD interaction**
				no reactions
**NO**	**xyz**	**SASA closest residue**	**SASA E_int / eV**	**MD interaction**
				no reactions
**O2**	**xyz**	**SASA closest residue**	**SASA E_int / eV**	**MD interaction**
				no reactions
**O**	**xyz**	**SASA closest residue**	**SASA E_int / eV**	**MD interaction**
	-5.719 20.127 -7.11	Phe274	-1.159	HD1 Phe274
	-21.881 4.895 -20.556	Ile262	-1.137	HN Ile262
	20.19 2.179 9.391	Glu10	-1.118	HN (Glu10), HN (Asn11)
	-12.742 6.762 24.991	Asn328	-1.112	HD2 (Arg301), HD21 (Asn328)
	11.861 13.57 -1.451	Ser240	-1.09	HN (Ser240)
	-5.344 20.631 -6.851	Phe274	-1.057	HN (Phe274), HH21 (Arg257)

No interactions between the *rmd*PGS and *Aae*UPO were detected in the short SASA MDs with solvent.

The results for higher temperatures were excluded from this section to minimize the length of the Supplementary Information. However, the corresponding data are available and have been provided alongside the main paper.

GapA

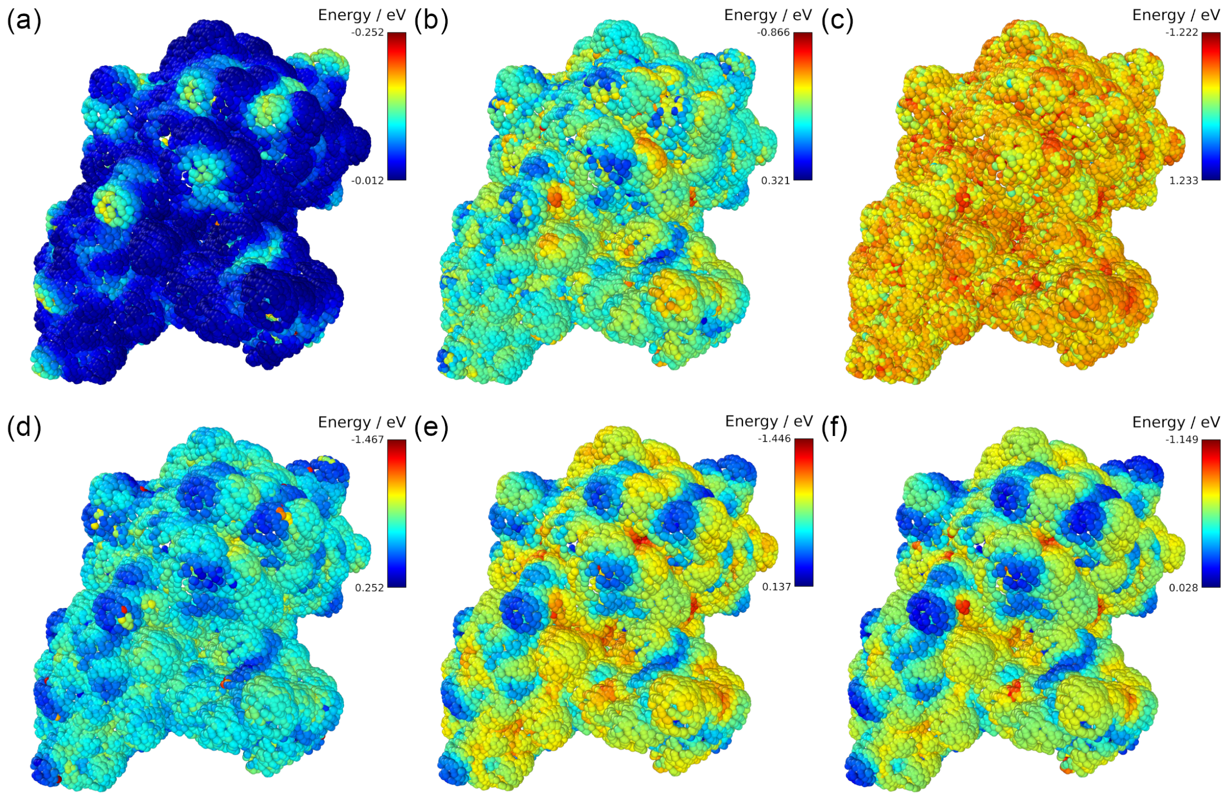

Figure S8: Interaction maps from SASA for the enzyme GapA at 300 K. (a) shows the interaction map with H and (b), (c), (d), (e) and (f) show the interaction maps for OH, H_2O_2, NO, O_2 and O, respectively.The colour bar in the upper right corner of each interaction map indicates the value of the interaction energy. More negative values are indicated in red, less negative values in blue.

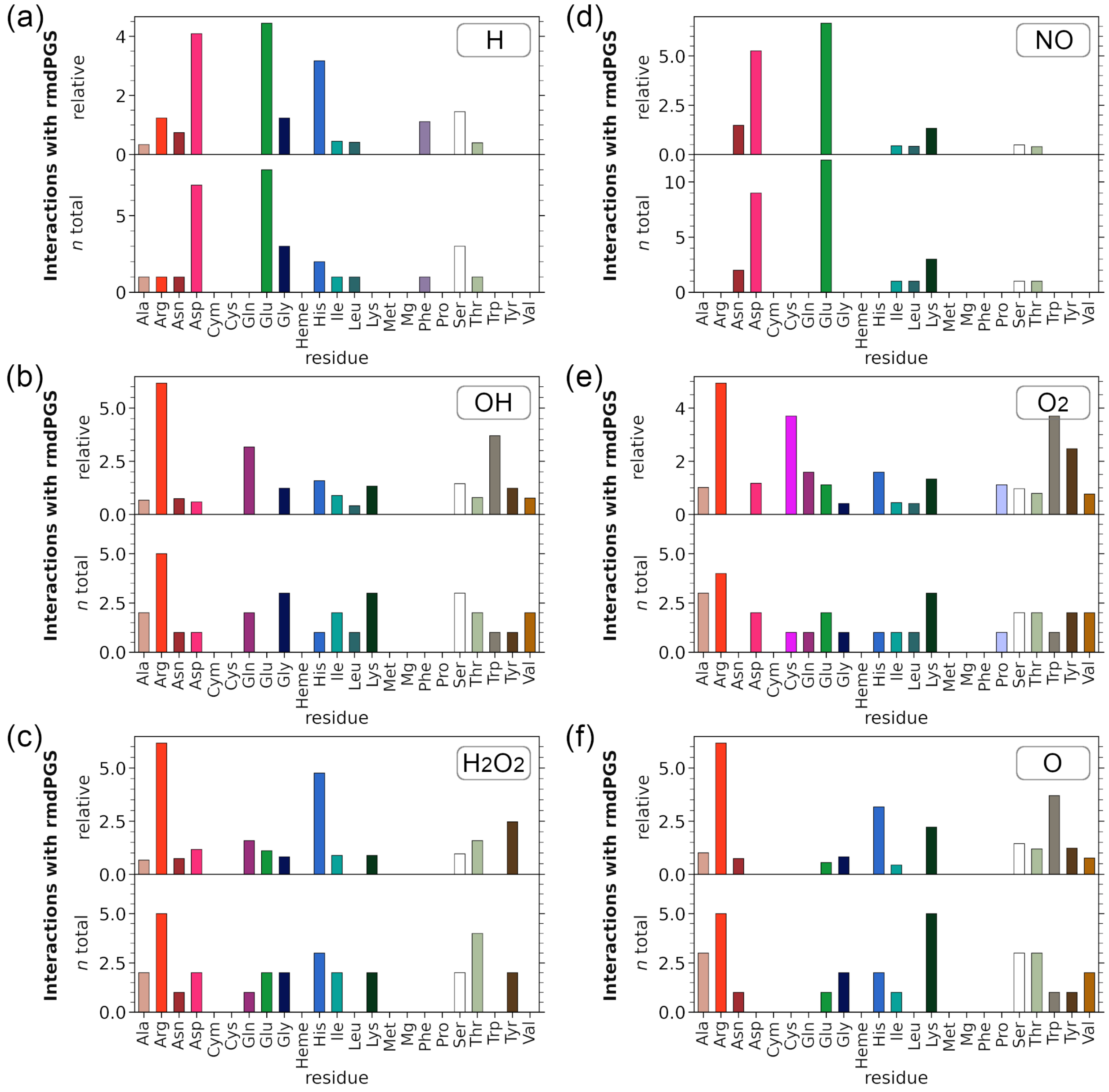

Figure S9: SASA interaction bar plots at 300 K for GapA. The bar plots below show the relative and total interactions per amino acid. The heme cofactor (Heme), the coordinated Mg ion (Mg) and the cysteine group bound to the heme (Cym) are highlighted separately. (a) shows the interactions with H and (b), (c), (d), (e) and (f) show the interactions for OH, H_2O_2, NO, O_2 and O, respectively.

Table S4: Data for the short validation MD simulations from Section "Comparison to *Aae*UPO and GapA" for all tested *rmd*PGS at 300 K in **vacuum**. Of the ten tested SASA positions (with the lowest interaction energies) only those SASA points are in the table listed where an immediate reaction occurred. Therefore, the rows of H_2O_2, NO and O_2 do not contain any data. The full data set is available and has been provided alongside the main paper.

**H**	**xyz**	**SASA closest residue**	**SASA E_int / eV**	**MD interaction**
	-14.174 13.575 -22.124	Glu202	-0.234	OD1 (Asp187)
	16.83 2.245 21.372	Leu333	-0.213	OT1 (Leu333)
	16.632 2.676 21.313	Gly140	-0.211	OT1 (Leu333)
	-11.523 -7.879 2.094	Ser285	-0.207	O (Asp281)
	-3.328 -20.437 -13.4	Thr296	-0.197	O (Thr296)
	-14.856 -16.476 -5.369	Glu276	-0.195	O (Glu276)
	-12.534 -7.989 2.371	Asp281	-0.191	O (Asp281)
	-18.562 18.793 22.722	Asp59	-0.191	OD1 (Asp59)
	-12.437 -7.571 2.860	Ile83	-0.190	O (Ile283)
	20.023 -18.895 -15.649	Glu169	-0.188	OE1 (Glu169)
**OH**	**xyz**	**SASA closest residue**	**SASA E_int / eV**	**MD interaction**
	-2.05 -5.305 12.117	Arg19	-0.807	HH11 (Arg19)
	-12.752 -7.05 -10.723	Ser279	-0.799	HN (Ser279)
	-10.964 13.683 2.709	Arg14	-0.73	HH22 (Arg14)
	-12.16 -7.333 -10.803	Trp312	-0.711	HN (Ser279)
	8.812 22.837 8.457	Lys81	-0.704	HN (Lys81)
	-14.238 4.07 8.004	Arg18	-0.702	HH21 (Arg18)
**H2O2**	**xyz**	**SASA closest residue**	**SASA E_int / eV**	**MD interaction**
				no reactions
**NO**	**xyz**	**SASA closest residue**	**SASA E_int / eV**	**MD interaction**
				no reactions
**O2**	**xyz**	**SASA closest residue**	**SASA E_int / eV**	**MD interaction**
				no reactions
**O**	**xyz**	**SASA closest residue**	**SASA E_int / eV**	**MD interaction**
	-10.109 13.349 2.4	Arg14	-1.097	HH12 (Arg14)
	-12.752 -7.05 -10.723	Ser279	-1.085	HN (Ser279), HG1 Ser279)
	-5.333 24.677 -17.099	Arg190	-1.07	HD2 (Arg190)
	8.637 11.106 -16.115	Arg195	-1.058	HH11 (Arg195)
	8.812 22.837 8.457	Lys81	-1.047	HN (Lys81)
	-12.160 -7.333 -10.803	Trp312	-1.032	HN (Ser279)

No interactions between the *rmd*PGS and GapA were detected in the short SASA MDs with solvent.

The results for higher temperatures were excluded from this section to minimize the length of the Supplementary Information. However, the corresponding data are available and have been provided alongside the main paper.

Interactions with high *rmd*PGS concentrations

*Cvi*UPO

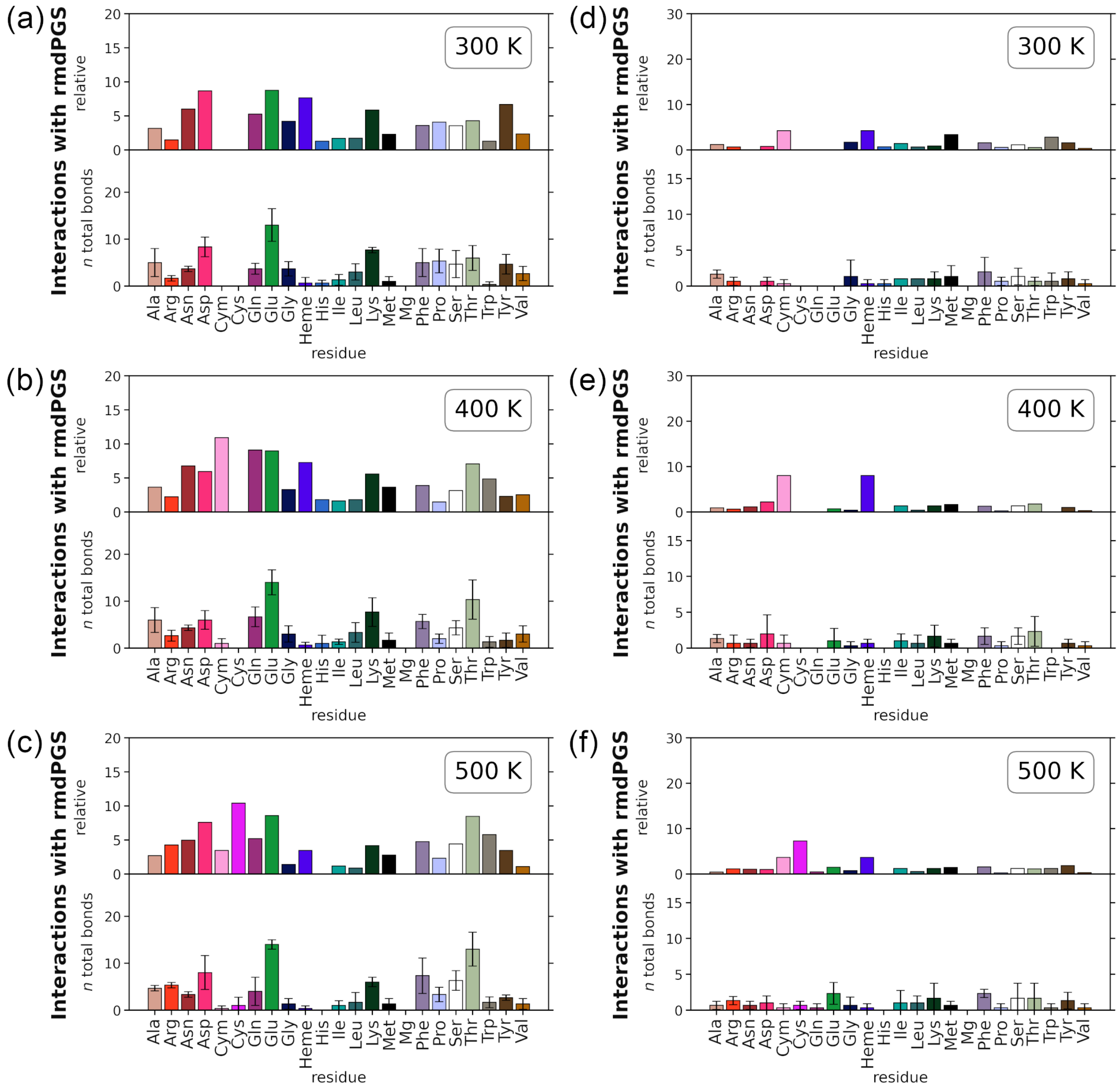

(i) Without solvent

(ii) With solvent

Figure S10: Bond analysis for high concentrations of H with *Cvi*UPO. All panels show the total number of bonds to an additional hydrogen atom per residue and the relative interactions calculated following equation 3. The heme cofactor (Heme), the coordinated Mg ion (Mg) and the cysteine group bound to the heme (Cym) are highlighted separately. The panels (a) and (d) show the interactions at 300 K, without and with solvent, respectively. The panels (b) and (e) show the interactions at at 400 K and the panels (c) and (f) show the interactions at at 500 K

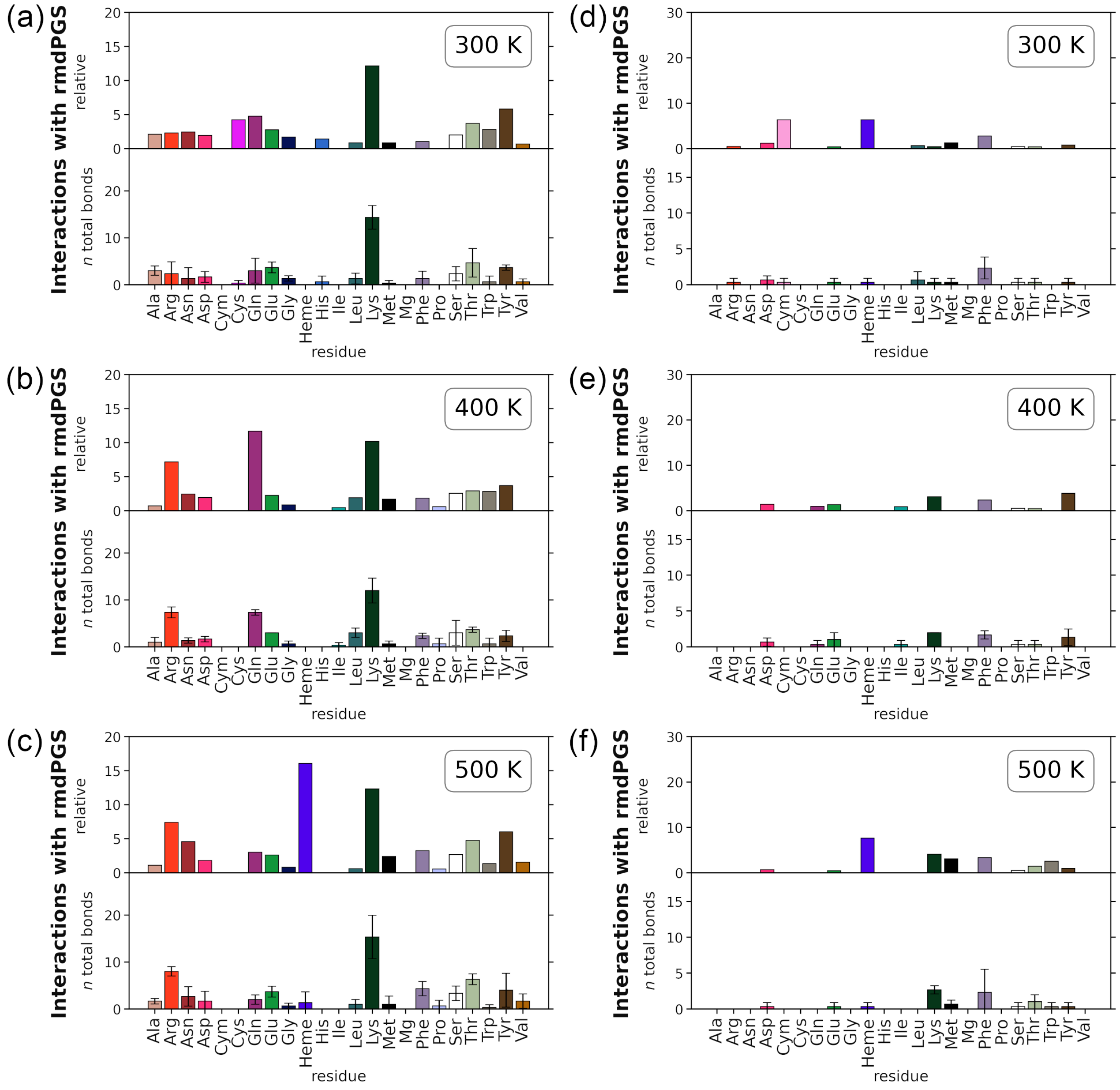

(i) Without solvent

(ii) With solvent

Figure S11: Bond analysis for high concentrations of H_2O_2 with *Cvi*UPO. All panels show the total number of bonds to an additional hydrogen atom per residue and the relative interactions calculated following equation 3. The heme cofactor (Heme), the coordinated Mg ion (Mg) and the cysteine group bound to the heme (Cym) are highlighted separately. The panels (a) and (d) show the interactions at 300 K, without and with solvent, respectively. The panels (b) and (e) show the interactions at at 400 K and the panels (c) and (f) show the interactions at at 500 K

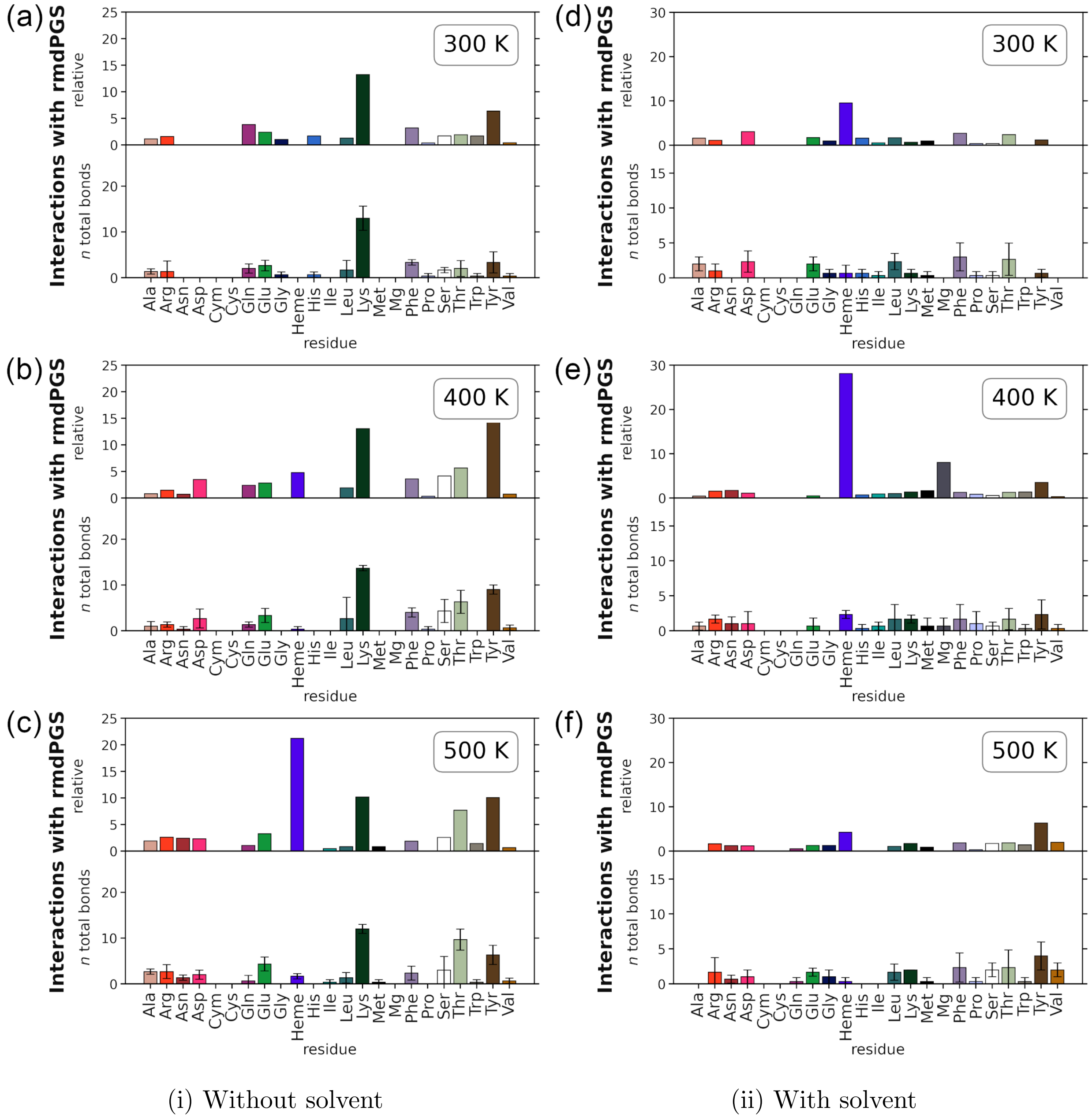

Figure S12: Bond analysis for high concentrations of NO with *Cvi*UPO. All panels show the total number of bonds to an additional hydrogen atom per residue and the relative interactions calculated following equation 3. The heme cofactor (Heme), the coordinated Mg ion (Mg) and the cysteine group bound to the heme (Cym) are highlighted separately. The panels (a) and (d) show the interactions at 300 K, without and with solvent, respectively. The panels (b) and (e) show the interactions at at 400 K and the panels (c) and (f) show the interactions at at 500 K

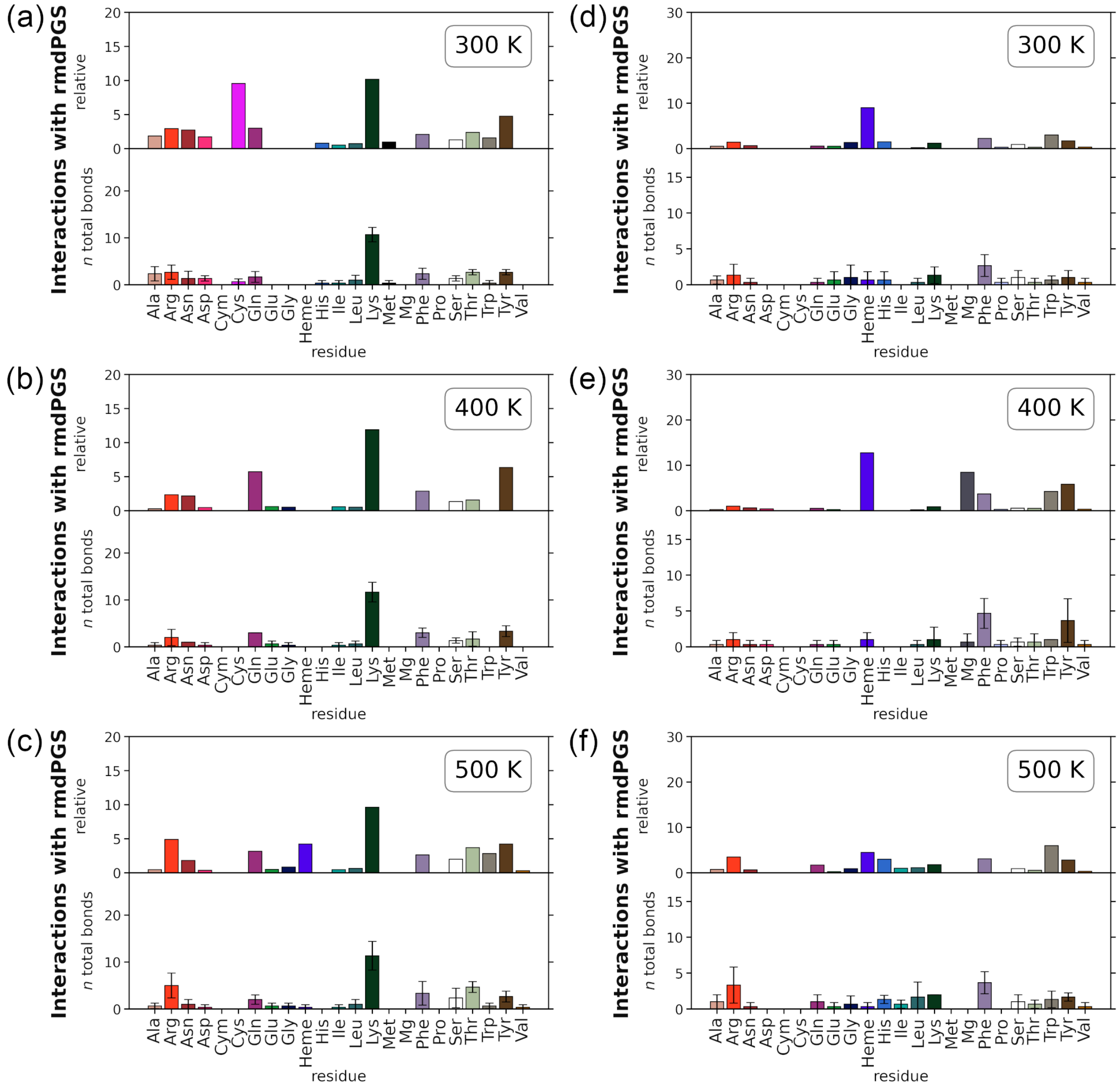

(i) Without solvent (ii) With solvent

Figure S13: Bond analysis for high concentrations of O_2 with *Cvi*UPO. All panels show the total number of bonds to an additional hydrogen atom per residue and the relative interactions calculated following equation 3. The heme cofactor (Heme), the coordinated Mg ion (Mg) and the cysteine group bound to the heme (Cym) are highlighted separately. The panels (a) and (d) show the interactions at 300 K, without and with solvent, respectively. The panels (b) and (e) show the interactions at at 400 K and the panels (c) and (f) show the interactions at at 500 K

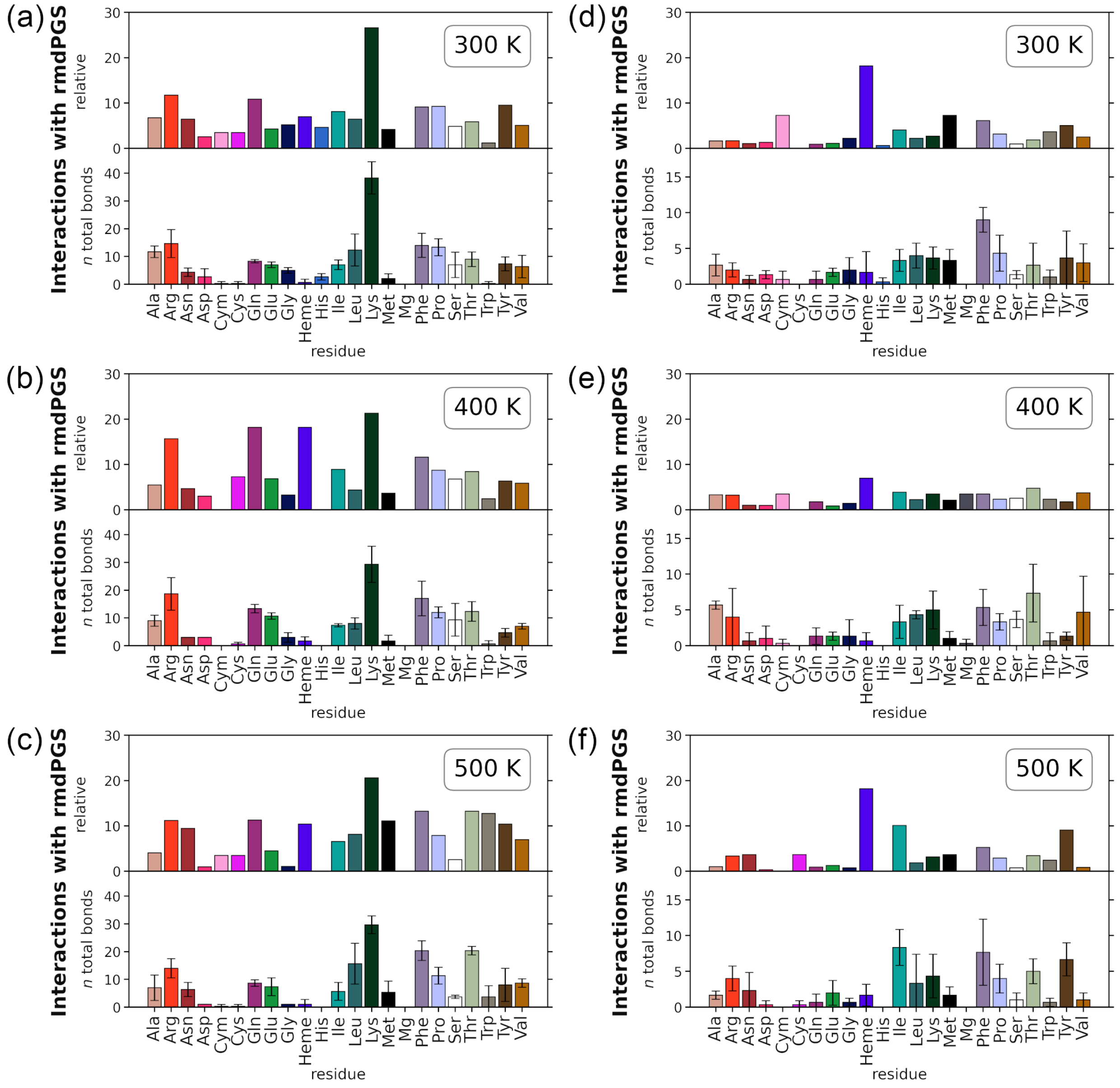

(i) Without solvent (ii) With solvent

Figure S14: Bond analysis for high concentrations of oxygen with *Cvi*UPO. All panels show the total number of bonds to an additional hydrogen atom per residue and the relative interactions calculated following equation 3. The heme cofactor (Heme), the coordinated Mg ion (Mg) and the cysteine group bound to the heme (Cym) are highlighted separately. The panels (a) and (d) show the interactions at 300 K, without and with solvent, respectively. The panels (b) and (e) show the interactions at at 400 K and the panels (c) and (f) show the interactions at at 500 K

***Aae*UPO**

The interaction behavior of the *rmd*PGS with *Aae*UPO shows more pronounced differences to the SASA predictions. While the SASA analysis showed various interactions between PGS and Arg, these are much reduced in the MD simulations. It is particularly striking that all PGS show a strong tendency towards proline (Pro) in the MD simulations, which might be due to the fact that Pro is the second most frequent amino acid in the protein. Similar to the other two enzymes temperature increase does not seem to change the behavior significantly. Interestingly the interaction profiles of the solvent simulations of *Aae*UPO are closer to the SASA predictions than the vacuum results. One explanation for this could be that the PGS in the solvent have the opportunity to diffuse to their preferred interaction partners. Nevertheless, the results should also be treated with caution here, as the error bars are still very high.

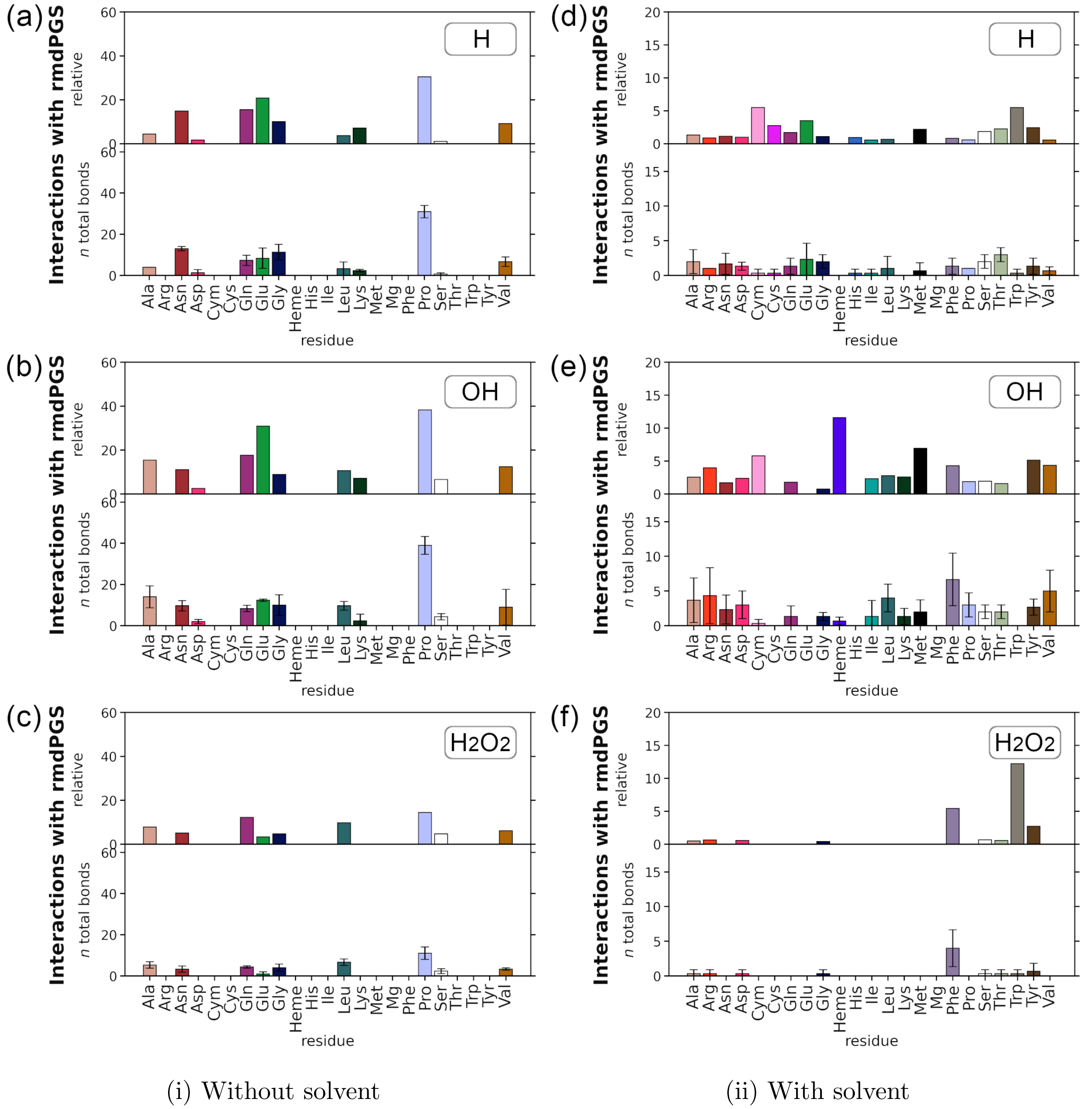

Figure S15: Bond analysis for high concentrations of *rmd*PGS with *Aae*UPO at 300 K. All panels show the total number of bonds to an additional hydrogen atom per residue and the relative interactions calculated following equation 3. The heme cofactor (Heme), the coordinated Mg ion (Mg) and the cysteine group bound to the heme (Cym) are highlighted separately. (a) shows the bonds to H without solvent while (b) and (c) show the bonds to OH, H_2O_2. The panel (d) shows the bonds to H with solvent. (b) and (c) show the bonds with solvent for OH, H_2O_2, respectively.

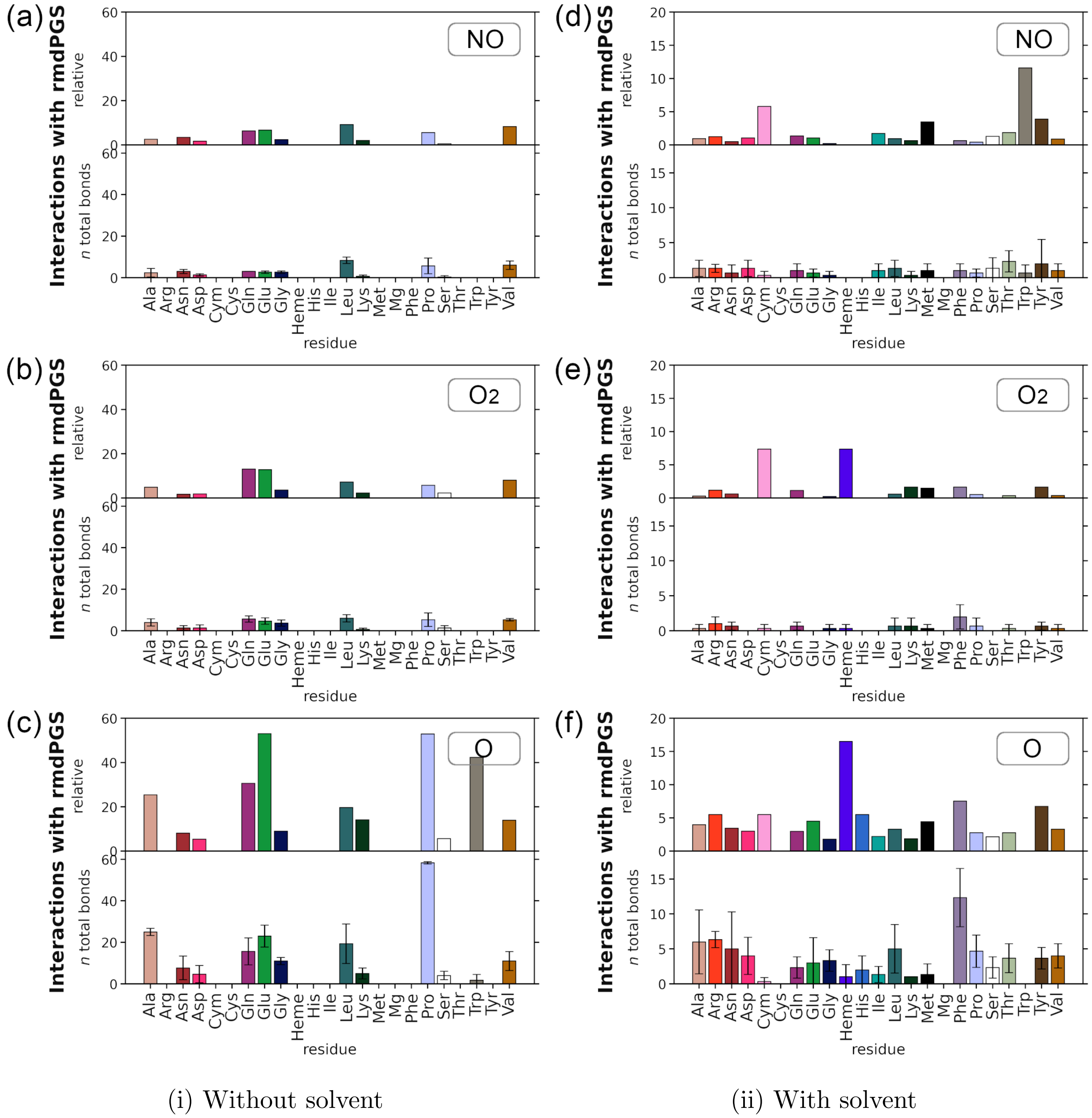

Figure S16: Bond analysis for high concentrations of *rmd*PGS with *Aae*UPO at 300 K. All panels show the total number of bonds to an additional hydrogen atom per residue and the relative interactions calculated following equation 3. The heme cofactor (Heme), the coordinated Mg ion (Mg) and the cysteine group bound to the heme (Cym) are highlighted separately. (a) shows the bonds to NO without solvent while (b) and (c) show the bonds to O_2, O. The panel (d) shows the bonds to NO with solvent.(b) and (c) show the bonds with solvent for O_2, O, respectively.

The results for higher temperatures were excluded from this section to minimize the

length of the Supplementary Information. However, the corresponding data are available and have been provided alongside the main paper

GapA

For GapA, the MD simulations also follow the trend of the SASA predictions. For most *rmd*PGS the results of the MD simulations look very similar with slight changes in the ratios. Overall, Lys is the most attacked amino acid for all tested *rmd*PGS, followed by Thr and Arg. Interestingly, NO interacts with more residues in the MD simulations than predicted by the SASA analysis, although the variety of different residues is smaller compared to the other *rmd*PGS. As with *Cvi*UPO, the solvent results are even closer to the SASA predictions.

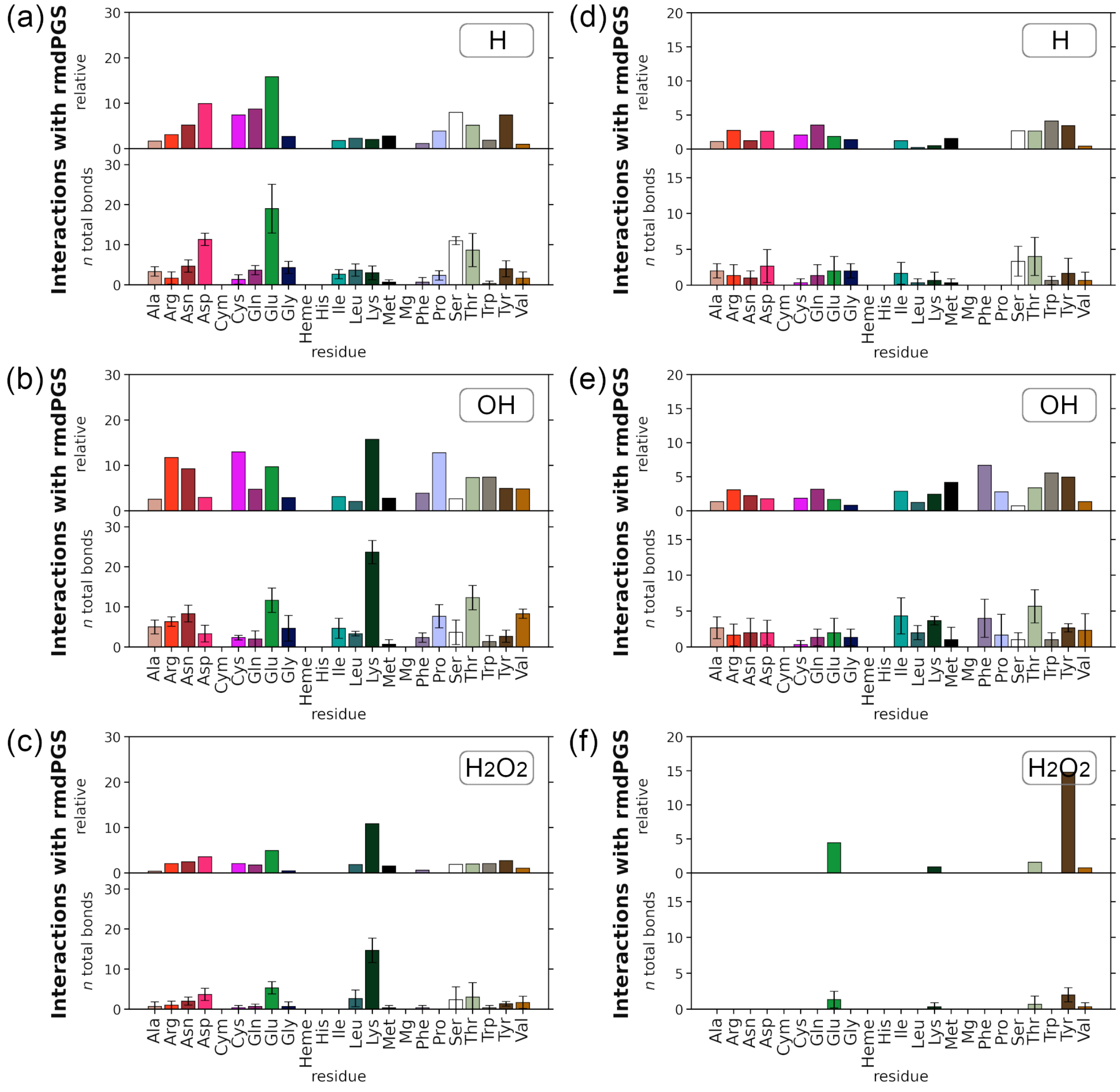

(i) Without solvent

(ii) With solvent

Figure S17: Bond analysis for high concentrations of *rmd*PGS with GapA at 300 K. All panels show the total number of bonds to an additional hydrogen atom per residue and the relative interactions calculated following equation 3. The heme cofactor (Heme), the coordinated Mg ion (Mg) and the cysteine group bound to the heme (Cym) are highlighted separately. (a) shows the bonds to H without solvent while (b) and (c) show the bonds to OH, H_2O_2. The panel (d) shows the bonds to H with solvent. (b) and (c) show the bonds with solvent for OH, H_2O_2, respectively.

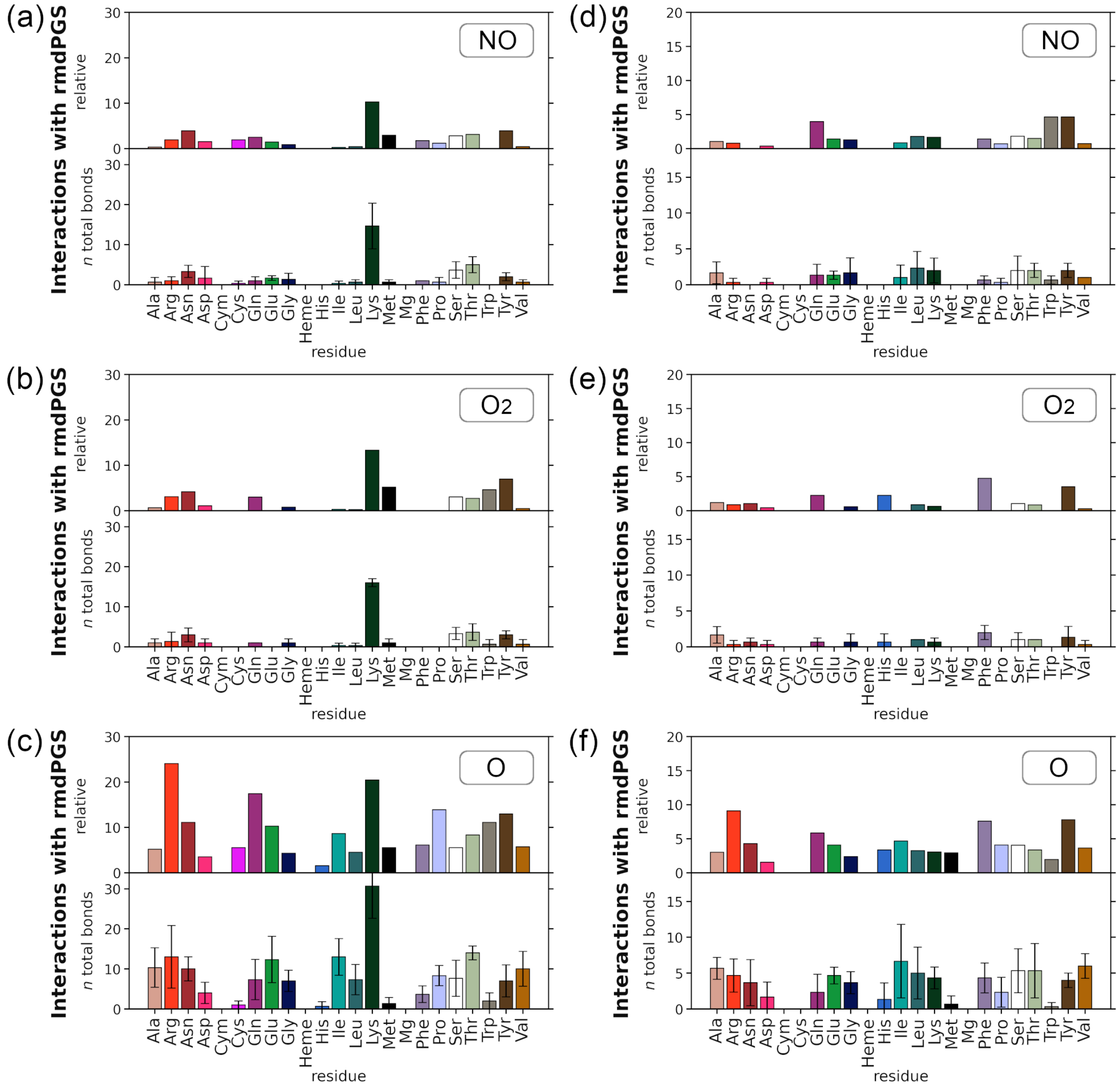

(i) Without solvent

(ii) With solvent

Figure S18: Bond analysis for high concentrations of *rmd*PGS with GapA at 300 K. All panels show the total number of bonds to an additional hydrogen atom per residue and the relative interactions calculated following equation 3. The heme cofactor (Heme), the coordinated Mg ion (Mg) and the cysteine group bound to the heme (Cym) are highlighted separately. (a) shows the bonds to NO without solvent while (b) and (c) show the bonds to O_2, O. The panel (d) shows the bonds to NO with solvent. (b) and (c) show the bonds with solvent for O_2, O, respectively.

The results for higher temperatures were excluded from this section to minimize the

length of the Supplementary Information. However, the corresponding data are available and have been provided alongside the main paper

References

(1) Monti, S.; Corozzi, A.; Fristrup, P.; Joshi, K. L.; Shin, Y. K.; Oelschlaeger, P.; Duin, A. C. V.; Barone, V. Exploring the conformational and reactive dynamics of biomolecules in solution using an extended version of the glycine reactive force field. *Physical Chemistry Chemical Physics* **2013**, *15*, 15062–15077.

(2) Duin, A. C. V.; Dasgupta, S.; Lorant, F.; Goddard, W. A. ReaxFF: A Reactive Force Field for Hydrocarbons. *Journal of Physical Chemistry A* **2001**, *105*, 9396–9409.

(3) Mortier, W. J.; Van Genechten, K.; Gasteiger, J. Electronegativity equalization: application and parametrization. *Journal of the American Chemical Society* **1985**, *107*, 829–835.

(4) Senftle, T. P.; Hong, S.; Islam, M. M.; Kylasa, S. B.; Zheng, Y.; Shin, Y. K.; Junkermeier, C.; Engel-Herbert, R.; Janik, M. J.; Aktulga, H. M.; et al. The ReaxFF reactive force-field: development, applications and future directions. *npj Computational Materials* **2016**, *2*, 1–14.

(5) Zhang, W.; Duin, A. C. V. Improvement of the ReaxFF Description for Functionalized Hydrocarbon/Water Weak Interactions in the Condensed Phase. *Journal of Physical Chemistry B* *122*, 4083–4092.
